# The oral microbiota is a reservoir for antimicrobial resistance: resistome and phenotypic resistance characteristics of oral biofilm in health, caries, and periodontitis

**DOI:** 10.1186/s12941-023-00585-z

**Published:** 2023-05-13

**Authors:** A. C. Anderson, C. von Ohle, C. Frese, S. Boutin, C. Bridson, K. Schoilew, S. A. Peikert, E. Hellwig, K. Pelz, A. Wittmer, D. Wolff, A. Al-Ahmad

**Affiliations:** 1 Department of Operative Dentistry and Periodontology, Medical Center, Faculty of Medicine, University of Freiburg, Hugstetter Straße 55, 79106 Freiburg, Germany; 2 Department of Conservative Dentistry, Periodontology and Endodontology, University Centre of Dentistry, Oral Medicine and Maxillofacial Surgery, University Hospital Tübingen, Tübingen, Germany; 3 Department of Conservative Dentistry, Clinic for Oral, Dental and Maxillofacial Diseases, University Hospital Heidelberg, Heidelberg, Germany; 4 Department of Infectious Diseases, Medical Microbiology and Hygiene, Heidelberg University Hospital, Heidelberg, Germany; 5 Translational Lung Research Center Heidelberg (TLRC), German Center for Lung Research (DZL), Heidelberg, Germany; 6 Institute of Medical Microbiology and Hygiene, Faculty of Medicine, University of Freiburg, Freiburg, Germany

## Abstract

**Background:**

Antimicrobial resistance (AMR) is an ever-growing threat to modern medicine and, according to the latest reports, it causes nearly twice as many deaths globally as AIDS or malaria. Elucidating reservoirs and dissemination routes of antimicrobial resistance genes (ARGs) are essential in fighting AMR. Human commensals represent an important reservoir, which is underexplored for the oral microbiota. Here, we set out to investigate the resistome and phenotypic resistance of oral biofilm microbiota from 179 orally healthy (H), caries active (C), and periodontally diseased (P) individuals (TRN: DRKS00013119, Registration date: 22.10.2022). The samples were analysed using shotgun metagenomic sequencing combined, for the first time, with culture technique. A selection of 997 isolates was tested for resistance to relevant antibiotics.

**Results:**

The shotgun metagenomics sequencing resulted in 2,069,295,923 reads classified into 4856 species-level OTUs. PERMANOVA analysis of beta-diversity revealed significant differences between the groups regarding their microbiota composition and their ARG profile. The samples were clustered into three ecotypes based on their microbial composition. The bacterial composition of H and C samples greatly overlapped and was based on ecotypes 1 and 2 whereas ecotype 3 was only detected in periodontitis. We found 64 ARGs conveying resistance to 36 antibiotics, particularly to tetracycline, macrolide-lincosamide-streptogramin, and beta-lactam antibiotics, and a correspondingly high prevalence of phenotypic resistance. Based on the microbiota composition, these ARGs cluster in different resistotypes, and a higher prevalence is found in healthy and caries active than in periodontally diseased individuals. There was a significant association between the resistotypes and the ecotypes. Although numerous associations were found between specific antibiotic resistance and bacterial taxa, only a few taxa showed matching associations with both genotypic and phenotypic analyses.

**Conclusions:**

Our findings show the importance of the oral microbiota from different niches within the oral cavity as a reservoir for antibiotic resistance. Additionally, the present study showed the need for using more than one method to reveal antibiotic resistance within the total oral biofilm, as a clear mismatch between the shotgun metagenomics method and the phenotypic resistance characterization was shown.

**Supplementary Information:**

The online version contains supplementary material available at 10.1186/s12941-023-00585-z.

## Background

Antimicrobial resistance (AMR), which according to most recent analyses [1] is responsible for an estimated 1.2 million deaths per year globally, presents a growing threat to public health concerning the successful treatment of bacterial infections. In the EU alone, the estimated annual cost due to these infections in health care and productivity loss add up to EUR 1.5 billion [2]. To tackle AMR, it is necessary to determine where it develops and to elucidate the reservoirs of antimicrobial resistance genes (ARGs), also within the human microbiome. Various sources and routes of dissemination of AMR have been identified, e.g., from environmental, animal, as well as human reservoirs and hospital environments [3, 4]. Fundamentally, all these reservoirs are interconnected through the food chain [3, 4].

Mutations on chromosomal genes for one, but especially the transfer of ARGs located on mobile genetic elements can equip bacteria with resistance and add to the number of resistant strains [5, 6]. Therefore, communities of human commensals can act as reservoirs for resistance genes that can be passed on between different species [7, 8]. The oral microbiota represents such a diverse microscale ecosystem. Approximately 1,000 different microbial taxa are capable of colonizing the oral cavity, of which a partial quantity is found in particular oral niches, e.g., dental plaque [9]. Here, the microbial cells are organized as a biofilm whose characteristics, e.g., close proximity and polymicrobial nature, enable interactions such as horizontal gene transfer [10, 11]. In health, stable homeostasis of the oral microbiota provides benefits for the host and its microbiota. However, environmental factors can tilt that balance towards a dysbiotic change, thereby promoting oral diseases such as caries and periodontitis. These are characterized by bacterial taxa possessing pathogenic potential and specific differences in the microbiota compared to health [9, 12, 13]. However, to date, it has not been analysed whether these compositional differences come into play regarding the resistome. The healthy oral microbiota reportedly contains a wealth of resistance genes [14] and many are located on mobile genetic elements and have been acquired through this process [6, 14]. In terms of the spread of ARGs by the transmission of bacteria, it is significant that the oral cavity is not a closed system and transmission of oral microbes to the gut has been reported to be widespread and even increased for opportunistic pathogens [15]. Oral bacteria can also spread to other body sites via the bloodstream or be passed on to other individuals [16]. Furthermore, due to the sloughing process of oral biofilms formed on hard tissue, flocs of the biofilm may be transmitted to other areas of the human body.

Therefore, we aimed to determine whether the oral microbiota represents a source of ARGs and how the resistome differs in healthy individuals (H) compared to caries active individuals (C) and chronic periodontitis patients (P). Additionally, the integration of genotypic and phenotypic methods is useful, as metagenomics enables the characterisation of a broad range of ARGs, as well as an assessment of abundance and diversity, while the culture technique tests whether there is a phenotypic expression of the ARGs. Given the importance of integrating both methods, it is useful to understand the overlap between the methods in assessing resistance, and whether there is a greater similarity between the methods for certain types of antibiotics. For this reason, we used a combined approach of a shotgun metagenomic analysis to generate comprehensive data on the microbiome and resistome as well as the culture technique to determine phenotypic resistance and link it to the corresponding microbial species. This study hypothesized that the oral biofilm depicts a diverse reservoir for antibiotic resistance. Additionally, we proposed that the antibiotic resistance pattern differs in conditions characterized by health, caries, and periodontitis.

## Materials and methods

### Study design and sampling procedures

In this multicentre trial, a total of 180 participants were recruited at three University hospitals in Southern Germany (Dep. of Operative Dentistry and Periodontology, Freiburg University Hospital; the Dep. of Conservative Dentistry, Heidelberg University Hospital, and the Dep. of Conservative Dentistry, Periodontology and Endodontology, Tübingen University Hospital). They were divided into three groups according to oral health status: healthy (H, DMFT = 0, periodontally healthy [17]), caries active (C, minimum of two dentine carious lesions and two restorative treatments within the last 2 years), and periodontally diseased (P, generalized moderate or severe chronic periodontitis [18]). We specified that healthy participants had to display 28 caries- and restoration-free teeth. In the other two groups, there was no specification concerning the total tooth number or distribution in the mouth. The descriptive data in Table 1 shows that the caries group had 1.4 (SD 2.4) missing teeth and that the periodontitis group had 3.8 (SD 4.1) missing teeth. According to the data, all groups included participants with adequate dental status. Ethical approval was obtained from the local Ethics Committees of the three Medical Faculties (604/16; S-652/2016; 863/201BO2). The study was registered in the German Clinical Trials Register (DRKS00013119). Subjects between 18 and 80 years. were eligible for study participation provided they were in good general health. The exclusion criteria included antibiotic intake within 6 months prior to and during the study, pregnancy or lactation, diabetes, pathological alteration of the oral mucosa, systemic disease, or intake of medication having side effects on gingiva, and restraints that did not permit the performance of daily oral hygiene measures. In the P group, patients were excluded when anti-infective periodontal therapy and subgingival instrumentation took place during the last 24 months. All participants gave their written informed consent. Clinical investigators underwent training and calibration in clinical examination and sampling. The study participants underwent detailed clinical oral examinations including anamnesis and dental and periodontal status. The caries experience was assessed by means of the Decayed-, Missing-, Filled teeth index (DMFT) according to WHO guidelines [17]. Periodontal status was assessed by measuring the probing pocket depth and clinical attachment levels at six sites per tooth using a millimeter scaled periodontal probe. Bleeding on probing was recorded for the measured sites. Gingival and periodontal diseases and conditions were assessed by bleeding on probing (BOP in %), plaque index (PI [19]), and gingiva index (GI [20]) and classified according to Armitage [18]. With regard to possible influencing factors of the oral biofilm, the intake of antibiotics, probiotics, or antiseptic mouth rinses was queried. Furthermore, dietary habits, smoking, number of dental visits, and brush replacements per year as well as qualitative and quantitative saliva parameters (pH and saliva flow rate ml/min) were collected. The participants were asked to abstain from oral hygiene for 24 h and to refrain from eating/drinking for 1 h prior to the examination. Unstimulated saliva was collected in a sterile tube for 5 min directly after the collection of the dental plaque and the pH values, buffering capacities, and saliva flow rates were analyzed (Saliva-Check Buffer, GC Corp.).

**Table 1:** Descriptive information in relative frequencies, mean, and standard deviation (SD) of the study population for the three groups healthy participants (H), caries patients (C), and periodontitis patients (P)

	Healthy n = 63		Caries n = 61		Periodontitis n = 55	
		(SD)		(SD)		(SD)
Age range	25.4 (18–57)	6.9	31.5 (18–71)	10.2	54.8 (26–76)	12.4
Gender [%]						
Female	60.3	–	31.1	–	41.8	-
Male	39.7	–	68.9	–	58.2	-
Body mass index	21.7	2.6	25.2	6.6	26.5	4.3
Social status [%]						
Employees without vocational training	1.6		22.0		9.8	
Employees with completed vocational training	9.8		30.5		68.6	
Academic	88.6		47.5		21.6	
Dental visits [year]	1.5	0.6	1.4	1.8	1.4	1.1
Number of brush replacements [year]	5.3	2.9	6.1	4.9	6.1	3.2
Use of chlorhexidine [%]						
Yes	6		5		17	
No	94		95		83	
Sugar consumption per day [%]						
Never	5		3		9	
Once daily	25		9		28	
Several times daily	70		88		63	
Use of probiotics [%]						
Yes	5		7		8	
No	95		93		92	
Smoking status [%]						
Non-smoker	88		61		60	
Former smoker	5		3		15	
Smoker	7		36		25	
Antibiotics during the last 12 months [%]						
Yes	17		15		19	
No	83		85		81	
Gingiva bleeding index [%]	8.2	9.8	18.9	22.7	24.2	17.5
Bleeding on probing [%]	8.6	9.4	18.2	19.6	41.7	22.2
DMF-T index [n]						
Decayed teeth (D)	0	0	5.4	4.3	0	0
Missing teeth (M)	0	0	1.4	2.4	3.8	4.1
Filled teeth (F)	0	0	7.2	4.1	11.0	5.5
Sum (DMF-T)	0	0	14.1	5.1	14.1	6.2
Saliva pH category [%]						
Acidic (pH-value 5.0–5.9)	0		8		4	
Moderate (pH-value 6.0–6.7)	17		38		22	
Neutral (pH-value 6.8–7.8)	83		54		74	
Saliva flow rate [%]						
Low (< 0.7 ml/min)	0		2		2	
Moderate (0.7–1.0 ml/min)	7		10		11	
High (> 1.0 ml/min)	93		88		87	
Periodontal and gingival status [%]						
Periodontal and gingival health	12.9		5.0		–	
Localized gingivitis	80.6		71.7		–	
Generalized gingivitis	6.5		23.3		–	
Generalized moderate, localized severe chronic periodontitis	–		–		92.3	
Generalized severe chronic periodontitis	–		–		7.2	

*SD* standard deviation

Supragingival plaque samples were taken from healthy and caries active individuals (at least one tooth per quadrant) with a sterile curette and pooled. Subgingival plaque from the periodontitis patients was obtained from 2–3 pockets between ≥ 4 mm and ≥ 8 mm pocket depth using sterile paper points ISO 25,20. Samples were stored in 1.5 ml Reduced Transport Fluid media [21] at − 80 °C. All samples of one proband were pooled.

### Shotgun metagenomics sequencing analysis

DNA of all samples was extracted using the DNeasy blood and tissue kit (Qiagen, Hilden, Germany) as described earlier [22]. The procedure was performed according to the manufacturer’s protocol. Enzymatic lysis was performed with 150 µl enzymatic lysis buffer containing two enzymes, 20 µl lysozyme (20 mg/ml) and 30 µl mutanolysin (1500 U/ml; Sigma Aldrich, Taufkirchen, Germany) for 1.5 h at 37 °C to achieve efficient lysis of oral microorganisms. As a positive control for the DNA extraction and metagenomic sequencing, a mock community containing 10 species in approximately equal proportions as measured by OD_600_ was carried along, including *Fusobacterium nucleatum* (ATCC 25586), *Streptococcus mutans* (DSM 6178), *Veillonella parvula* (DSM 2008), *Parvimonas micra* (ATCC 33270), *Actinomyces odontolyticus* (DSM 19120), *Neisseria flavescens* (DSM 17633), *Streptococcus sanguinis* (DSM 20068), *Streptococcus mitis* (ATCC 11843), *Tannerella forsythia* (ATCC 43037), and *Porphyromonas gingivalis* (W381).

The extracted DNA was sent to Eurofins Genomics Germany GmbH (Ebersberg, Germany) for metagenomic sequencing, i.e. standard genomic library preparation including unique dual indexing and Illumina paired-end sequencing (Novaseq 6000 S2, 2 × 150 bp).

### Processing metagenomic data

Human contaminant reads were removed using KneadData (version 0.10.1) and a human reference database (version hg37), using the default bowtie parameters. Low-quality reads were removed or trimmed using Sickle [23] with a Phred quality score threshold of > 30 and a length threshold of 45 bp. Reads were then assembled using metaSPAdes version 3.13.0. The resulting contigs were assigned taxonomic labels by Kraken version 2 [24], and the relative abundance of species was estimated using Bracken [25]. ARGs were identified using ABRicate (version 1.0.1) and the ResFinder, NCBI, CARD, and ARG-ANNOT databases [26–29] with ARG read annotation only occurring with a sequence identity of at least 90% and coverage of at least 80%. ABRicate is only capable of detecting acquired genes and does not detect point mutations. The taxonomic assignment of ARGs was the taxonomic label of the contig in which the ARG resided, as determined by Kraken v2 using a confidence threshold of 0.05. The antibiotic that a particular ARG provides resistance to was determined using the NCBI MicroBIGG-E and CARD databases, as well as a literature search. For the comparison of genotypic and phenotypic resistance, a sample was labelled as genotypically resistant to a particular antibiotic if any ARG that provided resistance to that antibiotic was present in the sample.

### Microbial culture technique

The culture method was performed as described elsewhere [30]. In brief, serial dilutions of the thawed samples were plated onto the respective culture media, namely yeast-cysteine blood agar (HCB) and Columbia blood agar (CBA). To cultivate aerobic and facultative anaerobic bacteria, CBA plates were incubated at 36 °C and 5% CO_2_ atmosphere for 3–5 days and to cultivate anaerobic taxa, HCB plates were incubated at 36 °C for 7–10 days in an anaerobic chamber (GENbox BioMérieux, Marcy L’Etoile, France). Colony forming units (CFU) per ml were determined, and morphological colony types were visually assessed and sub-cultivated to obtain pure cultures. These were identified by applying MALDI-TOF MS (matrix-assisted desorption ionization time-of-flight mass spectrometry) in a MALDI Biotyper Microflex LT (Bruker Daltonik GmbH, Bremen, Germany). For questionable results with no species level identification, the procedure was repeated and a universal PCR, amplifying the 16S rRNA gene with the primers TP16U1 (50-AGAGTTTGATC[C/A]TGGCTCAG-30) and RT16U6 (50-ATTGTAGCACGTGTGT[A/C]GCCC-30), was performed and the amplicons were sequenced [30]. The results were analysed using the BLAST program running through NCBI (https://blast.ncbi.nlm.nih.gov/Blast) as well as through HOMD (http://www.homd.org) to identify bacterial species.

### Phenotypic antibiotic susceptibility testing

The most prevalent species in the oral biofilm samples of the majority of probands in each group were selected for phenotyping testing using the E-test. In the periodontitis group, the relevant pathogenic species were also chosen for testing. To assess phenotypic antibiotic susceptibility, one isolate (if available) of the following representative oral species was selected from each study participant’s sample: *Streptococcus oralis*, *Streptococcus anginosus*-group, *S. mutans*, *Actinomyces oris*, *Neisseria macacae*/*mucosa*, *Capnocytophaga ochracea*, *Aggregatibacter actinomycetemcomitans*, *Eikenella corrodens*, *P. gingivalis*, *Prevotella nigrescens*, *F. nucleatum*, *V. parvula*, *Lachnoanaerobaculum saburreum*, and *P. micra*. These isolates were first tested with the Kirby-Bauer disk diffusion test to screen their sensitivity to relevant antibiotic compounds: *S. oralis*, *S. anginosus*-group, *S. mutans*, and *A. oris* (gram-positive, aerobic) were tested with erythromycin (15 µg/ml), clindamycin (2 µg/ml), penicillin G (6 µg/ ml), ampicillin (2 µg/ml), vancomycin (5 µg/ml), tetracycline (30 µg/ml), gentamycin (10 µg/ml), meropenem (10 µg/ml), ciprofloxacin (5 µg/ml), cefuroxime (30 µg/ml), tigecycline (15 µg/ml), and moxifloxacin (5 µg/ml); *E. corrodens*, *A. actinomycetemcomitans*, *N. macacae*/*mucosa*, and *C. ochracea* (gram-negative, aerobic) were tested using the same antibiotics except for penicillin G, vancomycin, and moxifloxacin. For these species, azithromycin (15 µg/ml), fosfomycin (200 µg/ml), and colistin (10 µg/ml) were added. *P. gingivalis*, *P. nigrescens*, *F. nucleatum*, *V. parvula*, *L. saburreum*, and *P. micra* (anaerobic) were tested with: clindamycin (2 µg/ml), penicillin G (6 µg/ml), metronidazole (5 µg/ml), ampicillin (2 µg/ml), tetracycline (30 µg/ml), and moxifloxacin (5 µg/ml).

For the test, a suspension of a pure culture adjusted approximately to McFarland 0.5 (equivalent to approx. 10^8^ cfu/ml) was prepared. Mueller–Hinton-Blood agar plates (MBH agar plate, for aerobic/facultative anaerobic isolates) or Brucella-Broth agar plates (BBF agar plates for strictly anaerobic isolates) were inoculated either by using a rota-plater (Retro C80^™^ bioMérieux, Marcy-l’Etoile, France) for aerobic/facultative anaerobic isolates or by flooding the agar plate with the suspension followed by sucking the excessive suspension off the plate with a vacuum pump and drying it at 36 °C (anaerobic isolates). The respective disks were placed onto the agar plates and incubated for 18–72 h. The zone of growth inhibition was subsequently measured (in mm) and the isolate was interpreted according to EUCAST (European Committee on Antimicrobial Susceptibility Testing) v11.0, 2021 as susceptible (s), intermediate (i), or resistant (r).

Resistant or intermediate isolates were further tested with the Etest method (Liofilchem srl, Roseto degli Abruzzi, Italy) as previously described [31]. Colonies from pure cultures were picked and processed in the same way as for the Kirby-Bauer test (see above) and the respective Etest strips were subsequently placed on the plates. The results were interpreted using the breakpoints according to EUCAST (if available) and susceptibility was determined as susceptible (s), intermediate (i), or resistant (r). Whenever EUCAST values were not available, minimal inhibitory concentration (MIC) values for similar strains were taken from the literature and used to determine the susceptibility of the respective isolates.

### Statistical analysis

The bacterial communities were characterised by calculating diversity measures. Hill numbers when q = 0 (equivalent to species richness) and q = 1 (related to Shannon’s index) were used to measure α-diversity. To account for unequal sequencing depth, the Hill numbers were calculated from the raw read counts using rarefaction/extrapolation curves [32] and β-diversity was calculated using the Bray–Curtis dissimilarity on relative abundance data. The dominance of a sample was measured as the relative abundance of the most abundant species in the sample.

The relationship between α-diversity and group was determined using linear regression. As with all models in this study, location (Freiburg, Heidelberg, Tübingen), gender, and age were included as fixed effects while a beta regression model was used to determine the relationship between dominance and group. A PERMANOVA with 9999 permutations was performed to investigate whether bacterial composition differed between groups and also between locations. The relationship of phyla and genera relative abundance with the disease group was determined using a beta regression implemented in the package betareg (v3.1-4 [33]), with either a logit or loglog link function depending on the model fit. Furthermore, DESeq2 [34] was used to identify which species were differentially abundant between disease groups.

The resistome was characterised by the prevalence of ARGs and the difference in the number of ARGs between groups was modelled using linear regression. Firth’s logistic regression was performed to investigate which ARGs and antibiotics differed in prevalence among disease groups. The Benjamini–Hochberg procedure was used to control the false discovery rate. Furthermore, hierarchical clustering was performed on a Jaccard dissimilarity matrix for the ARG data and a Bray–Curtis dissimilarity matrix for the bacterial abundance data. Ward’s minimum variance was used for agglomeration, using the package hclust. The optimum number of clusters was determined using the gap statistic and also silhouette widths whereby the optimal number of clusters was the value of k that maximised the average silhouette width.

To compare the genotypic and phenotypic test methods to assess antibiotic resistance, both datasets were filtered to only include the samples, antibiotics, and species that were present in both datasets. The percentage of samples where both methods or just one method found resistance was subsequently calculated. All statistical analyses were performed in R v3.61.

## Results

### Clinical and epidemiological data

Table 1 shows descriptive information of the study population in relative frequencies, mean, and standard deviation (SD) for the three groups Healthy (H; n = 63, 60.3% female), Caries (C; n = 61, 31.1% female), and Periodontitis (P; n = 55, 41.8% female). In terms of their ethnicity, the subjects were predominantly of Caucasian origin. In accordance with the prevalence of the oral diseases studied here, the mean age in the H and C groups was lower (25.4 ± 6.9 and 31.5 ± 10.2) than in the P group with 54.8 ± 12.4, as expected. Concerning the study population’s level of education, a shift between the three groups was visible. The vast majority of subjects in the H group were academics (88.6%), followed by employees with completed professional training. In the C group, nearly half were academics (47.5%), 30.5% were employees with completed professional training, and 22% were employees without completed professional training. In the P group, the majority (68.6%) were subjects with completed professional training followed by academics (21.6%) According to the inclusion criteria, all participants were in good general health. With regard to possible influencing factors of the oral biofilm, the intake of antibiotics during the last 12 months was reported by 17% in the H group, 15% in the C group, and 19% in the P group. Probiotics were consumed by 5% in the H group, 7% in the C group, and 8% in the P group. The use of antiseptic mouth rinses was also low with 6% in the H group, 5% in the C group, and 17% in the P group (Table 1). The term antiseptic is used as a synonym for chlorhexidine.

### Taxonomic results—metagenomic sequencing and culture technique

**Fig. 1:**
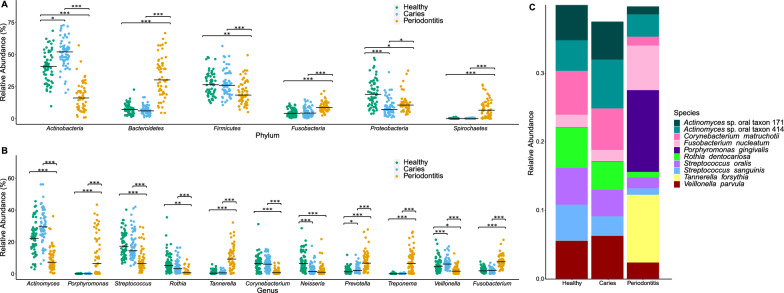
Bacterial composition of supragingival (H = Healthy, C = Caries) and subgingival (P = Periodontitis) biofilm samples of 179 study participants based on metagenomic sequencing results. **A** Significant differences in relative abundances (determined by beta regression) of the most abundant bacterial phyla in H, C, P; **B** significant differences in relative abundances of bacterial genera in H, C, P (> 5%) and **C** relative abundances of the 10 most abundant bacterial species in H, C, P. Statistical difference were evaluated using a beta regression with a logit or loglog link function depending on the model fit *< 0.05; **< 0.01; ***< 0.001. Healthy (H; n = 63, 60.3% female), Caries (C; n = 61, 31.1% female), and Periodontitis (P; n = 55, 41.8% female)

A total of 179 volunteers, comprising 63 healthy (H), 61 caries-active (C), and 55 periodontally diseased (P) participants completed the study (1 dropout). Shotgun metagenomic sequencing of oral biofilm samples resulted in 2,069,295,923 reads classified into 4856 species-level OTUs (mean number of reads 11,560,312; range: 951,395–20,823,700). As far as the artificial (mock) community used as positive control is concerned, the sequencing detected all taxa included in this community. Overall, the most abundant phyla were Actinobacteria (40.0%), Firmicutes (26.2%), Proteobacteria (14.3%), Bacteroidetes (11.5%), and Fusobacteria (5.7%) while the most abundant genera were *Actinomyces* (22.9%), *Streptococcus* (16.7%), *Veillonella* (5.5%), *Corynebacterium* (5.3%), and *Neisseria* (5.1%) (Fig. 1A, C; Additional file 2: Fig. S1A, B). Differentially abundant phyla, genera, and the top 10 most abundant species in H, C, and P are depicted in Fig. 1B, D, and E. The relative abundances of all individual taxa are depicted in Addtional file 8. The culture technique found 158 different species, with the most prevalent being *Fusobacterium nucleatum* (in 92.3% of samples), *Streptococcus oralis* (84.2%), *Actinomyces oris* (79.2%), *Veillonella parvula* (77.6%), and *Streptococcus sanguinis* (77.0%; Additional file 1: Table S1). The bacterial counts of the detected phyla, genera, and top 10 microbial species are depicted in Additional file 3: Fig. S2. Of the cultivated species, 58 were not detected by metagenomic sequencing, among them some taxa that showed high bacterial counts in the culture technique, e.g., *Selenomonas* spp. and *Parvimonas micra* (Additional file 1: Table S1, Fig. 1E, Additional file 3: Fig. S2C). In contrast, sequencing detected taxa that were not cultured (e.g., *Treponema* spp., *Escherichia coli*, *Bacteroides fragilis* etc.), and some with high abundance in the sequencing data (*Tannerella forsythia*, *Corynebacterium matruchotii*) (Fig. 1E, Additional file 3: Fig. S2C, Additional file 1: Table S6).

**Fig. 2:**
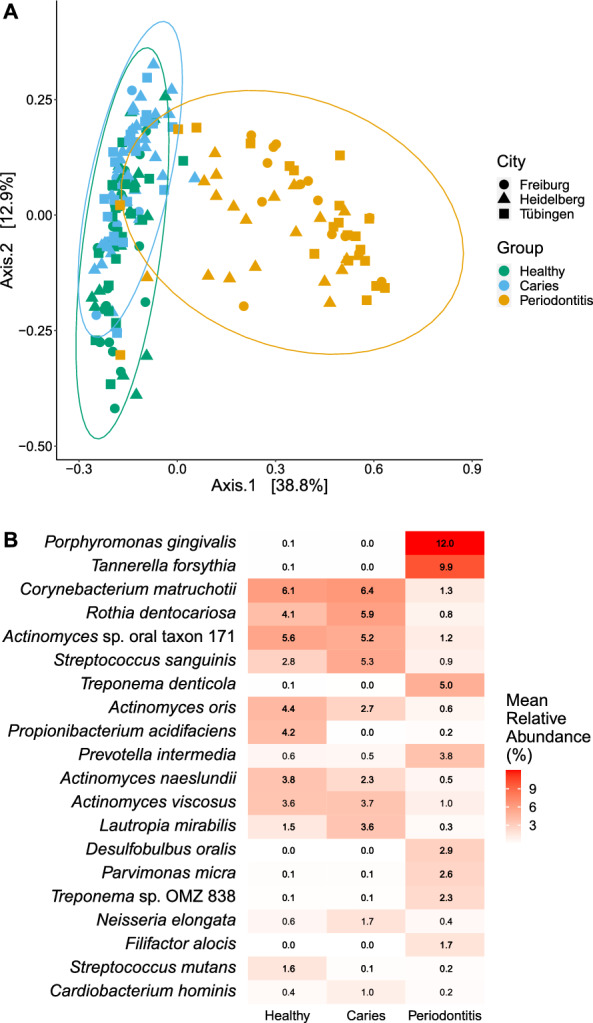
Differences within the microbiota in oral biofilm samples of 179 study participants in three different groups and three study centres based on metagenomic sequencing. **A** PCoA depicting the beta-diversity based on Bray–Curtis dissimilarity of the microbial communities in H, C, P (colours) and the three study centres (shapes; R^2^ = 0.155, p = 0.0001); **B** comparison of the mean relative abundance in H, C, P (≥ 1% in at least one group). The species shown are significantly differentially abundant between at least two groups based on DESeq2 with a log fold change > 2.5; numbers indicate the mean relative abundances. Healthy (n = 63, 60.3% female), Caries (n = 61, 31.1% female), and Periodontitis (n = 55, 41.8% female)

No α-diversity measure among the three groups reported significant differences (Additional file 4: Fig. S3). However, PERMANOVA analysis of beta-diversity revealed significant differences between the groups regarding their microbiota composition (R^2^ = 0.155, p = 0.0001) (Fig. 2A). The geographic location had no significant effect, independent of the group. In detail, analysis of differentially abundant taxa between the groups showed elevated abundances of *Porphyromonas gingivalis*, *T. forsythia*, *Treponema denticola*, *Prevotella intermedia*, *Desulfolobus oralis*, *P. micra*, *Treponema* sp. OMZ 838, and *Filifactor alocis* in P vs C and H patients. *Propionibacterium acidifaciens*, *Streptococcus mutans*, *Leptotrichia wadei*, and several *Actinomyces* spp. showed increased abundance in C vs H. *Neisseria elongata*, *Neisseria mucosa*, and *S. sanguinis* were more abundant in H than in C or P. Additionally, *C. matruchotii*, *Rothia dentocariosa*, several *Actinomyces* spp. and *Lautropia mirabilis* were less abundant in P vs C and H (Fig. 2B; Additional file 1: Table S2).

### Antibiotic resistance genes (ARGs) based on metagenomics sequencing

**Fig. 3:**
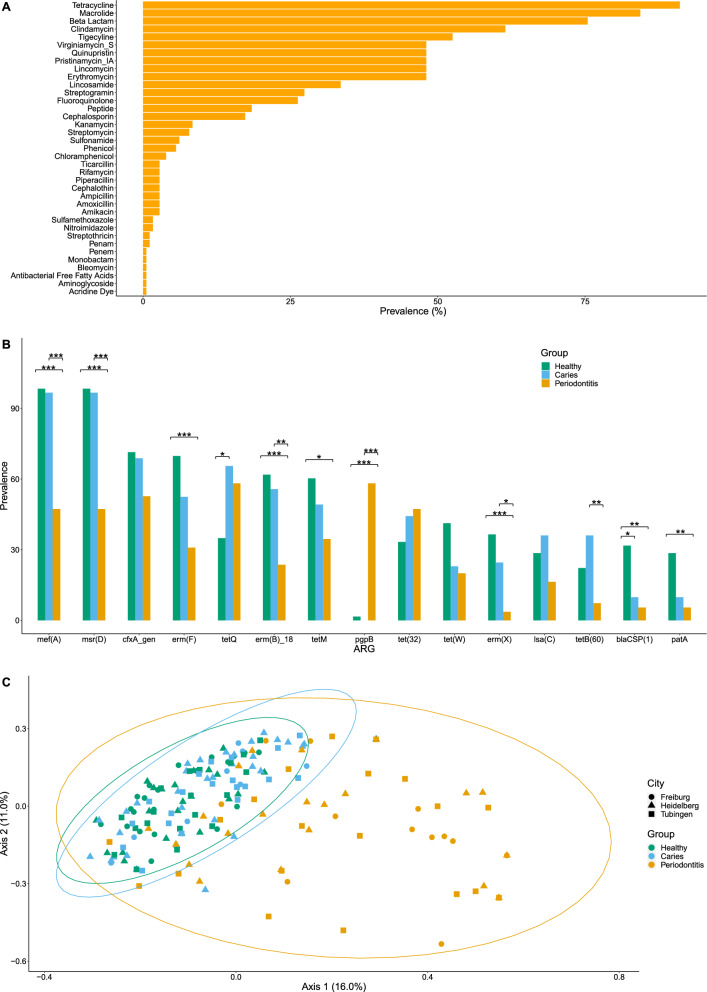
Detection of antibiotic resistance genes (ARGs) in oral biofilm samples of 179 study participants from three study centres divided into three different groups, based on metagenomic sequencing. **A** Prevalence of ARGs according to antibiotic classes and antibiotics; corresponding ARGs to specific antibiotics and antibiotic classes are listed in Additional file 1: Table S3; **B** Comparison of the 15 most prevalent ARGs in H, C, and P with significance determined by Firth’s logistic regression *p ≤ 0.05, **p ≤ 0.01 and ***p ≤ 0.001; **C** PCoA showing the ARG profiles of H, C, P (colours) and study centres (shapes); p = 0.0001; pairwise PERMANOVA: H vs C p = 0.001; H vs P p = 0.001; P vs C p = 0.001. Healthy (n = 63, 60.3% female), Caries (n = 61, 31.1% female), and Periodontitis (n = 55, 41.8% female)

ARGs were detected in all but two samples, with 64 ARGs coding for resistance to 36 antibiotics belonging to 17 antibiotic classes in total. There were significantly more ARGs in H and C than in P (p = 0.0001 and p = 0.003 resp.; Additional file 5: Fig. S4A). ARGs that were present in over 20% of samples almost always conferred resistance to tetracyclines, macrolide-lincosamide-streptogramin, or beta-lactam antibiotics. ARGs conveying resistance to aminoglycosides, fluoroquinolones, chloramphenicol, rifamycins, and different efflux pumps were less prevalent (Fig. 3A, Additional file 1: Table S3). Regarding macrolides, tetracycline, and streptomycin resistance, most samples possessed more than one ARG (94%, 76%, and 71% resp.). Overall, *msrD*, *mefA*, *cfxA,* and *ermF* were the most prevalent genes and detected in over 50% of samples (Additional file 5: Fig. S4B). The most prevalent ARGs for H and C were *mefA*, *msrD*, *cfxA*, *ermF*, *ermB*, *tetM* and also *tetQ* for C, and *pgpB*, *tetQ*, *cfxA*, *tet32*, *mefA*, and *msrD* for P (Fig. 3B). Periodontitis samples displayed a much lower prevalence of most ARGs except *pgpB*, which is the most prevalent gene in this group coding for peptide antibiotic resistance (Additional file 5: Fig. S4C). Four ARGs significantly differed in prevalence between the H and C samples: *blaCSP*(1), *tetQ*, *tetA*(46), and *tetB*(46), with the latter two genes only being found in samples from the caries group (C). A PERMANOVA analysis showed that there were significant differences between H, C, and P in their ARG profile (Fig. 3C) (R^2^ = 0.095, p = 0.0001). A Mantel test showed that samples that were more distant from each other in terms of their microbiota composition were also more distant in terms of the ARGs they share (r = 0.463, p = 0.001).

**Fig. 4:**
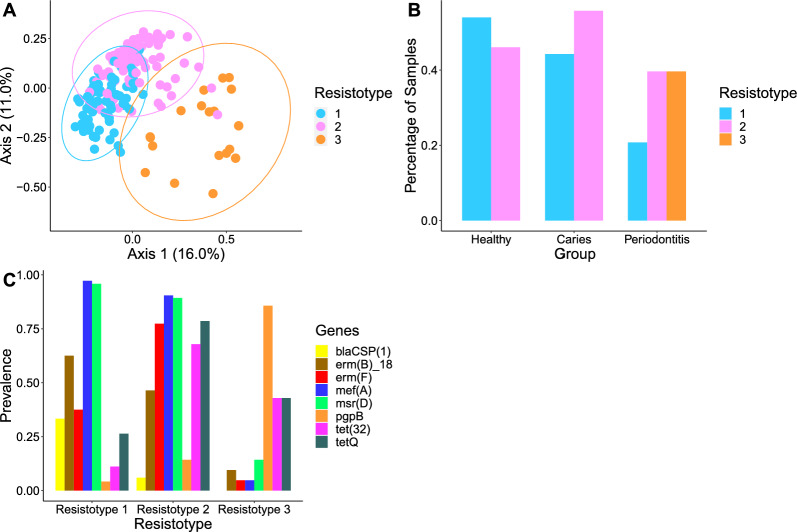
Hierarchical clustering of the detected ARGs in oral biofilm samples of three different groups of 179 study participants based on metagenomic sequencing: **A** PCoA showing three different clusters defining three resistotypes; **B** percentages of samples in H, C, and P belonging to each resistotype; **C** prevalence of eight ARGs underlying the resistotypes 1–3, with these ARGs being the most variable across the resistotypes. Healthy (n = 63, 60.3% female), Caries (n = 61, 31.1% female), and Periodontitis (n = 55, 41.8% female)

**Fig. 5:**
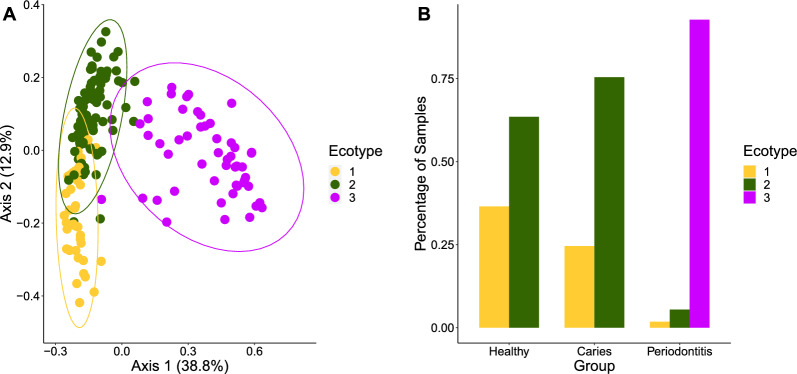
Hierarchical clustering of the bacterial taxa in oral biofilm samples of three different groups of 179 study participants based on metagenomic sequencing; **A** PCoA clustering of the microbial composition resulting in three clusters defining three ecotypes; **B** percentages of samples from H, C, and P belonging to each ecotype. Healthy (n = 63, 60.3% female), Caries (n = 61, 31.1% female), and Periodontitis (n = 55, 41.8% female)

Hierarchical clustering was used to group samples into three distinct clusters, termed resistotypes (Fig. 4A–C). Resistotype 1 was characterized by a high prevalence of *mefA*, *msrD*, *ermB*, *bla*CSP(1) and low *pgpB*, *tet32* and *tetQ*, resistotype 2 by a higher prevalence of *ermF* and *tet32*, *tetQ* and lower *ermB* and *bla*CSP(1), and resistotype 3 by a high prevalence of *pgpB* and low *mefA*, *msrD*, *ermF*, and *ermB*. Resistotype 3 was only present in periodontitis samples, whereas types 1 and 2 were present in all groups, mainly H and C (Fig. 4C). Similarly, the samples were clustered into three ecotypes based on their microbial composition (Fig. 5A, B). The bacterial composition of H and C samples overlapped greatly and was based on ecotypes 1 and 2, whereas ecotype 3 was only detected in periodontitis (mainly due to the taxa *Porphyromonas* and *Tannerella*). There was a significant association between the resistotypes and the ecotypes (Chi-squared = 83.8, p < 0.001).

### Phenotypic resistance and comparison with the resistome

The phenotypic resistance analysis using Etest found resistance to 15 of the 16 antibiotics tested, the exception being meropenem, although vancomycin resistance was only found in one sample. The metagenomic study did not find evidence of resistance to vancomycin, gentamicin, or fosfomycin, despite ARGs for these antibiotics being present in the database.

For the phenotypic testing, a selection of 997 isolates cultivated from samples from H (325 isolates from 52 individuals), C (300 isolates from 42 individuals), and P (372 isolates from 50 individuals) was tested for resistance to relevant antibiotics. The most prevalent phenotypic resistance in all three groups together based on the number of tested isolates per antibiotic was gentamicin (74.57%), azithromycin (69.48%), ciprofloxacin (52.22%), fosfomycin (45.78%), and erythromycin (39.08%) (Table 2). Regarding the different bacterial species, the largest number of isolated strains with resistances belonged to the species *S. oralis* (104), *V. parvula* (100), *A. oris* (99), *F. nucleatum* (97), *Neisseria macacae*/*mucosa* (86), and *Prevotella nigrescens* (84) (Table 3). Across all tested antibiotics, the species with the most phenotypic resistances was *N. macacae*/*mucosa* (Table 3). All results of phenotyping resistance are depicted in Additional file 9.

**Table 2:** Phenotypic testing (Etest method) for antibiotic resistance of cultured isolates from healthy participants (H), caries patients (C), and periodontitis patients (P)

	Total nr of tested isolates	% Resistant in H, C, P	% Resistant in H	% Resistant in C	% Resistant in P
Gentamicin	586	74.57	73.12	76.80	73.97
Azithromycin	249	69.48	60.71	81.82	68.69
Ciprofloxacin	586	52.22	51.61	59.67	46.58
Fosfomycin	249	45.78	44.05	45.45	47.47
Erythromycin	586	39.08	43.01	38.12	36.53
Colistin	249	36.95	35.71	33.33	40.40
Cefuroxime	586	23.21	22.58	28.18	19.63
Clindamycin	997	21.26	21.85	18.33	23.12
Metronidazole	411	19.46	22.30	21.85	15.03
Tetracycline	997	16.45	16.62	17.67	15.32
Penicillin	748	15.51	21.58	15.38	10.26
Moxifloxacin	748	14.97	14.94	18.80	11.72
Ampicillin	997	10.03	13.23	9.00	8.06
Tigecycline	586	6.83	5.91	8.29	6.39
Vancomycin	337	0.30	0.98	0.00	0.00
Meropenem	586	0.00	0.00	0.00	0.00

Numbers and percentages of isolates in resp. groups resistant to tested antibiotics are shown

**Table 3:** Phenotypic testing (Etest method) for antibiotic resistance of cultured isolates from H, C, and P

Species	Number of isolates resistant to resp. antibiotic																Total number of isolates in resp. group			
	TET	CEF	MET	CIP	ERY	AZI	CLI	PEN	FOS	MOX	AMP	GENT	TIG	COL	VAN	MER	All	H	C	P
*S. oralis*	28	6	nd	104	37	nd	9	0	nd	0	3	104	0	nd	0	0	104	38	31	35
*V. parvula*	11	nd	70	nd	nd	nd	0	64	nd	24	43	nd	nd	nd	nd	nd	100	37	29	34
*A. oris*	0	0	nd	89	1	nd	3	0	nd	5	0	14	0	nd	0	0	99	35	29	35
*F. nucleatum*	0	nd	3	nd	nd	nd	1	15	nd	2	16	nd	nd	nd	nd	nd	97	40	27	30
*N. macacae/mucosa*	30	66	nd	7	86	86	85	nd	3	nd	2	50	22	1	nd	0	86	30	25	31
*P. nigrescens*	22	nd	3	nd	nd	nd	14	31	nd	17	30	nd	nd	nd	nd	nd	84	25	29	30
*C. ochraceae*	1	19	nd	6	22	34	3	nd	66	nd	0	78	1	80	nd	0	81	31	19	31
*E. corrodens*	12	44	nd	1	63	53	75	nd	29	nd	3	56	17	11	nd	0	75	23	22	30
*S. anginosus*	9	1	nd	42	12	nd	4	0	nd	0	0	61	0	nd	0	0	61	10	18	33
*P. micra*	0	nd	2	nd	nd	nd	9	2	nd	6	3	nd	nd	nd	nd	nd	59	16	8	35
*L. saburreum*	44	nd	2	nd	nd	nd	0	2	nd	57	0	nd	nd	nd	nd	nd	57	21	26	10
*S. mutans*	0	0	nd	39	0	nd	0	0	nd	0	0	38	0	nd	0	0	41	1	30	10
*S. intermedius*	4	0	nd	12	5	nd	2	0	nd	0	0	20	0	nd	0	0	20	16	4	nd
*P. gingivalis*	0	nd	0	nd	nd	nd	4	0	nd	1	0	nd	nd	nd	nd	nd	14	0	0	14
*S. constellatus*	3	0	nd	3	2	nd	1	1	nd	0	0	8	0	nd	1	0	8	2	3	3
A. actino^*^	0	0	nd	0	1	0	2	nd	1	nd	0	4	0	0	nd	0	7	0	0	7
*S. gordonii*	0	0	nd	1	0	nd	0	0	nd	0	0	2	0	nd	0	0	2	0	0	2
*S. infantis*	0	0	nd	1	0	nd	0	0	nd	0	0	1	0	nd	0	0	1	0	0	1
*S. mitis*	0	0	nd	1	0	nd	0	0	nd	0	0	1	0	nd	0	0	1	0	0	1

Resistant isolates of resp. bacterial species, total number of isolates, and numbers of isolates in H, C, and P

*TET* tetracycline, *CEF* cefuroxime, *MET* metronidazole, *CIP* ciprofloxacin, *ERY* erythromycin, *AZI* azithromycin, *CLI* clindamycin, *PEN* penicillin, *FOS* fosfomycin, *MOX* moxifloxacin, *AMP* ampicillin, *GEN* gentamycin, *TIG* tigecycline, *COL* colistin, *VAN* vancomycin, *MER* meropenem

^*^A. actino Aggregatibacter actinomycetemcomitans

^**^nd not determined, meaning testing this antibiotic was not appropriate or relevant for the resp. species due to its metabolism or other characteristics

**Fig. 6:**
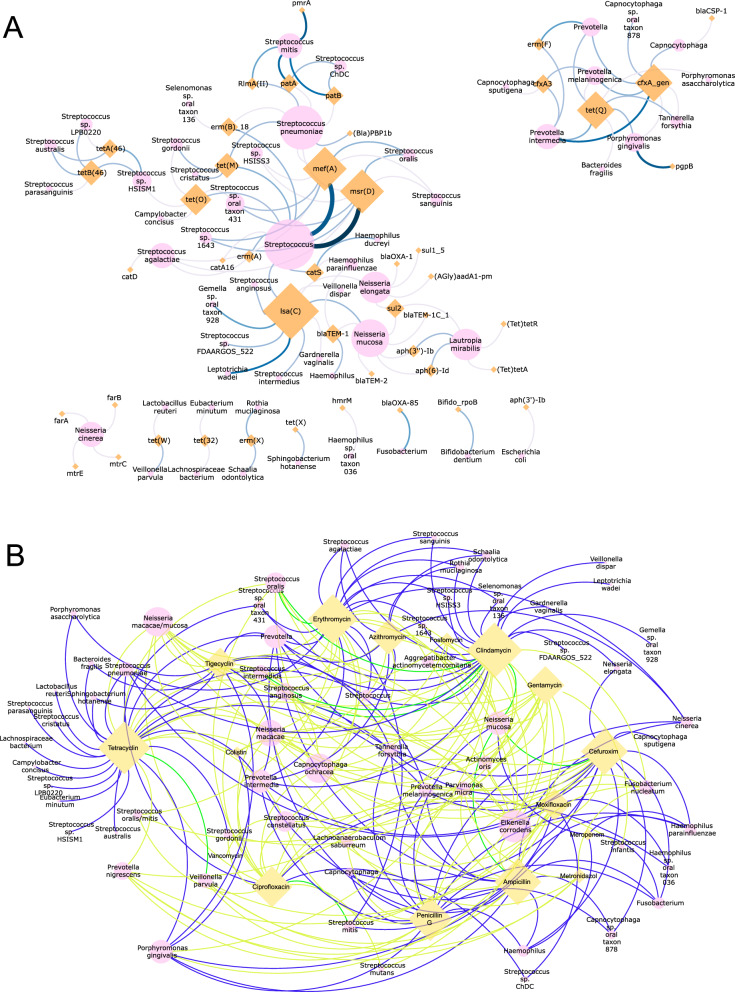
Network analysis showing associations between antibiotic resistance and bacterial taxa in oral biofilm samples of 179 study participants. **A** The associations between ARGs and species/genera that were found through metagenomic sequencing. Each connection means that the resp. ARG was associated with the resp. taxon. The colour and width of the edges represent the number of samples in which the association between ARG and taxa was found. Only associations with taxa on the genus or species level are shown. **B** Associations between antibiotic resistance and bacterial species that were found from both the phenotypic testing and the metagenomic sequencing. The yellow edges indicate that association was only found in the phenotypic tests, the blue edges represent associations that were only found by sequencing, while the green edges are associations that were found by both methods

In total, 48 different species were found to possess ARGs according to metagenomic sequencing (Additional file 1: Table S4). The species that most commonly possessed ARGs were *Streptococcus mitis* (27.1% of samples the species was present in), *P. gingivalis* (20.9%), *P. intermedia* (15.8%), *L. wadei* (11.3%), and *Gemella* oral taxon 928 (6.2%). The species *A. oris, Capnocytophaga ochracea, F. nucleatum*, *Eikenella corrodens, P. micra*, *S. mutans, Aggregatibacter actinomycetemcomitans*, and *Streptococcus constellatus* were shown to be resistant to several antibiotics in phenotypic tests but did not display resistance to any of the tested antibiotics according to sequencing (Additional file 1: Tables S4 and S5). The disparity is particularly pronounced for *E. corrodens* and *C. ochracea*, which demonstrated phenotypic resistance to 11 and 10 of the 16 tested antibiotics respectively. The comparison between the metagenomic and phenotypic data is not trivial since these data cannot be compared directly. To address this challenge, one approach was to link the resistance gene data with the associated species carrying the gene using bioinformatic tools, whereby most of the associations of ARGs with species (89%) concerned species that were not tested phenotypically (Additional file 1: Tables S4, S5; Additional file 6: Fig. S5, Fig. 6B).

The network analysis in Fig. 6A shows the associations between the ARGs and the microbial genera and species that were found with metagenomic sequencing. High percentages of associations were found for several streptococcal species with ARGs for beta-lactam, fluoroquinolone, macrolide, and chloramphenicol resistance, and several *Neisseria* species with ARGs for beta-lactam, macrolide, and aminoglycoside resistance as well as efflux pumps and *P. gingivalis* with pgpB. Further associations between particular antibiotics and bacterial species considering the phenotypic and genotypic resistance analysis are depicted in Fig. 6B, Additional file 7: Fig. S6, and Additional file 1: Table S4. Although numerous associations were found between specific antibiotic resistance and bacterial taxa, only a few taxa showed matching associations both with genotypic and phenotypic analysis, e.g., *S. oralis* with erythromycin and clindamycin resistance, *S. mitis* with ciprofloxacin resistance, *N. mucosa* with cefuroxime, ampicillin, and clindamycin resistance, and *V. parvula* with tetracycline resistance.

**Fig. 7:**
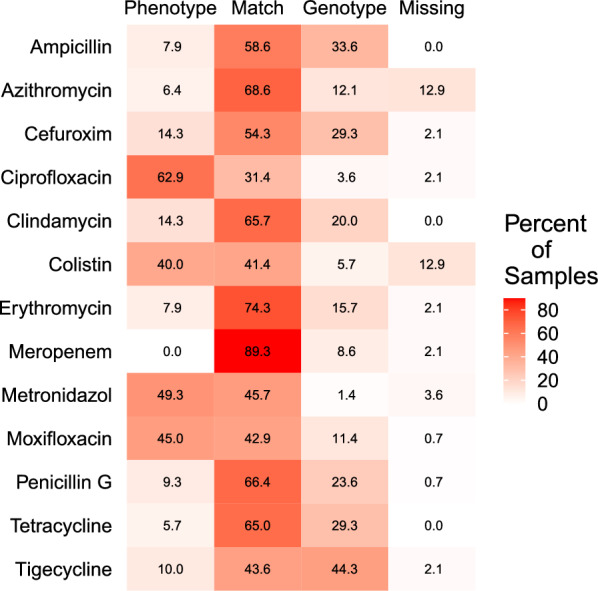
Comparison of phenotypic resistance (of 997 selected isolates and determined with Etest) and genotypic resistance (based on the detection of ARGs by metagenomic sequencing) in oral biofilm samples of 179 study participants. The heatmap shows percentages of matches of positive and negative results in the samples regarding a set of 13 antibiotics. Phenotype: phenotypic resistance but no genotypic resistance; Genotype: genotypic resistance but no phenotypic resistance; Match: both methods in agreement (resistance or no resistance); Missing: no phenotypic test.

However, when the associations with specific taxa were disregarded and only those antibiotics that were tested phenotypically and for which genotypic results were found were considered, there was a 59% level of agreement between the two methods. Here, the data from the selected species for the culture and E-Tests was taken for the comparison of both approaches. For most antibiotics, there was an agreement greater than 60% between the methods, with the discrepancy usually being due to higher predicted resistance from the metagenomic data than phenotypically tested resistance (Fig. 7). The exceptions were ciprofloxacin, colistin, metronidazole, and moxifloxacin where the prevalence of resistance was higher in phenotypic tests. As discussed earlier, this can be due to a bias introduced by the DNA extraction methods and, possibly, a lower sensitivity of the metagenomic sequencing for certain species, which highlights the need for using more than one method to comprehensively assess resistance in the oral microbiota. Additionally, genes encoded in plasmids or mobile elements are not associated with any species and therefore induce a bias when reconstructing the link between species and ARG detection (Additional file 8).

## Discussion

Like other human commensals, the oral microbiota has been hypothesized to be a reservoir for microbial resistance genes. To investigate this, we sampled oral biofilm from 179 study participants from healthy (H), caries (C), and periodontally diseased individuals (P). The samples were analysed using shotgun metagenomic sequencing combined with culture technique resulting in 4856 species-level OTUs and 158 cultured bacterial species. This study is the first to investigate antimicrobial resistance by assessing both ARGs by sequencing and phenotypic resistance from selected cultured isolates. We conclude that the oral microbiome is a rich reservoir for multiple ARGs which can be clustered into different resistotypes based on the specific differences in the microbiota composition and also depending on the respective condition of the individuals, i.e. health, caries, or periodontitis. Notably, the prevalence of ARGs was highest in H, and significantly higher in H and C than in P patients (Additional file 9).

Overall, the genera *Actinomyces*, *Streptococcus*, *Veillonella*, *Corynebacterium*, *Neisseria*, and *Fusobacterium* dominated in H, which is reflected for the most part in both the sequencing and the culture technique. This core oral microbiome has been confirmed in numerous studies using PCR cloning methods and amplicon sequencing of the 16S rRNA genes [9, 13, 35–37]. Compared with amplicon sequencing studies, we found a higher abundance of the genus *Actinomyces* (and the phylum Actinobacteria) than *Streptococcus* (and the phylum Firmicutes) which might be due to methodological differences in the direct shotgun sequencing approach. In comparison with H, in C the genera *Veillonella* and *Prevotella* were significantly more abundant (Fig. 1D). Additionally, the species *P. acidifaciens*, *S. mutans*, and different *Actinomyces* species showed significantly higher abundances in C than in H or P (Fig. 2B). This result confirms molecular studies that revealed not only *S. mutans* but a polymicrobial community representing all these taxa associated with cariogenic plaque [9, 38–41]. In P, the genera *Porphyromonas*, *Tannerella*, *Prevotella*, *Treponema*, and *Fusobacterium* showed a significantly higher abundance than in H and C (Fig. 1D). The species *P. gingivalis*, *Desulfobulbus oralis*, *T. forsythia*, *T. denticola*, *F. alocis*, *P. micra*, and *P. intermedia* showed log fold changes ≥ 2 to 9 or significantly higher abundances in P versus H and C, resp. (Additional file 1: Table S2, Fig. 2B). All these taxa were shown to be characteristic of periodontitis in molecular studies [13, 42–44]. The only taxa that stand out as significantly more abundant in H were the phylum Proteobacteria and the genus *Neisseria*. Belda-Ferre et al. accordingly found *Neisseria* to be highly abundant in healthy individuals [45] and although *Neisseria* spp. were reported to be enriched in enamel caries [46], it was found to be decreased in progressive caries [47], and thus these previous results and the results from our study indicate that the relative abundance of genus *Neisseria* could be health-associated.

Consequently, significant differences were revealed in the beta-diversity of the three groups (Fig. 2A), although there were overlaps between H and C when hierarchical clustering was performed leading to three ecotypes (Fig. 5). Presumably, differential abundances between C and H were less pronounced since we sampled supragingival biofilm, and not directly within carious lesions. Altogether, we recruited a very representative study population portraying typical taxonomic compositions for the respective oral conditions of health, caries, and periodontitis. To date, shotgun analysis has only scarcely been able to analyze the subgingival microbiota correlated with healthy and periodontitis subjects. The difference between the microbiome sequencing methods used may explain the results presented in our study. Nevertheless, in their very recent and extensive review report about the microbial diversity of periodontitis, Balan et al. [48] described contradictory results in the literature. While most studies reported an increasing diversity of the microbial taxa associated with periodontitis, some other studies reported the opposite.

The metagenomic sequencing provided a comprehensive analysis and revealed a high prevalence of resistance genes in all three groups. ARGs with a prevalence of over 20% mostly conferred resistance to antibiotics targeting microbial protein biosynthesis and cell wall synthesis. The spectrum of ARGs that we found included resistance to macrolides, fluoroquinolones, and ampicillin, all of which are classified by the WHO as critically important antimicrobials for human medicine [49]. The range we found resembles the results of Carr et al., who studied dental biofilm samples from China and the US in that, mostly resistance to tetracyclines, macrolide-lincosamide streptogramin antibiotics, and beta-lactams was detected [14]. In contrast in our study, the prevalence of fluoroquinolone was lower and we did not find genotypic resistance to glycylglycine or pleuromutilin [14]. These differences could be due to the variations in the samples, local antibiotic usage, or over-the-counter availability in different countries.

The ARGs with the highest prevalence in H and G were *mefA*, *msrD*, *cfxA*, and *ermF* and *tetQ*, *pgpB*, and *tet32* in P. Our results corroborate the findings of Caselli et al. [50] who analysed the oral resistome of healthy Italians with a metagenomics approach for taxonomy and a microarray for ARG detection. They also report the presence of over 60 ARGs, with the highest prevalence for macrolide-lincosamide-streptogramin and tetracycline resistance, although they detected a lower prevalence of ARGs for beta-lactam antibiotics. While earlier studies assessing ARGs in oral samples revealed genes for tetracycline and macrolide-lincosamide-streptogramin resistance and some beta-lactam resistance genes, most studies did not use open-ended sequencing methods and only provided data for relatively small study populations [51–55]. Functional metagenomic approaches mostly detected tetracycline resistance genes, most frequently *tetM*, which we found in our study with a prevalence of 30–60% and erythromycin genes, albeit less frequently [6, 11, 53, 56, 57]. Particularly tetracycline resistance genes as well as erythromycin genes and *mef* genes have been found on conjugative elements, e.g., Tn916, predestining them for dissemination through horizontal gene transfer [6, 11].

In P, *pgpB* was the most prevalent ARG not detected in the other groups. Other authors, using specific PCRs, have not reported *pgpB* in periodontitis, but frequently found *tetQ*, *tetM*, *cfxA*, and *bla*_TEM_ genes [51, 54, 55, 58]. *PgpB* is a chromosomally encoded gene in *P. gingivalis*, which we found in high abundances in P.

Interestingly, significantly fewer ARGs were detected in P than in H and C, which can be explained by the lower diversity and differing microbial composition in periodontitis. This is confirmed by the resistotypes the ARGs were clustered in, which were associated with the underlying microbiota composition. Resistotypes 1 and 2 were present in all three groups, mostly in H and C, which also showed overlapping ecotypes, whereas resistotype 3 was only present in P and was dominated by the pgpB gene and tetracycline resistance genes (Fig. 4).

In this study, we investigated the microbiota corresponding to the specific health/disease condition. Since the subgingival biofilm is the etiological agent for periodontitis, we investigated the subgingival niche, whereas for the caries and healthy groups, the main focus was on the supragingival biofilm. This could be the reason for some of the discrepancies in the frequency of the ARGs revealed in the different microbial niches.

In contrast to other studies, we expanded the genotypic results of metagenomic sequencing by performing phenotypic resistance tests on nearly 1000 isolates. The direct comparison of both methods is limited by the fact that for the Etests a subset of 14 species and 12 relevant antibiotics had to be selected. Also, bacteria might be equipped with an ARG which is not expressed and would not result in phenotypic resistance and a high percentage of oral taxa is as yet uncultivated [59] and might not be captured with the culture technique. However, despite these restrictions, a surprisingly high agreement of almost 60% was found between the two methods regarding the presence and absence of antibiotic resistance in the samples tested with both methods. Similar to the genotypic results, phenotypic resistance to macrolides-lincosamides (azithromycin, erythromycin, clindamycin), fluoroquinolones (ciprofloxacin), and beta-lactams (cefuroxime and penicillin) was also highly prevalent. Tetracycline resistance was found to be the most prevalent resistance with genotypic analysis but not with phenotypic resistance. This could be due to less phenotypic expression of this resistance gene or to the selected isolates for the phenotypic testing (Fig. 3, Table 2). Furthermore, the genotypic analysis did not find evidence for resistance to vancomycin, gentamicin, or fosfomycin, which contrasts with the phenotypic analysis, and could be because the bioinformatic methods used were not capable of detecting point mutations. In elucidating the reasons behind the mismatch between the results of the genotypic and phenotypic analysis, several technical points should be highlighted. Firstly, the DNA extraction from the biofilm samples could have led to the absence of some taxa, as the cell wall structure is different among the diverse bacterial species found in oral biofilms. An additional important point is the effect of low gene abundance on the results of shotgun analysis, as no PCR was performed prior to sequencing. Furthermore, in this study, we focused on the acquisition of antimicrobial resistance genes and did not examine mutation-induced resistance. The association between mutation-induced resistance and metagenomic data is challenging because it would require the reconstruction of the genomes or an examination of the nucleotide polymorphisms (SNP) distribution on the target genes from every species.

Usually, next-generation sequencing techniques are expected to reveal bacteria that are not detectable by culture technique. However, also in earlier studies in combination with the culture technique, not all cultured bacteria were detected using sequencing [60].

For the metagenomic sequencing used in this study, no amplification was performed before sequencing, and thus the sensitivity for detecting low-abundant species could be reduced. On the other hand, with suitable media, low-abundant species that grow rapidly can also be cultivated.

Previous studies assessing phenotypic resistance often only considered periodontitis patients [61–64]. In agreement with these studies, we found phenotypic resistance to tetracycline, metronidazole, and clindamycin as well as penicillin and ampicillin. Notably, we found a comparatively high percentage of isolates resistant to the reserve antibiotic colistin and some resistance to tigecycline, but no noteworthy resistance to vancomycin or meropenem. It is also striking that *Neisseria macacae/mucosa* and *S. oralis* isolates overall showed the most prevalent phenotypic resistance, corresponding to the finding that these genera are most abundant in H where the highest numbers of ARGs were found.

Linking the ARGs to the bacterial taxa in the network analysis revealed that mostly non-mutans streptococci, e.g., *S. mitis*, *Streptococcus pneumoniae*, *Streptococcus australis*, and *Streptococcus agalactiae* harboured diverse ARGs for tetracycline, macrolide, fluoroquinolone, and chloramphenicol resistance. Other commensals, *Neisseria* spp., *Haemophilus* spp., and *Fusobacterium* spp. were associated with beta-lactam and tetracycline resistance genes and genes for efflux pumps. For several *Streptococcus* taxa, it was shown that clindamycin, erythromycin, and ciprofloxacin resistance was genotypically present and phenotypically expressed and also for *N. mucosa* and *V. parvula*, several genotypic and phenotypic resistances matched. It should be emphasized that it is challenging to directly compare the results of the phenotypic E-Tests with the genotypic tests since a bias is introduced by the choice of the 14 most prevalent oral species that we used for the phenotypic analysis. While we successfully analyzed nearly 1000 isolates from these species, the analysis of additional samples was not feasible in the context of this research. Hence, the differences between the results derived from both techniques could not be avoided due to the different proportions of the various species that were investigated. For instance, fosfomycin resistance occurs in *Capnocytophaga ochracea* which we only detected at a low level of abundance in our sequencing data, thereby most likely leading to incomplete coverage of the genome.

For the gentamicin resistance, *S. oralis* is potentially resistant due to the expression of the AAC(6ʺ)-APH(2″) genes but since these genes are mostly encoded on plasmids the resistance cannot be attributed to a specific species when the results of the metagenomic sequencing are examined. Furthermore, due to the focus and objectives of our research, the applied sequencing method does not investigate mutations but rather analyzes genes leading to resistance.

Although we focused on the biofilm niches corresponding to the health status (caries, periodontitis, and health), it would also have added value to the investigation if similar biofilm samples (subgingival biofilm samples in caries and healthy groups, and supragingival samples in the periodontitis-group) would also have been collected and analyzed.

In conclusion, we found the oral microbiota to harbor a diverse array of resistance genes and display a high prevalence of phenotypically expressed resistance. Furthermore, a method combination is required to reveal the antibiotic resistance potential in oral biofilm, as both shotgun analysis and phenotypic testing yielded different results. Clustering of the ARGs according to the oral condition (H, C, or P) was found, with H being the richest in ARGs. The specific framework conditions within the oral biofilm increase the possibility for bacteria to exchange ARGs located on mobile genetic elements [65–67]. Hence, dissemination of resistance is a possibility since oral bacteria can reach other body sites and their transfer to the gut microbiome has been shown to be more extensive than previously assumed [15]. In light of these data, and the evidence that antibiotics often are prescribed in dentistry without indication [68–70], prudent use of antibiotics is highly recommended and further research needs to shed light on potential horizontal gene transfer and dissemination of resistance through oral bacteria. From a clinical point of view, a prudent approach to antibiotic use in dentistry is recommended.

## Supplementary Information

**Additional file 1: Table S1.** Bacterial species detected in oral biofilm samples of 179 study participants with culture technique. Some of the taxa could not be unambiguously identified to species level, thus the possible species identifications are listed, some could only be identified to genus level. Species in bold were only found with culture technique and not by sequencing. **Table S2.** Log fold change of bacterial species abundance found in oral biofilm samples of 179 study participants with metagenomics sequencing. Comparison of the three study groups, healthy, cariesand periodontitis. **Table S3.** ARGs found in oral biofilm samples of 179 study participantswith metagenomics sequencing and corresponding antibiotics they confer resistance to. **Table S4.** Bacterial species that were assigned to the ARGs found in oral biofilm samples of 179 study participants with metagenomics sequencing. Only assignments that could be made to the species level are included in the table. **Table S5.** Bacterial speciesassociated with resistance to the phenotypically-tested antibiotics in both the phenotypicand metagenomic sequencing methods. Red: phenotypic resistance; blue: genotypic resistance; yellow: both methods found resistance. The species highlighted in grey are those that were tested in the phenotypic study. **Table S6.** The mean relative abundancesof species found through metagenomic sequencing. The mean relative abundance in healthy, caries and periodontitis samples are reported separately, along with the overall mean across all groups. Only the species with a mean overall abundance >0.1% are listed.

**Additional file 2: Figure S1.** Bacterial composition of supragingivaland subgingivalbiofilm samples of 179 study participants based on metagenomic sequencing results. The 12 most abundant generaare shown A) Mean relative abundances in percent in three age categories; B) Mean relative abundances in percent in males and females. Healthy, Caries, and Periodontitis.

**Additional file 3: Figure S2.** Bacterial composition of supragingivaland subgingivalbiofilm samples of 179 study participants based on culture technique as bacterial count in % CFU. A) Bacterial count of microbial phyla in H,C,P; B) Bacterial count of microbial genera in H,C,P; C) Bacterial count of 10 most abundant microbial species in H,C,P. Healthy, Caries, and Periodontitis.

**Additional file 4: Figure S3.** Diversity measures of the microbial composition in biofilm samples of 179 study participants based on metagenomic sequencing. The differences in A) Species richness, B) Alpha diversity; and C) dominance between the three groups with different oral conditions. Healthy, Caries, and Periodontitis.

**Additional file 5: Figure S4.** Detection of antibiotic resistance genesin oral biofilm samples of three different groupsof 179 study participants based on metagenomic sequencing. A) Numbers of ARGs present in samples from H,C,P; B) Prevalence of the ARGs across all samples; C) Prevalence of resistance to the different antibiotics/ antibiotic classes in H,C,P with significance determined using Firth’s logistic regression. * <0.05; **<0.01; ***<0.001. Healthy, Caries, and Periodontitis.

**Additional file 6: Figure S5.** Bacterial taxa underlying the 3 ecotypesfound in oral biofilm samples of three different groupsof 179 study participants based on metagenomic sequencing. Healthy, Caries, and Periodontitis.

**Additional file 7: Figure S6.** Comparison of phenotypic resistanceand genotypic resistancein oral biofilm samples of 179 study participants. The agreement between the phenotypic and sequencing methods in terms of whether each of the species tested phenotypically provides resistance to each of the tested antibiotics. Phenotype Resistance: only phenotypic resistance found; Match: percentage of samples where both methods agreed on resistance; Genotype Resistance: only genotypic resistance found; Phenotype Missing: species was not tested phenotypically; Genotype Missing: species was not found with sequencing; Missing in both: species resp. resistance was not found with either method.

**Additional file 8:** Metagenomics sequencing results with taxonomic assignment.

**Additional file 9:** Raw data of the phenotypic resistance analysis.

## Data Availability

Sequence data are available at GenBank under BioProject: PRJNA817430 (accession numbers will be released before publication): https://dataview.ncbi.nlm.nih.gov/object/PRJNA817430?reviewer=kh1852h73oqpv91f9ll4oe67iv. All other data are contained within the main manuscript and Additional files.

## Code availability

The following software packages were used: KneadData (v0.10.1), available at https://github.com/biobakery/biobakery/wiki/kneaddata, Sickle (v1.33), available at https://github.com/najoshi/sickle, metaSPAdes (v3.13.0), available at https://github.com/ablab/spades, Kraken2 (v2.1.2), available at https://github.com/DerrickWood/kraken2, Bracken (v2.5), available at https://github.com/jenniferlu717/Bracken, ABRicate (v1.0.1), available at https://github.com/tseemann/abricate, R (v4.1.2), available at https://cran.r-project.org/bin/windows/base/. The following R packages were used: betareg (v3.1-4), cluster (v2.1.2), DESeq2 (v1.34.0), emmeans (v1.7.0), ggplot2 (v3.3.5), iNEXT (v2.0.20), lme4 (v1.1-27.1), logistf (v1.24), microbiome (v1.16.0), and phyloseq (v1.38.0), which are available at https://cran.r-project.org/web/packages/.
