# Supplementary material for: The oral microbiota is a reservoir for antimicrobial resistance: resistome and phenotypic resistance characteristics of oral biofilm in health, caries, and periodontitis
Source: Ann Clin Microbiol Antimicrob. 2023 May 13;22:37. doi: 10.1186/s12941-023-00585-z (PMC10183135; doi:10.1186/s12941-023-00585-z)
Supplement: Supplementary file 1 — Additional file 1: Table S1. Bacterial species detected in oral biofilm samples of 179 study participants with culture technique. Some of the taxa could not be unambiguously identified to species level, thus the possible species identifications are listed, some could only be identified to genus level. Species in bold were only found with culture technique and not by sequencing. Table S2. Log fold change of bacterial species abundance found in oral biofilm samples of 179 study participants with metagenomics sequencing. Comparison of the three study groups, healthy, cariesand periodontitis. Table S3. ARGs found in oral biofilm samples of 179 study participantswith metagenomics sequencing and corresponding antibiotics they confer resistance to. Table S4. Bacterial species that were assigned to the ARGs found in oral biofilm samples of 179 study participants with metagenomics sequencing. Only assignments that could be made to the species level are included in the table. Table S5. Bacterial speciesassociated with resistance to the phenotypically-tested antibiotics in both the phenotypicand metagenomic sequencing methods. Red: phenotypic resistance; blue: genotypic resistance; yellow: both methods found resistance. The species highlighted in grey are those that were tested in the phenotypic study. Table S6. The mean relative abundancesof species found through metagenomic sequencing. The mean relative abundance in healthy, caries and periodontitis samples are reported separately, along with the overall mean across all groups. Only the species with a mean overall abundance >0.1% are listed. [file 12941_2023_585_MOESM1_ESM.docx]

**Additional file: Tables**

The oral microbiota is a reservoir for antimicrobial resistance: Resistome and phenotypic resistance characteristics of oral biofilm in health, caries and periodontitis

Anderson AC, von Ohle C, Frese C, Boutin S, Bridson C, Schoilew K, Peikert SA, Hellwig E, Pelz K, Wittmer A, Wolff D, Al-Ahmad A

Additional file Results regarding diversity measures based on the metagenomics sequencing data - the effect of city and gender

There was a significant interaction between disease group and city for α-diversity and dominance of the microbiome, due to periodontitis samples having lower α-diversity (F=6.83, p<0.001) and higher dominance (F=4.03, p=0.003) than other disease groups in Tübingen, but with no difference in other cities. Similarly there was a significant interaction between disease group and city for β-diversity (R2=0.030, p=0.007). There were also differences in relative abundance of Spirochaetes (F=5.59, p=0.015), Fusobacteria (F=4.88, p=0.015) and Firmicutes (F=5.03, p=0.015) among cities, and also females had higher levels of Actinobacteria (F=9.56, p=0.011) and Proteobacteria (F=6.94, p=0.025) than males. There was also a significant interaction between disease group and city in the number of ARGs in a sample, as periodontitis samples do not possess fewer ARGs in Heidelberg (F=4.30, p=0.003).

**Table S1**: Bacterial species detected in oral biofilm samples of 179 study participants with culture technique. Some of the taxa could not be unambiguously identified to species level, thus the possible species identifications are listed, some could only be identified to genus level. Species in bold were only found with culture technique and not by sequencing.

| ***Abiotrophia defectiva*** | *Fusobacterium periodonticum* | *Porphyromonas/Prevotella/*  *Alloprevotella* spp. |
| --- | --- | --- |
| *Actinobaculum* spp*.* | *Fusobacterium* spp. | ***Prevotella buccae*** |
| ***Actinomyces georgiae*** | *Gemella haemolysans* | *Prevotella denticola* |
| ***Actinomyces gerencseriae*** | *Gemella morbillorum* | ***Prevotella histicola*** |
| *Actinomyces israelii* | ***Gemella sanguinis*** | *Prevotella intermedia* |
| *Actinomyces naeslundii* | *Gemella spp.* | ***Prevotella loescheii*** |
| ***Actinomyces odontolyticus*** | ***Granulicatella adiacens*** | ***Prevotella maculosa*** |
| *Actinomyces oris* | *Haemophilus* spp*.* | *Prevotella melaninogenica* |
| *Actinomyces* spp*.* | *Haemophilus haemolyticus* | ***Prevotella nigrescens*** |
| *Aggregatibacter actinomycetemcomitans* | *Haemophilus parainfluenzae* | ***Prevotella oralis/veroralis*** |
| *Aggregatibacter aphrophilus* | ***Johnsonella ignava*** | *Prevotella oris* |
| *Aggregatibacter segnis* | ***Kingella denitrificans*** | ***Prevotella oulorum*** |
| *Aggregatibacter spp.* | ***Kingella oralis*** | *Prevotella salivae* |
| ***Alloprevotella tannerae*** | ***Lachnoanaerobaculum orale*** | *Prevotella/Bacteroides* spp*.* |
| ***Anaeroglobus geminatus*** | ***Lachnoanaerobaculum saburreum*** | *Propionibacterium acidifaciens* |
| *Atopobium parvulum* | *Lachnoanaerobaculum* spp*.* | ***Propionibacterium acnes*** |
| ***Atopobium rimae*** | *Lachnoanaerobaculum umaense* | *Propionibacterium/ Pseudopropionibacterium* spp. |
| ***Atopobium spp.*** | *Lachnospiraceae bacterium* | ***Pseudoramibacter alactolyticus*** |
| *Bifidobacterium dentium* | *Lactobacillus fermentum* | *Rothia aeria* |
| *Bifidobacterium* spp*.* | *Lactobacillus gasseri* | *Rothia dentocariosa* |
| ***Bulleidia extructa*** | *Lactobacillus paracasei* | *Rothia mucilaginosa* |
| *Campylobacter concisus* | *Lactobacillus rhamnosus* | *Scardovia* spp. |
| *Campylobacter gracilis* | *Lactobacillus salivarius* | ***Scardovia wiggsiae*** |
| ***Campylobacter rectus*** | *Lactobacillus spp.* | ***Selenomonas artemidis*** |
| *Campylobacter showae* | ***Lactobacillus vaginalis*** | ***Selenomonas flueggei*** |
| *Capnocytophaga gingivalis* | *Lautropia mirabilis* | ***Selenomonas infelix*** |
| ***Capnocytophaga granulosa*** | *Leptotrichia buccalis* | ***Selenomonas noxia*** |
| *Capnocytophaga haemolytica* | *Leptotrichia goodfellowii* | *Selenomonas* spp. |
| *Capnocytophaga leadbetteri* | *Leptotrichia hofstadii* | *Selenomonas sputigena* |
| *Capnocytophaga ochracea* | *Leptotrichia shahii* | ***Slackia exigua*** |
| *Capnocytophaga spp.* | *Leptotrichia* spp*.* | ***Solobacterium moorei*** |
| *Capnocytophaga sputigena* | ***Leptotrichia trevasanii*** | *Staphylococcus aureus* |
| *Cardiobacterium hominis* | *Leptotrichia wadei* | *Streptococcus anginosus group* |
| ***Cardiobacterium* spp**. | ***Megasphaera micronuciformis*** | *Streptococcus constellatus* |
| ***Cardiobacterium valvarum*** | *Megasphaera* spp*.* | *Streptococcus cristatus* |
| ***Centipedia periodontii*** | ***Mogibacterium timidum*** | *Streptococcus gordonii* |
| ***Corynebacterium durum*** | ***Neisseria bacilliformis*** | *Streptococcus intermedius* |
| *Corynebacterium matruchotii* | *Neisseria cinerea* | *Streptococcus mitis* |
| *Corynebacterium* spp. | *Neisseria elongata* | *Streptococcus mutans* |
| *Cryptobacterium curtum* | ***Neisseria macacae/ mucosa*** | *Streptococcus oralis* |
| ***Dialister invisus*** | ***Neisseria oralis*** | *Streptococcus parasanguinis* |
| ***Dialister pneumosintes*** | *Neisseria* spp*.* | *Streptococcus salivarius* |
| *Eikenella corrodens* | *Neisseria subflava/ flavescencs/ perflava* | *Streptococcus sanguinis* |
| *Eikenella/ Kingella spp.* | ***Olsenella profusa*** | ***Streptococcus sinensis*** |
| *Enterobacteriaceae spp.* | *Olsenella uli* | *Streptococcus sobrinus* |
| *Eubacteirum/ Mogibacterium spp.* | *Ottowia/Pasteurella* spp*.* | *Streptococcus* spp. |
| ***Eubacterium infirmum*** | *Parascardovia denticolens* | *Streptococcus vestibularis* |
| ***Eubacterium yurii*** | *Parvimonas micra* | *Tannerella forsythia* |
| *Filifactor alocis* | ***Peptidophaga* spp.** | *Tannerella* spp. |
| ***Fusobacterium canifelinum*** | *Peptoniphilus* spp*.* | ***Veillonella atypica*** |
| *Fusobacterium hwasooki* | ***Peptostreptococcus* spp*.*** | *Veillonella parvula* |
| ***Fusobacterium naviforme*** | ***Peptostreptococcus stomatis*** | *Veillonella* spp. |
| *Fusobacterium nucleatum* | *Porphyromonas gingivalis* |  |

**Table S2:** Log fold change of bacterial species abundance found in oral biofilm samples of 179 study participants with metagenomics sequencing. Comparison of the three study groups, healthy (H), caries (C) and periodontitis (P).

| **Periodontitis versus Healthy** |  | **Caries versus healthy** |  | **Periodontitis versus Caries** |  |
| --- | --- | --- | --- | --- | --- |
| **Species** | **Log fold change** | **Species** | **Log fold change** | **Species** | **Log fold change** |
| *Desulfobulbus oralis* | 9.84 | *Propionibacterium acidifaciens* | 7.42 | *Desulfobulbus oralis* | 8.75 |
| *Tannerella forsythia* | 8.05 | *Streptococcus mutans* | 4.32 | *Porphyromonas gingivalis* | 7.54 |
| *Porphyromonas gingivalis* | 7.98 | *Leptotrichia wadei* | 1.54 | *Tannerella forsythia* | 7.25 |
| *Treponema denticola* | 7.07 | *Actinomyces* sp. oral taxon 897 | 1.23 | *Treponema denticola* | 6.12 |
| *Filifactor alocis* | 6.81 | *Actinomyces oris* | 1.00 | *Filifactor alocis* | 5.56 |
| *Treponema sp. OMZ 838* | 4.43 | *Actinomyces naeslundii* | 0.986 | *Treponema* sp. OMZ 838 | 4.28 |
| *Parvimonas micra* | 4.00 | *Actinomyces* sp. oral taxon 414 | 0.852 | *Parvimonas micra* | 3.85 |
| *Prevotella intermedia* | 2.64 |  |  | *Prevotella intermedia* | 2.14 |
| *Fusobacterium nucleatum* | 1.51 |  |  | *Fusobacterium nucleatum* | 1.53 |
| *Prevotella oralis* | 0.916 |  |  | *Prevotella oralis* | 1.06 |
|  |  |  |  |  |  |
| *Lautropia mirabilis* | -3.86 | *Neisseria elongata* | -1.62 | *Propionibacterium acidifaciens* | -5.52 |
| *Rothia dentocariosa* | -3.39 | *Neisseria mucosa* | -1.57 | *Streptococcus mutans* | -4.22 |
| *Streptococcus sanguinis* | -2.99 | *Cardiobacterium hominis* | -1.38 | *Actinomyces oris* | -3.79 |
| *Actinomyces naeslundii* | -2.80 | *Haemophilus parainfluenzae* | -1.24 | *Actinomyces naeslundii* | -3.79 |
| *Actinomyces oris* | -2.79 | *Capnocytophaga sputigena* | -0.807 | *Rothia dentocariosa* | -3.18 |
| *Corynebacterium matruchotii* | -2.77 |  |  | *Actinomyces* sp. oral taxon 171 | -3.09 |
| *Actinomyces* sp. oral taxon 171 | -2.73 |  |  | *Corynebacterium matruchotii* | -2.74 |
| *Haemophilus parainfluenzae* | -2.41 |  |  | *Lautropia mirabilis* | -2.66 |
| *Actinomyces viscosus* | -2.38 |  |  | *Actinomyces viscosus* | -2.58 |
| *Streptococcus oralis* | -2.37 |  |  | *Streptococcus sanguinis* | -2.38 |

**Table S3:** ARGs found in oral biofilm samples of 179 study participants (H, C, P) with metagenomics sequencing and corresponding antibiotics they confer resistance to.

| **Antibiotic** | **ARG** |
| --- | --- |
| Acridine Dye | hmrM |
| Amikacin | aph(3')-IIIa |
| Aminoglycoside | ANT(3'')-IIa |
| Amoxicillin | blaTEM-1A_1, blaTEM-1C_1 |
| Ampicillin | blaTEM-1A_1, blaTEM-1C_1 |
| Antibacterial Free Fatty Acids | farA, farB |
| Beta Lactam | blaOXA-85, blaTEM-1, blaTEM-2, cfxA, cfxA_gen, cfxA3 |
| Bleomycin | ble_Tn5 |
| Cephalosporin | blaCSP-1, blaOXA-1, TEM-1 |
| Cephalothin | blaTEM-1A_1, blaTEM-1C_1 |
| Chloramphenicol | catA16, catD |
| Clindamycin | erm(B)_18 |
| Erythromycin | erm(B)_18 |
| Fluoroquinolone | hmrM, patA, patB, pmrA |
| Kanamycin | aph(3')-Ia, aph(3')-Ib, aph(3')-IIa, aph(3')-IIIa |
| Lincomycin | erm(B)_18 |
| Lincosamide | lnu(C), lsa(C), RlmA(II) |
| Macrolide | erm(A), erm(B), erm(F), erm(X), mef(A), msr(D), mtrC, mtrE, RlmA(II) |
| Monobactam | TEM-1 |
| Nitroimidazole | nimI |
| Penam | mtrC, mtrE, TEM-1 |
| Penem | TEM-1 |
| Peptide | pgpB |
| Phenicol | catS |
| Piperacillin | blaTEM-1A_1, blaTEM-1C_1 |
| Pristinamycin_IA | erm(B)_18 |
| Quinupristin | erm(B)_18 |
| Rifamycin | Bifidobacterium_adolescentis_rpoB_conferring_resistance_to_rifampicin |
| Streptogramin | lsa(C) |
| Streptomycin | ant(6)-Ia, aph(3'')-Ib, aph(6)-Ic, aph(6)-Id, |
| Streptothricin | sat4 |
| Sulfamethoxazole | sul1_5 |
| Sulfonamide | sul1, sul2 |
| Tetracycline | tet(32), tet(B), tet(M), tet(O), tet(Q), tet(W), tet(X), tetA(46), tetA(60), tetB(46), tetB(60) |
| Tigecyline | tet(Q), tet(X) |
| Ticarcillin | blaTEM-1A_1, blaTEM-1C_1 |
| Virginiamycin_S | erm(B)_18 |

**Table S4:**

Bacterial species that were assigned to the ARGs found in oral biofilm samples of 179 study participants with metagenomics sequencing. Only assignments that could be made to the species level are included in the table.

| ARG | Bacteroides fragilis | Bifidobacterium dentium | Campylobacter concisus | Capnocytophaga oraltaxon878 | Capnocytophaga sputigena | Escherichia coli | Eubacterium minutum | Gardnerella vaginalis | Gemella oral taxon 928 | Haemophilus ducreyi | Haemophilus oral taxon 036 | Haemophilus parainfluenzae | Lachnospiraceae bacterium | Lactobacillus reuteri | Lautropia mirabilis | Leptotrichia wadei | Neisseria cinerea | Neisseria elongata | Neisseria mucosa | Porphyromonas asaccharolytica | Porphyromonas gingivalis | Prevotella intermedia | Prevotella melaninogenica | Rothia mucilaginosa | Schaalia odontolytica | Selenomonas oral taxon 136 | Sphingobacterium hotanense | Streptococcus sp. 1643 | Streptococcus agalactiae | Streptococcus anginosus | Streptococcus australis | Streptococcus sp. ChDC | Streptococcus cristatus | Streptococcus sp. FDAARGOS_522 | Streptococcus gordonii | Streptococcus sp. HSISM1 | Streptococcus sp. HSISS3 | Streptococcus intermedius | Streptococcus sp. LPB0220 | Streptococcus mitis | Streptococcus oralis | Streptococcus oral taxon 431 | Streptococcus parasanguinis | Streptococcus pneumoniae | Streptococcus sanguinis | Tannerella forsythia | Veillonella dispar | Veillonella parvula |
| --- | --- | --- | --- | --- | --- | --- | --- | --- | --- | --- | --- | --- | --- | --- | --- | --- | --- | --- | --- | --- | --- | --- | --- | --- | --- | --- | --- | --- | --- | --- | --- | --- | --- | --- | --- | --- | --- | --- | --- | --- | --- | --- | --- | --- | --- | --- | --- | --- |
| (AGly)aadA1-pm |  |  |  |  |  |  |  |  |  |  |  |  |  |  |  |  |  |  |  |  |  |  |  |  |  |  |  |  |  |  |  |  |  |  |  |  |  |  |  |  |  |  |  |  |  |  |  |  |
| (Bla)PBP1b |  |  |  |  |  |  |  |  |  |  |  |  |  |  |  |  |  |  |  |  |  |  |  |  |  |  |  |  |  |  |  |  |  |  |  |  |  |  |  |  |  |  |  |  |  |  |  |  |
| (Tet)tetA |  |  |  |  |  |  |  |  |  |  |  |  |  |  |  |  |  |  |  |  |  |  |  |  |  |  |  |  |  |  |  |  |  |  |  |  |  |  |  |  |  |  |  |  |  |  |  |  |
| (Tet)tetR |  |  |  |  |  |  |  |  |  |  |  |  |  |  |  |  |  |  |  |  |  |  |  |  |  |  |  |  |  |  |  |  |  |  |  |  |  |  |  |  |  |  |  |  |  |  |  |  |
| ANT(3'')-IIa |  |  |  |  |  |  |  |  |  |  |  |  |  |  |  |  |  |  |  |  |  |  |  |  |  |  |  |  |  |  |  |  |  |  |  |  |  |  |  |  |  |  |  |  |  |  |  |  |
| ant(6)-Ia |  |  |  |  |  |  |  |  |  |  |  |  |  |  |  |  |  |  |  |  |  |  |  |  |  |  |  |  |  |  |  |  |  |  |  |  |  |  |  |  |  |  |  |  |  |  |  |  |
| aph(3')-Ia |  |  |  |  |  |  |  |  |  |  |  |  |  |  |  |  |  |  |  |  |  |  |  |  |  |  |  |  |  |  |  |  |  |  |  |  |  |  |  |  |  |  |  |  |  |  |  |  |
| aph(3')-Ib |  |  |  |  |  |  |  |  |  |  |  |  |  |  |  |  |  |  |  |  |  |  |  |  |  |  |  |  |  |  |  |  |  |  |  |  |  |  |  |  |  |  |  |  |  |  |  |  |
| aph(3'')-Ib |  |  |  |  |  |  |  |  |  |  |  |  |  |  |  |  |  |  |  |  |  |  |  |  |  |  |  |  |  |  |  |  |  |  |  |  |  |  |  |  |  |  |  |  |  |  |  |  |
| aph(3')-IIa |  |  |  |  |  |  |  |  |  |  |  |  |  |  |  |  |  |  |  |  |  |  |  |  |  |  |  |  |  |  |  |  |  |  |  |  |  |  |  |  |  |  |  |  |  |  |  |  |
| aph(3')-IIIa |  |  |  |  |  |  |  |  |  |  |  |  |  |  |  |  |  |  |  |  |  |  |  |  |  |  |  |  |  |  |  |  |  |  |  |  |  |  |  |  |  |  |  |  |  |  |  |  |
| aph(6)-Ic |  |  |  |  |  |  |  |  |  |  |  |  |  |  |  |  |  |  |  |  |  |  |  |  |  |  |  |  |  |  |  |  |  |  |  |  |  |  |  |  |  |  |  |  |  |  |  |  |
| aph(6)-Id |  |  |  |  |  |  |  |  |  |  |  |  |  |  |  |  |  |  |  |  |  |  |  |  |  |  |  |  |  |  |  |  |  |  |  |  |  |  |  |  |  |  |  |  |  |  |  |  |
| Bifido_rpoB |  |  |  |  |  |  |  |  |  |  |  |  |  |  |  |  |  |  |  |  |  |  |  |  |  |  |  |  |  |  |  |  |  |  |  |  |  |  |  |  |  |  |  |  |  |  |  |  |
| blaCSP-1 |  |  |  |  |  |  |  |  |  |  |  |  |  |  |  |  |  |  |  |  |  |  |  |  |  |  |  |  |  |  |  |  |  |  |  |  |  |  |  |  |  |  |  |  |  |  |  |  |
| blaOXA-1 |  |  |  |  |  |  |  |  |  |  |  |  |  |  |  |  |  |  |  |  |  |  |  |  |  |  |  |  |  |  |  |  |  |  |  |  |  |  |  |  |  |  |  |  |  |  |  |  |
| blaOXA-85 |  |  |  |  |  |  |  |  |  |  |  |  |  |  |  |  |  |  |  |  |  |  |  |  |  |  |  |  |  |  |  |  |  |  |  |  |  |  |  |  |  |  |  |  |  |  |  |  |
| blaTEM-1 |  |  |  |  |  |  |  |  |  |  |  |  |  |  |  |  |  |  |  |  |  |  |  |  |  |  |  |  |  |  |  |  |  |  |  |  |  |  |  |  |  |  |  |  |  |  |  |  |
| blaTEM-1A_1 |  |  |  |  |  |  |  |  |  |  |  |  |  |  |  |  |  |  |  |  |  |  |  |  |  |  |  |  |  |  |  |  |  |  |  |  |  |  |  |  |  |  |  |  |  |  |  |  |
| blaTEM-1C_1 |  |  |  |  |  |  |  |  |  |  |  |  |  |  |  |  |  |  |  |  |  |  |  |  |  |  |  |  |  |  |  |  |  |  |  |  |  |  |  |  |  |  |  |  |  |  |  |  |
| blaTEM-2 |  |  |  |  |  |  |  |  |  |  |  |  |  |  |  |  |  |  |  |  |  |  |  |  |  |  |  |  |  |  |  |  |  |  |  |  |  |  |  |  |  |  |  |  |  |  |  |  |
| ble_Tn5 |  |  |  |  |  |  |  |  |  |  |  |  |  |  |  |  |  |  |  |  |  |  |  |  |  |  |  |  |  |  |  |  |  |  |  |  |  |  |  |  |  |  |  |  |  |  |  |  |
| catA16 |  |  |  |  |  |  |  |  |  |  |  |  |  |  |  |  |  |  |  |  |  |  |  |  |  |  |  |  |  |  |  |  |  |  |  |  |  |  |  |  |  |  |  |  |  |  |  |  |
| catD |  |  |  |  |  |  |  |  |  |  |  |  |  |  |  |  |  |  |  |  |  |  |  |  |  |  |  |  |  |  |  |  |  |  |  |  |  |  |  |  |  |  |  |  |  |  |  |  |
| catS |  |  |  |  |  |  |  |  |  |  |  |  |  |  |  |  |  |  |  |  |  |  |  |  |  |  |  |  |  |  |  |  |  |  |  |  |  |  |  |  |  |  |  |  |  |  |  |  |
| cfxA |  |  |  |  |  |  |  |  |  |  |  |  |  |  |  |  |  |  |  |  |  |  |  |  |  |  |  |  |  |  |  |  |  |  |  |  |  |  |  |  |  |  |  |  |  |  |  |  |
| cfxA_gen |  |  |  |  |  |  |  |  |  |  |  |  |  |  |  |  |  |  |  |  |  |  |  |  |  |  |  |  |  |  |  |  |  |  |  |  |  |  |  |  |  |  |  |  |  |  |  |  |
| cfxA3 |  |  |  |  |  |  |  |  |  |  |  |  |  |  |  |  |  |  |  |  |  |  |  |  |  |  |  |  |  |  |  |  |  |  |  |  |  |  |  |  |  |  |  |  |  |  |  |  |
| erm(A) |  |  |  |  |  |  |  |  |  |  |  |  |  |  |  |  |  |  |  |  |  |  |  |  |  |  |  |  |  |  |  |  |  |  |  |  |  |  |  |  |  |  |  |  |  |  |  |  |
| erm(B) |  |  |  |  |  |  |  |  |  |  |  |  |  |  |  |  |  |  |  |  |  |  |  |  |  |  |  |  |  |  |  |  |  |  |  |  |  |  |  |  |  |  |  |  |  |  |  |  |
| erm(B)_18 |  |  |  |  |  |  |  |  |  |  |  |  |  |  |  |  |  |  |  |  |  |  |  |  |  |  |  |  |  |  |  |  |  |  |  |  |  |  |  |  |  |  |  |  |  |  |  |  |
| erm(F) |  |  |  |  |  |  |  |  |  |  |  |  |  |  |  |  |  |  |  |  |  |  |  |  |  |  |  |  |  |  |  |  |  |  |  |  |  |  |  |  |  |  |  |  |  |  |  |  |
| erm(X) |  |  |  |  |  |  |  |  |  |  |  |  |  |  |  |  |  |  |  |  |  |  |  |  |  |  |  |  |  |  |  |  |  |  |  |  |  |  |  |  |  |  |  |  |  |  |  |  |
| farA |  |  |  |  |  |  |  |  |  |  |  |  |  |  |  |  |  |  |  |  |  |  |  |  |  |  |  |  |  |  |  |  |  |  |  |  |  |  |  |  |  |  |  |  |  |  |  |  |
| farB |  |  |  |  |  |  |  |  |  |  |  |  |  |  |  |  |  |  |  |  |  |  |  |  |  |  |  |  |  |  |  |  |  |  |  |  |  |  |  |  |  |  |  |  |  |  |  |  |
| hmrM |  |  |  |  |  |  |  |  |  |  |  |  |  |  |  |  |  |  |  |  |  |  |  |  |  |  |  |  |  |  |  |  |  |  |  |  |  |  |  |  |  |  |  |  |  |  |  |  |
| lnu(C) |  |  |  |  |  |  |  |  |  |  |  |  |  |  |  |  |  |  |  |  |  |  |  |  |  |  |  |  |  |  |  |  |  |  |  |  |  |  |  |  |  |  |  |  |  |  |  |  |
| lsa(C) |  |  |  |  |  |  |  |  |  |  |  |  |  |  |  |  |  |  |  |  |  |  |  |  |  |  |  |  |  |  |  |  |  |  |  |  |  |  |  |  |  |  |  |  |  |  |  |  |
| mef(A) |  |  |  |  |  |  |  |  |  |  |  |  |  |  |  |  |  |  |  |  |  |  |  |  |  |  |  |  |  |  |  |  |  |  |  |  |  |  |  |  |  |  |  |  |  |  |  |  |
| msr(D) |  |  |  |  |  |  |  |  |  |  |  |  |  |  |  |  |  |  |  |  |  |  |  |  |  |  |  |  |  |  |  |  |  |  |  |  |  |  |  |  |  |  |  |  |  |  |  |  |
| mtrC |  |  |  |  |  |  |  |  |  |  |  |  |  |  |  |  |  |  |  |  |  |  |  |  |  |  |  |  |  |  |  |  |  |  |  |  |  |  |  |  |  |  |  |  |  |  |  |  |
| mtrE |  |  |  |  |  |  |  |  |  |  |  |  |  |  |  |  |  |  |  |  |  |  |  |  |  |  |  |  |  |  |  |  |  |  |  |  |  |  |  |  |  |  |  |  |  |  |  |  |
| nimI |  |  |  |  |  |  |  |  |  |  |  |  |  |  |  |  |  |  |  |  |  |  |  |  |  |  |  |  |  |  |  |  |  |  |  |  |  |  |  |  |  |  |  |  |  |  |  |  |
| patA |  |  |  |  |  |  |  |  |  |  |  |  |  |  |  |  |  |  |  |  |  |  |  |  |  |  |  |  |  |  |  |  |  |  |  |  |  |  |  |  |  |  |  |  |  |  |  |  |
| patB |  |  |  |  |  |  |  |  |  |  |  |  |  |  |  |  |  |  |  |  |  |  |  |  |  |  |  |  |  |  |  |  |  |  |  |  |  |  |  |  |  |  |  |  |  |  |  |  |
| pgpB |  |  |  |  |  |  |  |  |  |  |  |  |  |  |  |  |  |  |  |  |  |  |  |  |  |  |  |  |  |  |  |  |  |  |  |  |  |  |  |  |  |  |  |  |  |  |  |  |
| pmrA |  |  |  |  |  |  |  |  |  |  |  |  |  |  |  |  |  |  |  |  |  |  |  |  |  |  |  |  |  |  |  |  |  |  |  |  |  |  |  |  |  |  |  |  |  |  |  |  |
| RlmA(II) |  |  |  |  |  |  |  |  |  |  |  |  |  |  |  |  |  |  |  |  |  |  |  |  |  |  |  |  |  |  |  |  |  |  |  |  |  |  |  |  |  |  |  |  |  |  |  |  |
| sat4 |  |  |  |  |  |  |  |  |  |  |  |  |  |  |  |  |  |  |  |  |  |  |  |  |  |  |  |  |  |  |  |  |  |  |  |  |  |  |  |  |  |  |  |  |  |  |  |  |
| sul1 |  |  |  |  |  |  |  |  |  |  |  |  |  |  |  |  |  |  |  |  |  |  |  |  |  |  |  |  |  |  |  |  |  |  |  |  |  |  |  |  |  |  |  |  |  |  |  |  |
| sul1_5 |  |  |  |  |  |  |  |  |  |  |  |  |  |  |  |  |  |  |  |  |  |  |  |  |  |  |  |  |  |  |  |  |  |  |  |  |  |  |  |  |  |  |  |  |  |  |  |  |
| sul2 |  |  |  |  |  |  |  |  |  |  |  |  |  |  |  |  |  |  |  |  |  |  |  |  |  |  |  |  |  |  |  |  |  |  |  |  |  |  |  |  |  |  |  |  |  |  |  |  |
| TEM-1 |  |  |  |  |  |  |  |  |  |  |  |  |  |  |  |  |  |  |  |  |  |  |  |  |  |  |  |  |  |  |  |  |  |  |  |  |  |  |  |  |  |  |  |  |  |  |  |  |
| tet(32) |  |  |  |  |  |  |  |  |  |  |  |  |  |  |  |  |  |  |  |  |  |  |  |  |  |  |  |  |  |  |  |  |  |  |  |  |  |  |  |  |  |  |  |  |  |  |  |  |
| tet(B) |  |  |  |  |  |  |  |  |  |  |  |  |  |  |  |  |  |  |  |  |  |  |  |  |  |  |  |  |  |  |  |  |  |  |  |  |  |  |  |  |  |  |  |  |  |  |  |  |
| tet(M) |  |  |  |  |  |  |  |  |  |  |  |  |  |  |  |  |  |  |  |  |  |  |  |  |  |  |  |  |  |  |  |  |  |  |  |  |  |  |  |  |  |  |  |  |  |  |  |  |
| tet(O) |  |  |  |  |  |  |  |  |  |  |  |  |  |  |  |  |  |  |  |  |  |  |  |  |  |  |  |  |  |  |  |  |  |  |  |  |  |  |  |  |  |  |  |  |  |  |  |  |
| tet(Q) |  |  |  |  |  |  |  |  |  |  |  |  |  |  |  |  |  |  |  |  |  |  |  |  |  |  |  |  |  |  |  |  |  |  |  |  |  |  |  |  |  |  |  |  |  |  |  |  |
| tet(W) |  |  |  |  |  |  |  |  |  |  |  |  |  |  |  |  |  |  |  |  |  |  |  |  |  |  |  |  |  |  |  |  |  |  |  |  |  |  |  |  |  |  |  |  |  |  |  |  |
| tet(X) |  |  |  |  |  |  |  |  |  |  |  |  |  |  |  |  |  |  |  |  |  |  |  |  |  |  |  |  |  |  |  |  |  |  |  |  |  |  |  |  |  |  |  |  |  |  |  |  |
| tetA(46) |  |  |  |  |  |  |  |  |  |  |  |  |  |  |  |  |  |  |  |  |  |  |  |  |  |  |  |  |  |  |  |  |  |  |  |  |  |  |  |  |  |  |  |  |  |  |  |  |
| tetA(60) |  |  |  |  |  |  |  |  |  |  |  |  |  |  |  |  |  |  |  |  |  |  |  |  |  |  |  |  |  |  |  |  |  |  |  |  |  |  |  |  |  |  |  |  |  |  |  |  |
| tetB(46) |  |  |  |  |  |  |  |  |  |  |  |  |  |  |  |  |  |  |  |  |  |  |  |  |  |  |  |  |  |  |  |  |  |  |  |  |  |  |  |  |  |  |  |  |  |  |  |  |
| tetB(60) |  |  |  |  |  |  |  |  |  |  |  |  |  |  |  |  |  |  |  |  |  |  |  |  |  |  |  |  |  |  |  |  |  |  |  |  |  |  |  |  |  |  |  |  |  |  |  |  |

**Table S5:**

Bacterial species (found in oral biofilm of 179 study participants) associated with resistance to the phenotypically-tested antibiotics in both the phenotypic (E-test) and metagenomic sequencing methods. Red: phenotypic resistance; blue: genotypic resistance; yellow: both methods found resistance. The species highlighted in grey are those that were tested in the phenotypic study.

| Species Name | Ampicillin | Azithromycin | Cefuroxim | Ciprofloxacin | Clindamycin | Colistin | Erythromycin | Fosfomycin | Gentamycin | Meropenem | Metronidazol | Moxifloxacin | Penicillin G | Tetracycline | Tigecycline | Vancomycin |
| --- | --- | --- | --- | --- | --- | --- | --- | --- | --- | --- | --- | --- | --- | --- | --- | --- |
| *Actinomyces oris* |  |  |  |  |  |  |  |  |  |  |  |  |  |  |  |  |
| *Aggregatibacter actinomycetemcomitans* |  |  |  |  |  |  |  |  |  |  |  |  |  |  |  |  |
| *Bacteroides fragilis* |  |  |  |  |  |  |  |  |  |  |  |  |  |  |  |  |
| *Bifidobacterium dentium* |  |  |  |  |  |  |  |  |  |  |  |  |  |  |  |  |
| *Campylobacter concisus* |  |  |  |  |  |  |  |  |  |  |  |  |  |  |  |  |
| *Capnocytophaga ochracea* |  |  |  |  |  |  |  |  |  |  |  |  |  |  |  |  |
| *Capnocytophaga* sp. oral taxon 878 |  |  |  |  |  |  |  |  |  |  |  |  |  |  |  |  |
| *Capnocytophaga sputigena* |  |  |  |  |  |  |  |  |  |  |  |  |  |  |  |  |
| *Eikenella corrodens* |  |  |  |  |  |  |  |  |  |  |  |  |  |  |  |  |
| *Escherichia coli* |  |  |  |  |  |  |  |  |  |  |  |  |  |  |  |  |
| *Eubacterium minutum* |  |  |  |  |  |  |  |  |  |  |  |  |  |  |  |  |
| *Fusobacterium nucleatum* |  |  |  |  |  |  |  |  |  |  |  |  |  |  |  |  |
| *Gardnerella vaginalis* |  |  |  |  |  |  |  |  |  |  |  |  |  |  |  |  |
| *Gemella* sp. oral taxon 928 |  |  |  |  |  |  |  |  |  |  |  |  |  |  |  |  |
| *Haemophilus ducreyi* |  |  |  |  |  |  |  |  |  |  |  |  |  |  |  |  |
| *Haemophilus* sp. oral taxon 036 |  |  |  |  |  |  |  |  |  |  |  |  |  |  |  |  |
| *Haemophilus parainfluenzae* |  |  |  |  |  |  |  |  |  |  |  |  |  |  |  |  |
| *Lachnoanaerobaculum saburreum* |  |  |  |  |  |  |  |  |  |  |  |  |  |  |  |  |
| *Lachnospiraceae bacterium* |  |  |  |  |  |  |  |  |  |  |  |  |  |  |  |  |
| *Lactobacillus reuteri* |  |  |  |  |  |  |  |  |  |  |  |  |  |  |  |  |
| *Lautropia mirabilis* |  |  |  |  |  |  |  |  |  |  |  |  |  |  |  |  |
| *Leptotrichia wadei* |  |  |  |  |  |  |  |  |  |  |  |  |  |  |  |  |
| *Neisseria cinerea* |  |  |  |  |  |  |  |  |  |  |  |  |  |  |  |  |
| *Neisseria elongata* |  |  |  |  |  |  |  |  |  |  |  |  |  |  |  |  |
| *Neisseria macacae* |  |  |  |  |  |  |  |  |  |  |  |  |  |  |  |  |
| *Neisseria mucosa* |  |  |  |  |  |  |  |  |  |  |  |  |  |  |  |  |
| *Parvimonas micra* |  |  |  |  |  |  |  |  |  |  |  |  |  |  |  |  |
| *Porphyromonas asaccharolytica* |  |  |  |  |  |  |  |  |  |  |  |  |  |  |  |  |
| *Porphyromonas gingivalis* |  |  |  |  |  |  |  |  |  |  |  |  |  |  |  |  |
| *Prevotella intermedia* |  |  |  |  |  |  |  |  |  |  |  |  |  |  |  |  |
| *Prevotella melaninogenica* |  |  |  |  |  |  |  |  |  |  |  |  |  |  |  |  |
| *Prevotella nigrescens* |  |  |  |  |  |  |  |  |  |  |  |  |  |  |  |  |
| *Rothia mucilaginosa* |  |  |  |  |  |  |  |  |  |  |  |  |  |  |  |  |
| *Schaalia odontolytica* |  |  |  |  |  |  |  |  |  |  |  |  |  |  |  |  |
| *Selenomonas* sp. oral taxon 136 |  |  |  |  |  |  |  |  |  |  |  |  |  |  |  |  |
| *Sphingobacterium hotanense* |  |  |  |  |  |  |  |  |  |  |  |  |  |  |  |  |
| *Streptococcus* sp*. 1643* |  |  |  |  |  |  |  |  |  |  |  |  |  |  |  |  |
| *Streptococcus agalactiae* |  |  |  |  |  |  |  |  |  |  |  |  |  |  |  |  |
| *Streptococcus anginosus* |  |  |  |  |  |  |  |  |  |  |  |  |  |  |  |  |
| *Streptococcus australis* |  |  |  |  |  |  |  |  |  |  |  |  |  |  |  |  |
| *Streptococcus* sp*. ChDC* |  |  |  |  |  |  |  |  |  |  |  |  |  |  |  |  |
| *Streptococcus constellatus* |  |  |  |  |  |  |  |  |  |  |  |  |  |  |  |  |
| *Streptococcus cristatus* |  |  |  |  |  |  |  |  |  |  |  |  |  |  |  |  |
| *Streptococcus* sp. *FDAARGOS_522* |  |  |  |  |  |  |  |  |  |  |  |  |  |  |  |  |
| *Streptococcus gordonii* |  |  |  |  |  |  |  |  |  |  |  |  |  |  |  |  |
| *Streptococcus* sp. *HSISM1* |  |  |  |  |  |  |  |  |  |  |  |  |  |  |  |  |
| *Streptococcus* sp. *HSISS3* |  |  |  |  |  |  |  |  |  |  |  |  |  |  |  |  |
| *Streptococcus infantis* |  |  |  |  |  |  |  |  |  |  |  |  |  |  |  |  |
| *Streptococcus intermedius* |  |  |  |  |  |  |  |  |  |  |  |  |  |  |  |  |
| *Streptococcus* sp*. LPB0220* |  |  |  |  |  |  |  |  |  |  |  |  |  |  |  |  |
| *Streptococcus mitis* |  |  |  |  |  |  |  |  |  |  |  |  |  |  |  |  |
| *Streptococcus mutans* |  |  |  |  |  |  |  |  |  |  |  |  |  |  |  |  |
| *Streptococcus oralis* |  |  |  |  |  |  |  |  |  |  |  |  |  |  |  |  |
| *Streptococcus* sp. oral taxon 431 |  |  |  |  |  |  |  |  |  |  |  |  |  |  |  |  |
| *Streptococcus parasanguinis* |  |  |  |  |  |  |  |  |  |  |  |  |  |  |  |  |
| *Streptococcus pneumoniae* |  |  |  |  |  |  |  |  |  |  |  |  |  |  |  |  |
| *Streptococcus sanguinis* |  |  |  |  |  |  |  |  |  |  |  |  |  |  |  |  |
| *Tannerella forsythia* |  |  |  |  |  |  |  |  |  |  |  |  |  |  |  |  |
| *Veillonella dispar* |  |  |  |  |  |  |  |  |  |  |  |  |  |  |  |  |
| *Veillonella parvula* |  |  |  |  |  |  |  |  |  |  |  |  |  |  |  |  |

**Table S6:** The mean relative abundances (in %) of species found through metagenomic sequencing. The mean relative abundance in healthy, caries and periodontitis samples are reported separately, along with the overall mean across all groups. Only the species with a mean overall abundance >0.1% are listed.

| ***Taxa*** | **Healthy** | **Caries** | **Periodontitis** | **Overall** |
| --- | --- | --- | --- | --- |
| *Actinomyces sp. oral taxon 414* | 4.489 | 7.129 | 3.243 | 5.006 |
| *Veillonella parvula* | 5.508 | 6.254 | 2.341 | 4.789 |
| *Corynebacterium matruchotii* | 6.388 | 6.051 | 1.313 | 4.714 |
| *Actinomyces sp. oral taxon 171* | 5.152 | 5.565 | 1.177 | 4.072 |
| *Streptococcus oralis* | 5.360 | 3.890 | 1.629 | 3.713 |
| *Porphyromonas gingivalis* | 0.041 | 0.059 | 11.962 | 3.710 |
| *Rothia dentocariosa* | 5.861 | 4.076 | 0.779 | 3.691 |
| *Fusobacterium nucleatum* | 1.873 | 1.657 | 6.493 | 3.219 |
| *Streptococcus sanguinis* | 5.262 | 2.800 | 0.930 | 3.092 |
| *Tannerella forsythia* | 0.034 | 0.053 | 9.886 | 3.068 |
| *Actinomyces viscosus* | 3.684 | 3.647 | 0.987 | 2.843 |
| *Actinomyces oris* | 2.674 | 4.388 | 0.636 | 2.632 |
| *Actinomyces naeslundii* | 2.315 | 3.760 | 0.545 | 2.264 |
| *Streptococcus gordonii* | 2.058 | 3.198 | 1.284 | 2.209 |
| *Neisseria mucosa* | 3.559 | 1.293 | 1.277 | 2.085 |
| *Lautropia mirabilis* | 3.597 | 1.456 | 0.344 | 1.868 |
| *Prevotella intermedia* | 0.488 | 0.613 | 3.833 | 1.558 |
| *Treponema denticola* | 0.032 | 0.055 | 4.962 | 1.555 |
| *Haemophilus parainfluenzae* | 2.641 | 1.101 | 0.695 | 1.518 |
| *Propionibacterium acidifaciens* | 0.037 | 4.185 | 0.172 | 1.492 |
| *Actinomyces sp. oral taxon 897* | 0.766 | 1.655 | 0.969 | 1.131 |
| *Neisseria elongata* | 1.738 | 0.554 | 0.412 | 0.927 |
| *Desulfobulbus oralis* | 0.002 | 0.005 | 2.908 | 0.896 |
| *Parvimonas micra* | 0.139 | 0.131 | 2.610 | 0.895 |
| *Treponema sp. OMZ 838* | 0.095 | 0.090 | 2.322 | 0.777 |
| *Actinomyces hongkongensis* | 0.881 | 1.026 | 0.375 | 0.775 |
| *Capnocytophaga sputigena* | 1.234 | 0.653 | 0.338 | 0.761 |
| *Tannerella sp. oral taxon HOT-286* | 0.929 | 0.791 | 0.416 | 0.724 |
| *Pseudopropionibacterium propionicum* | 0.979 | 0.640 | 0.499 | 0.716 |
| *Streptococcus cristatus* | 0.761 | 0.859 | 0.475 | 0.706 |
| *Schaalia odontolytica* | 0.658 | 0.975 | 0.378 | 0.680 |
| *Capnocytophaga gingivalis* | 1.032 | 0.566 | 0.365 | 0.668 |
| *Prevotella oris* | 0.429 | 0.372 | 1.156 | 0.633 |
| *Streptococcus mutans* | 0.124 | 1.564 | 0.174 | 0.630 |
| *Streptococcus mitis* | 0.889 | 0.453 | 0.499 | 0.621 |
| *Olsenella sp. oral taxon 807* | 0.418 | 0.560 | 0.886 | 0.610 |
| *Leptotrichia wadei* | 0.358 | 1.039 | 0.360 | 0.591 |
| *Cardiobacterium hominis* | 1.031 | 0.387 | 0.234 | 0.567 |
| *Campylobacter gracilis* | 0.499 | 0.566 | 0.619 | 0.559 |
| *Gemella morbillorum* | 0.556 | 0.598 | 0.488 | 0.550 |
| *Filifactor alocis* | 0.012 | 0.025 | 1.723 | 0.542 |
| *Prevotella denticola* | 0.289 | 0.598 | 0.722 | 0.527 |
| *Prevotella melaninogenica* | 0.406 | 0.629 | 0.516 | 0.516 |
| *Capnocytophaga leadbetteri* | 0.759 | 0.481 | 0.260 | 0.511 |
| *Actinomyces sp. oral taxon 848* | 0.389 | 0.644 | 0.495 | 0.509 |
| *Aggregatibacter aphrophilus* | 0.925 | 0.271 | 0.295 | 0.509 |
| *Neisseria sp. oral taxon 014* | 0.885 | 0.328 | 0.212 | 0.488 |
| *Selenomonas sputigena* | 0.188 | 0.391 | 0.852 | 0.461 |
| *Streptococcus intermedius* | 0.368 | 0.261 | 0.665 | 0.423 |
| *Rothia aeria* | 0.769 | 0.274 | 0.133 | 0.405 |
| *Campylobacter showae* | 0.220 | 0.155 | 0.862 | 0.395 |
| *Leptotrichia sp. oral taxon 212* | 0.480 | 0.430 | 0.207 | 0.379 |
| *Streptococcus sp. oral taxon 064* | 0.539 | 0.405 | 0.137 | 0.370 |
| *Streptococcus sp. 1643* | 0.555 | 0.358 | 0.159 | 0.366 |
| *Streptococcus sp. NPS 308* | 0.572 | 0.331 | 0.161 | 0.364 |
| *Streptococcus australis* | 0.496 | 0.373 | 0.186 | 0.359 |
| *Streptococcus pneumoniae* | 0.498 | 0.310 | 0.217 | 0.348 |
| *Neisseria subflava* | 0.499 | 0.277 | 0.200 | 0.331 |
| *Leptotrichia hofstadii* | 0.430 | 0.367 | 0.113 | 0.311 |
| *Streptococcus anginosus* | 0.098 | 0.292 | 0.536 | 0.299 |
| *Actinomyces howellii* | 0.334 | 0.355 | 0.161 | 0.288 |
| *Leptotrichia hongkongensis* | 0.405 | 0.324 | 0.107 | 0.286 |
| *Actinomyces radicidentis* | 0.330 | 0.346 | 0.152 | 0.281 |
| *Rothia mucilaginosa* | 0.204 | 0.264 | 0.360 | 0.273 |
| *Leptotrichia buccalis* | 0.291 | 0.354 | 0.152 | 0.270 |
| *Schaalia meyeri* | 0.152 | 0.221 | 0.461 | 0.270 |
| *Eikenella corrodens* | 0.349 | 0.212 | 0.232 | 0.266 |
| *Fusobacterium sp. oral taxon 203* | 0.064 | 0.039 | 0.727 | 0.259 |
| *Fusobacterium periodonticum* | 0.214 | 0.220 | 0.350 | 0.258 |
| *Actinomyces slackii* | 0.301 | 0.310 | 0.149 | 0.257 |
| *Capnocytophaga sp. ChDC OS43* | 0.343 | 0.277 | 0.125 | 0.254 |
| *Selenomonas sp. oral taxon 126* | 0.226 | 0.245 | 0.207 | 0.226 |
| *Streptococcus sobrinus* | 0.011 | 0.625 | 0.008 | 0.219 |
| *Selenomonas sp. oral taxon 920* | 0.227 | 0.242 | 0.148 | 0.208 |
| *Actinomyces sp. Chiba101* | 0.234 | 0.241 | 0.124 | 0.203 |
| *Prevotella enoeca* | 0.054 | 0.072 | 0.521 | 0.203 |
| *Aggregatibacter segnis* | 0.241 | 0.192 | 0.146 | 0.195 |
| *Veillonella dispar* | 0.186 | 0.280 | 0.107 | 0.194 |
| *Leptotrichia trevisanii* | 0.210 | 0.232 | 0.121 | 0.190 |
| *Neisseria sp. KEM232* | 0.340 | 0.132 | 0.067 | 0.185 |
| *Campylobacter concisus* | 0.179 | 0.232 | 0.132 | 0.183 |
| *Actinomyces sp. Z16* | 0.212 | 0.224 | 0.101 | 0.182 |
| *Actinomyces sp. dk561* | 0.205 | 0.220 | 0.103 | 0.179 |
| *Fusobacterium hwasookii* | 0.170 | 0.143 | 0.219 | 0.175 |
| *Aggregatibacter actinomycetemcomitans* | 0.036 | 0.016 | 0.491 | 0.169 |
| *Bifidobacterium dentium* | 0.005 | 0.261 | 0.241 | 0.165 |
| *Lachnospiraceae bacterium oral taxon 500* | 0.016 | 0.018 | 0.490 | 0.162 |
| *Capnocytophaga sp. oral taxon 323* | 0.219 | 0.153 | 0.101 | 0.160 |
| *Ottowia sp. oral taxon 894* | 0.252 | 0.147 | 0.054 | 0.155 |
| *Parascardovia denticolens* | 0.004 | 0.392 | 0.060 | 0.153 |
| *Prevotella jejuni* | 0.075 | 0.216 | 0.162 | 0.150 |
| *Streptococcus sp. ChDC B345* | 0.214 | 0.119 | 0.108 | 0.149 |
| *Atopobium parvulum* | 0.116 | 0.223 | 0.101 | 0.148 |
| *Treponema putidum* | 0.004 | 0.005 | 0.473 | 0.148 |
| *Neisseria meningitidis* | 0.255 | 0.095 | 0.081 | 0.147 |
| *Actinomyces sp. 410* | 0.168 | 0.183 | 0.082 | 0.146 |
| *Streptococcus milleri* | 0.032 | 0.098 | 0.313 | 0.141 |
| *TM7 phylum sp. oral taxon 488* | 0.119 | 0.130 | 0.174 | 0.140 |
| *Capnocytophaga sp. oral taxon 864* | 0.165 | 0.135 | 0.108 | 0.137 |
| *Streptococcus sp. oral taxon 431* | 0.171 | 0.141 | 0.086 | 0.135 |
| *Anaerolineaceae bacterium oral taxon 439* | 0.001 | 0.002 | 0.419 | 0.130 |
| *Desulfomicrobium orale* | 0.001 | 0.001 | 0.422 | 0.130 |
| *Lachnoanaerobaculum umeaense* | 0.129 | 0.160 | 0.092 | 0.128 |
| *Gemella haemolysans* | 0.153 | 0.099 | 0.121 | 0.124 |
| *Streptococcus sp. HSISM1* | 0.020 | 0.276 | 0.068 | 0.122 |
| *Streptococcus sp. LPB0220* | 0.019 | 0.268 | 0.070 | 0.120 |
| *Streptococcus parasanguinis* | 0.028 | 0.252 | 0.072 | 0.118 |
| *Prevotella fusca* | 0.038 | 0.077 | 0.252 | 0.117 |
| *Olsenella uli* | 0.006 | 0.052 | 0.308 | 0.115 |
| *Actinomyces gaoshouyii* | 0.132 | 0.135 | 0.065 | 0.113 |
| *Prevotella scopos* | 0.061 | 0.114 | 0.170 | 0.113 |
| *Dialister pneumosintes* | 0.009 | 0.018 | 0.326 | 0.110 |
| *Prevotella dentalis* | 0.021 | 0.030 | 0.295 | 0.108 |
| *Actinomyces sp. 299* | 0.127 | 0.134 | 0.056 | 0.107 |
