# Supplementary figures and images for: The oral microbiota is a reservoir for antimicrobial resistance: resistome and phenotypic resistance characteristics of oral biofilm in health, caries, and periodontitis
Source: Ann Clin Microbiol Antimicrob. 2023 May 13;22:37. doi: 10.1186/s12941-023-00585-z (PMC10183135; doi:10.1186/s12941-023-00585-z)

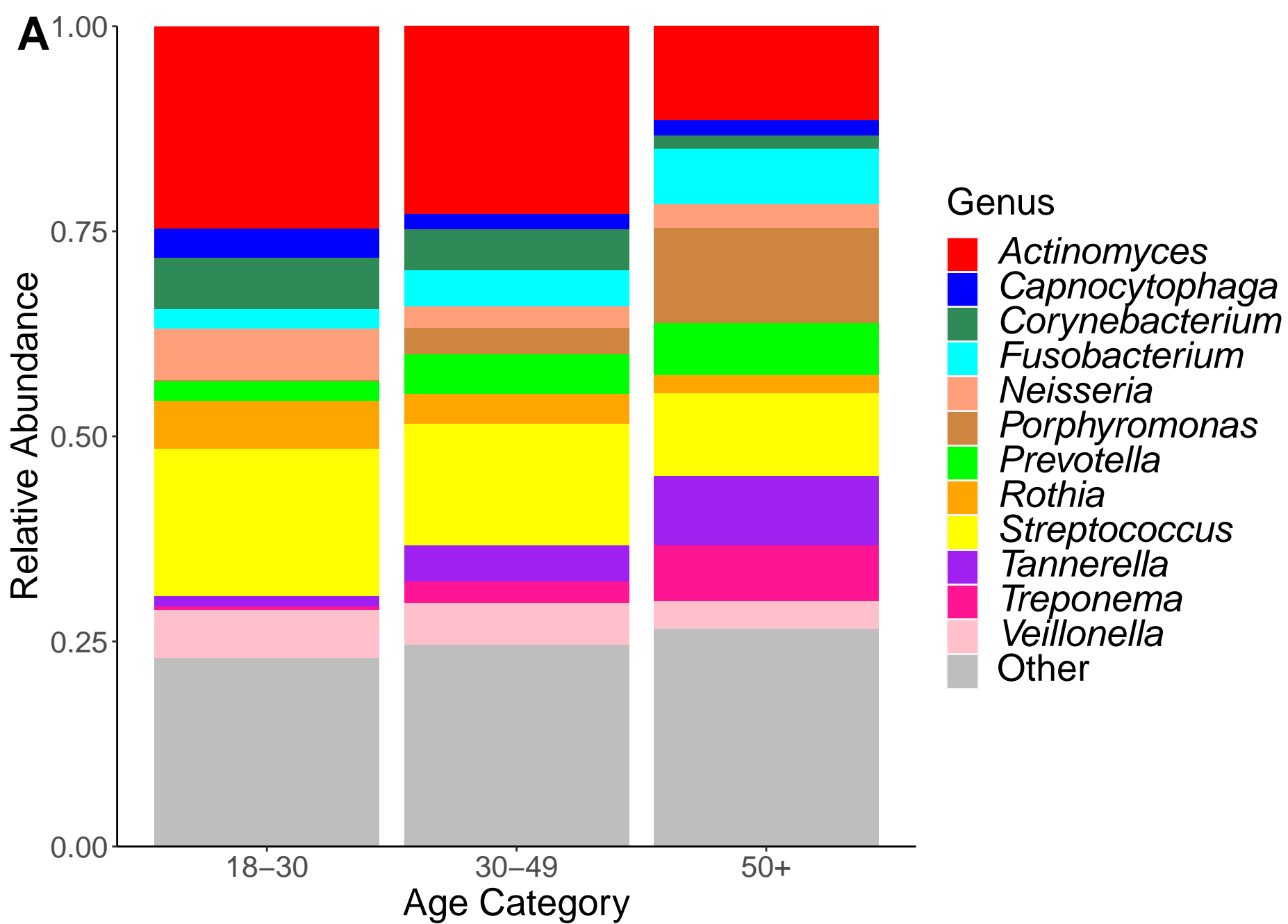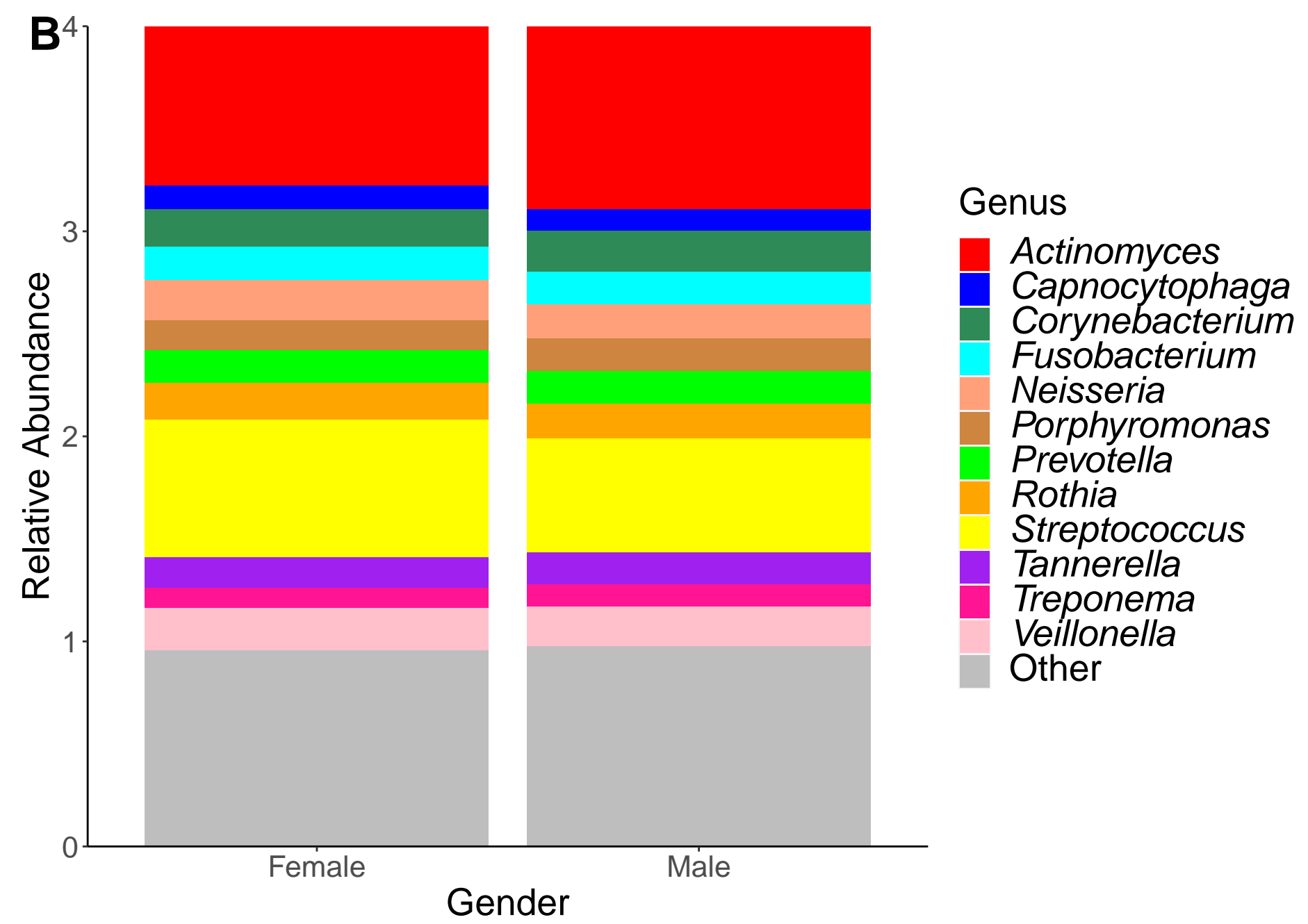

Supplement: Supplementary file 2 — Additional file 2: Figure S1. Bacterial composition of supragingivaland subgingivalbiofilm samples of 179 study participants based on metagenomic sequencing results. The 12 most abundant generaare shown A) Mean relative abundances in percent in three age categories; B) Mean relative abundances in percent in males and females. Healthy, Caries, and Periodontitis. [file 12941_2023_585_MOESM2_ESM.pdf]

**A**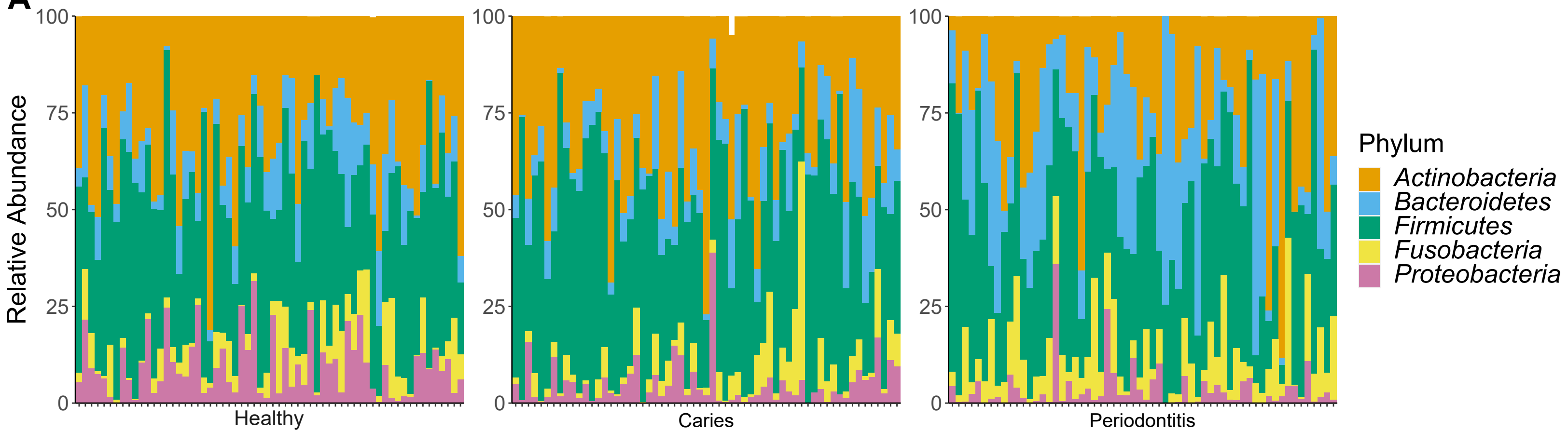**B**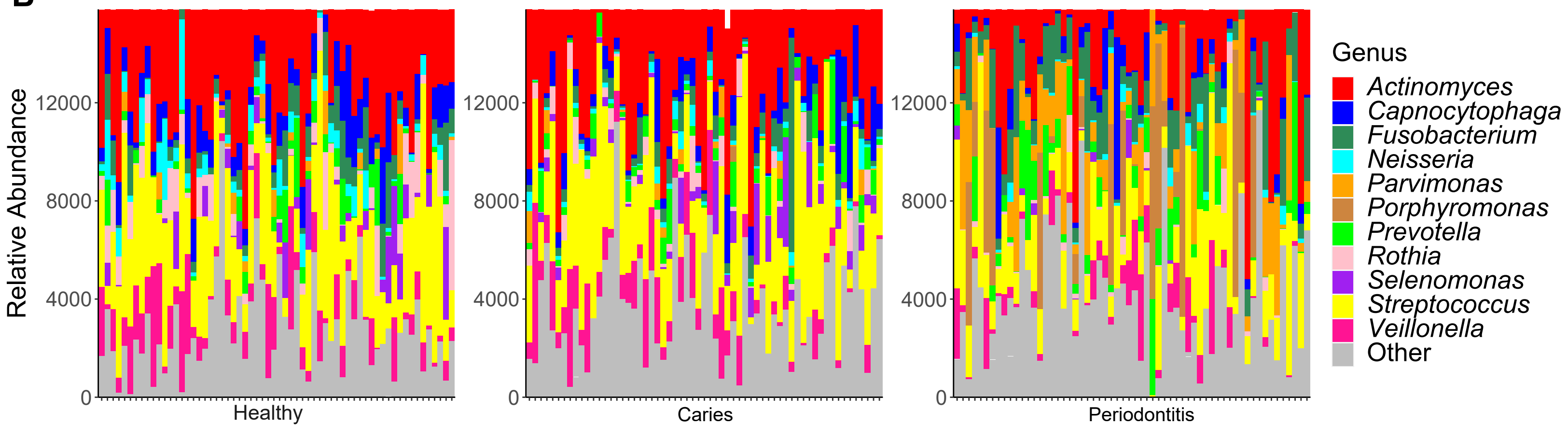**C**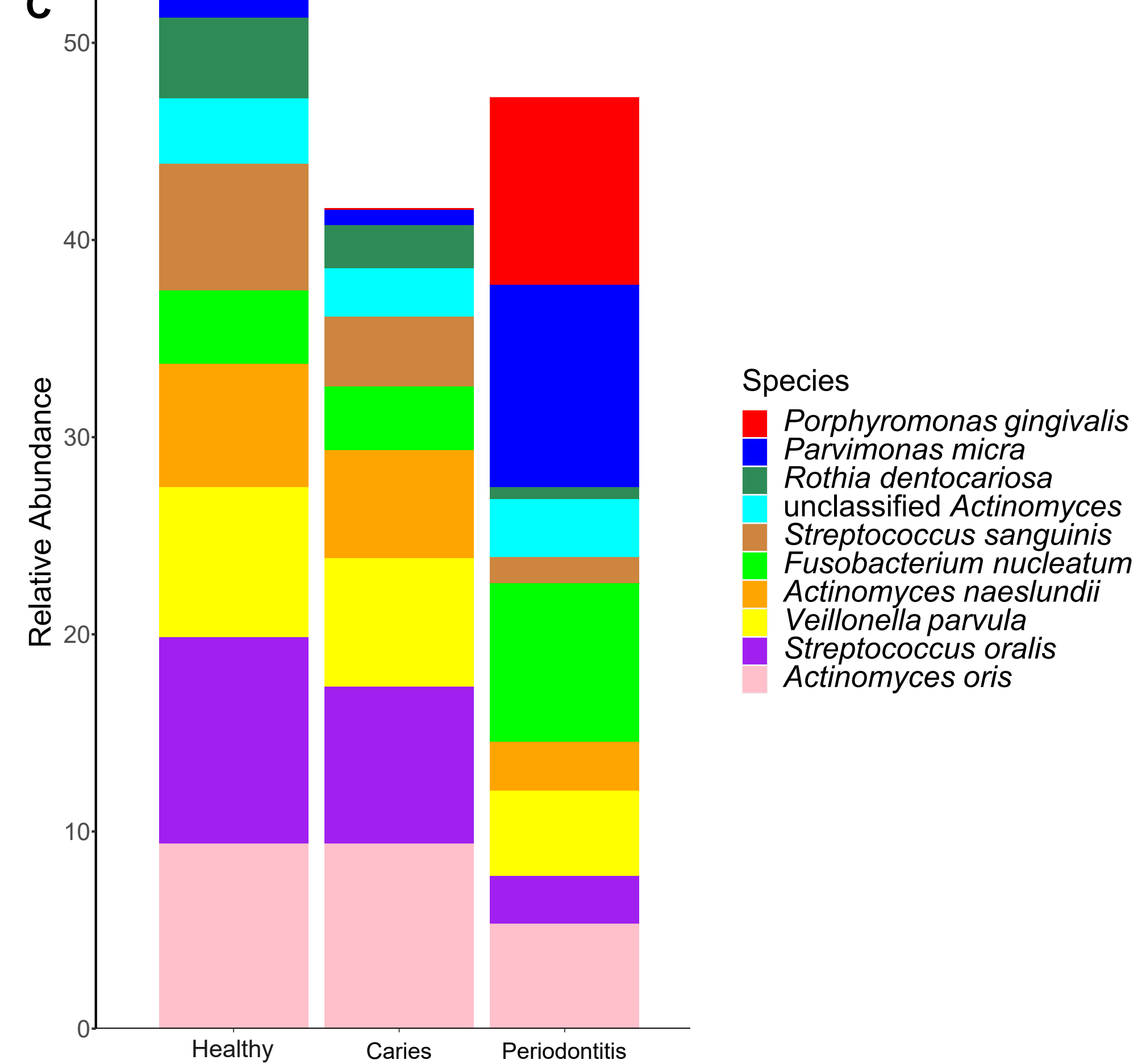

**A**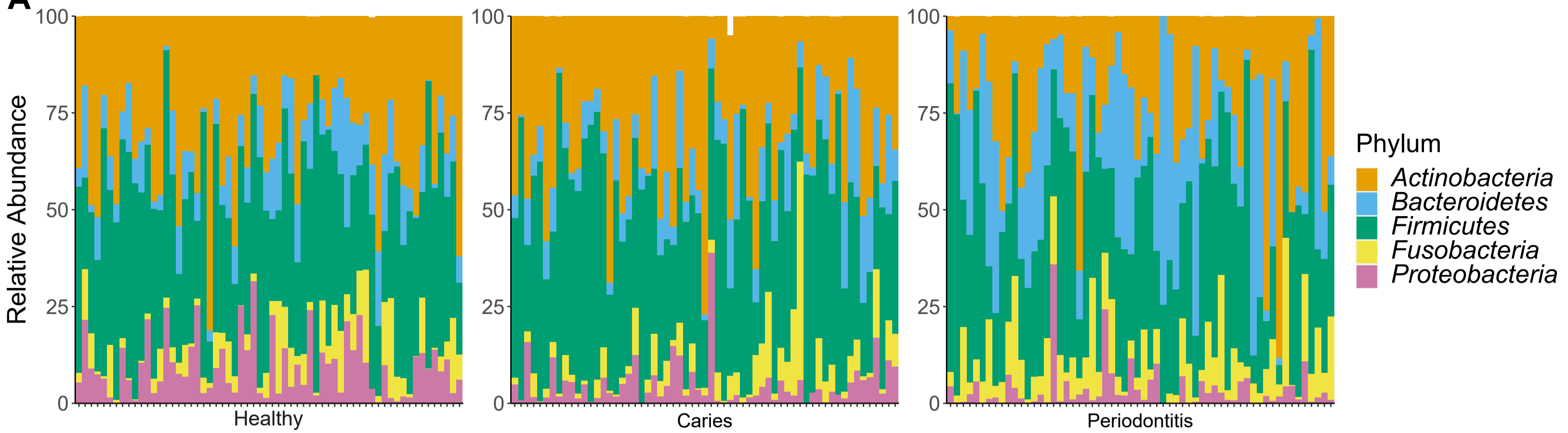**B**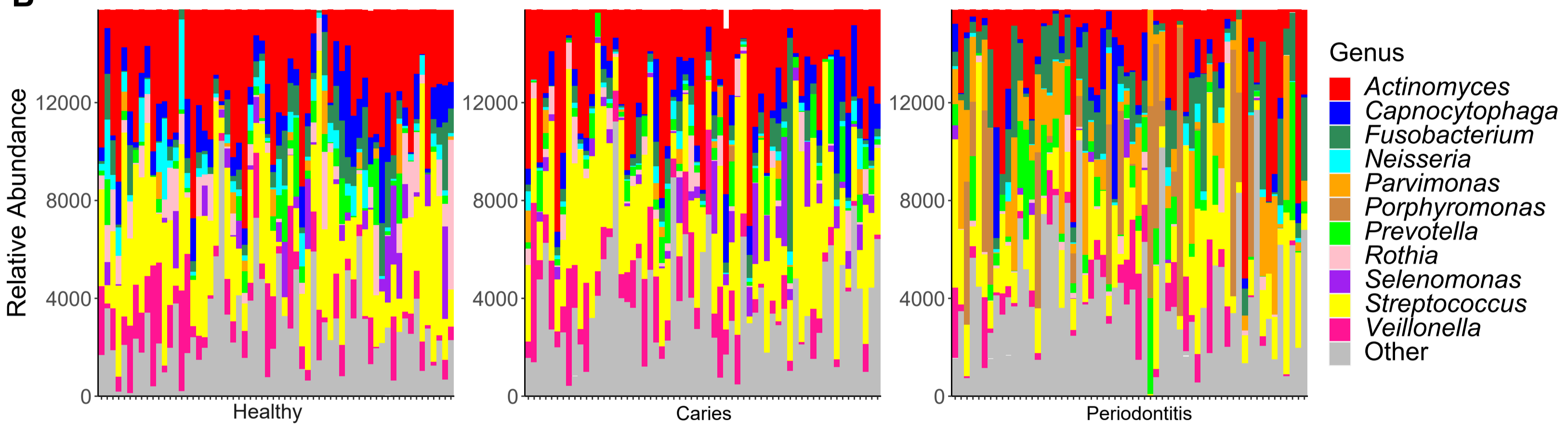

Supplement: Supplementary file 3 — Additional file 3: Figure S2. Bacterial composition of supragingivaland subgingivalbiofilm samples of 179 study participants based on culture technique as bacterial count in % CFU. A) Bacterial count of microbial phyla in H,C,P; B) Bacterial count of microbial genera in H,C,P; C) Bacterial count of 10 most abundant microbial species in H,C,P. Healthy, Caries, and Periodontitis. [file 12941_2023_585_MOESM3_ESM.pdf]

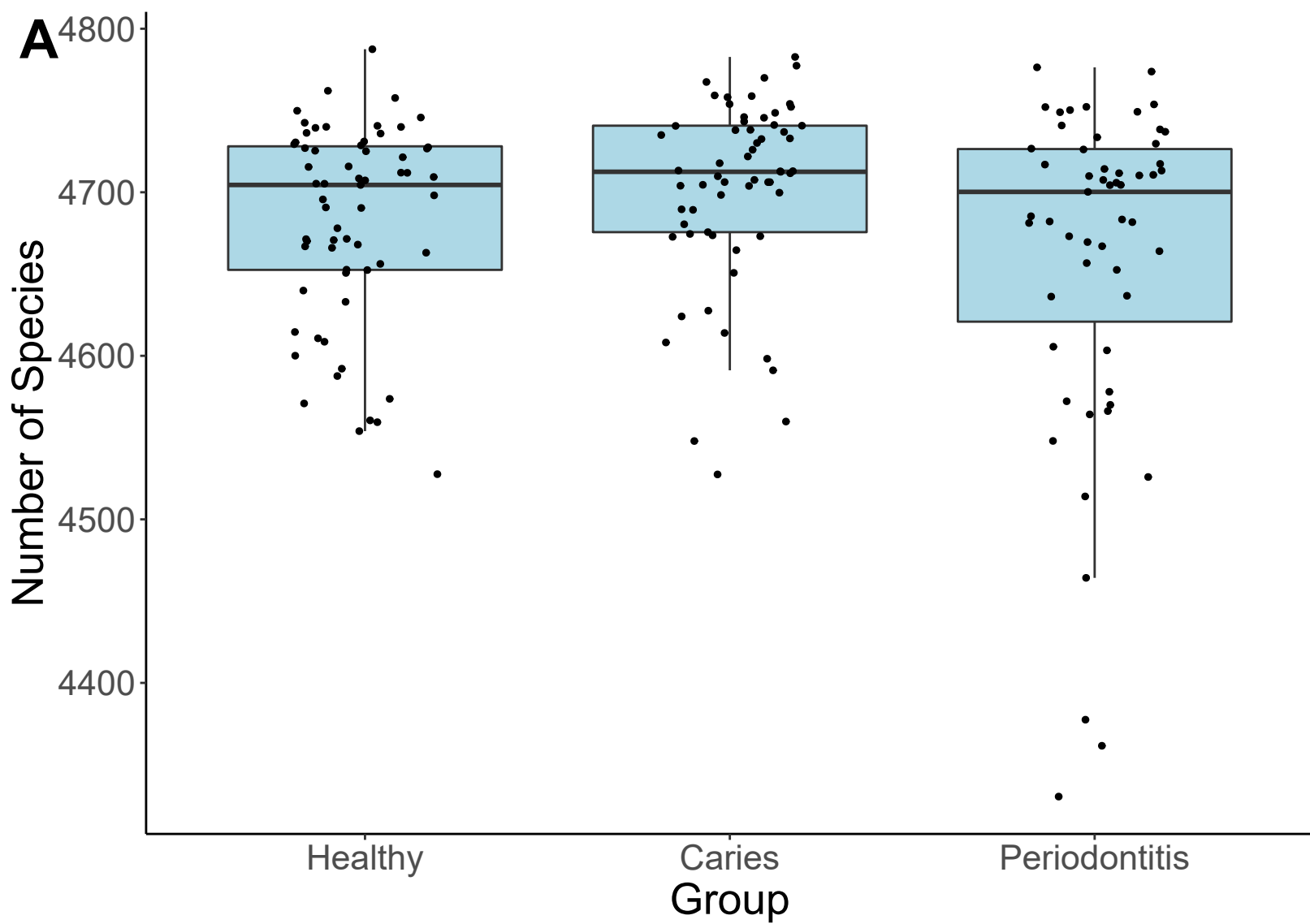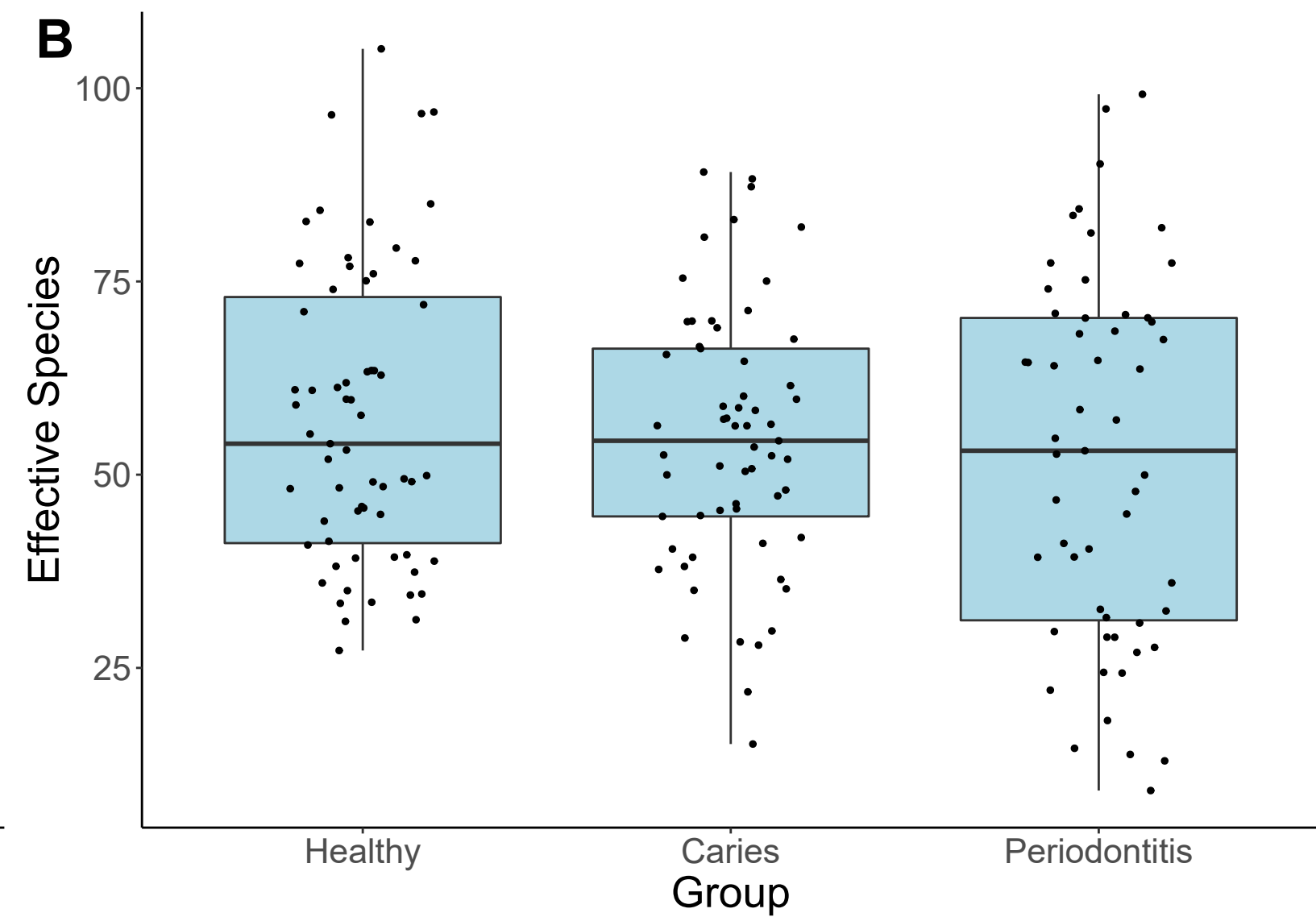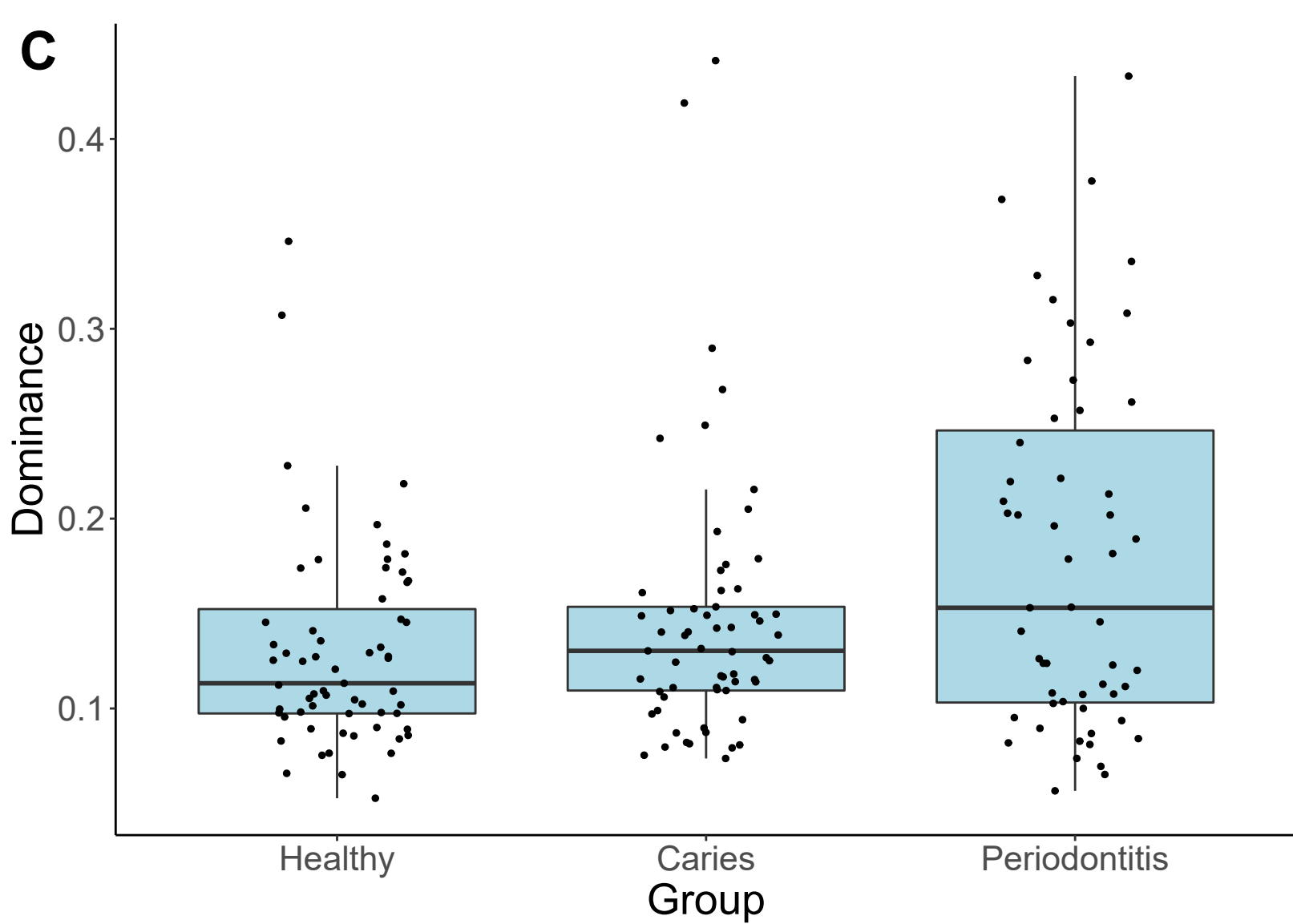

Supplement: Supplementary file 4 — Additional file 4: Figure S3. Diversity measures of the microbial composition in biofilm samples of 179 study participants based on metagenomic sequencing. The differences in A) Species richness, B) Alpha diversity; and C) dominance between the three groups with different oral conditions. Healthy, Caries, and Periodontitis. [file 12941_2023_585_MOESM4_ESM.pdf]

A

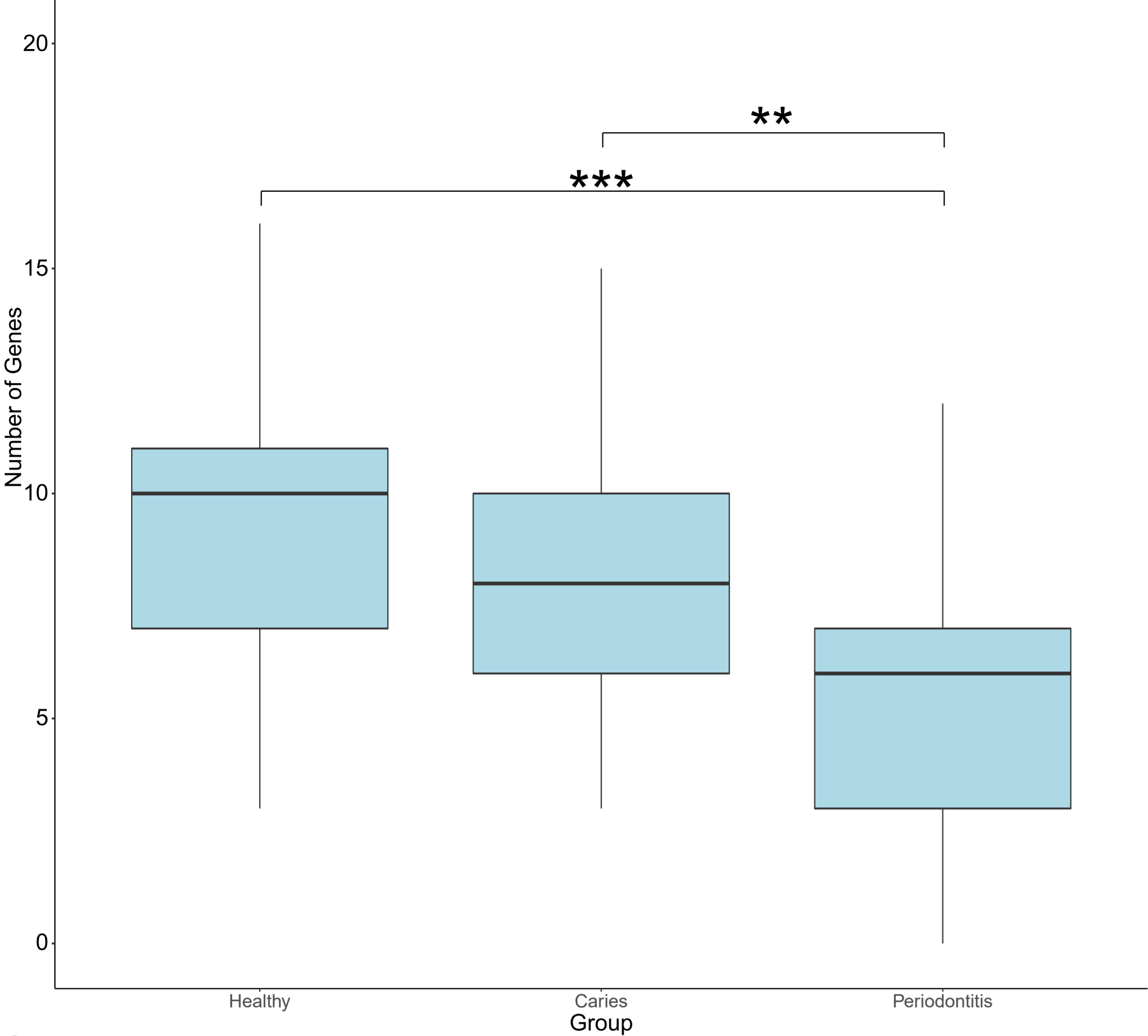

B

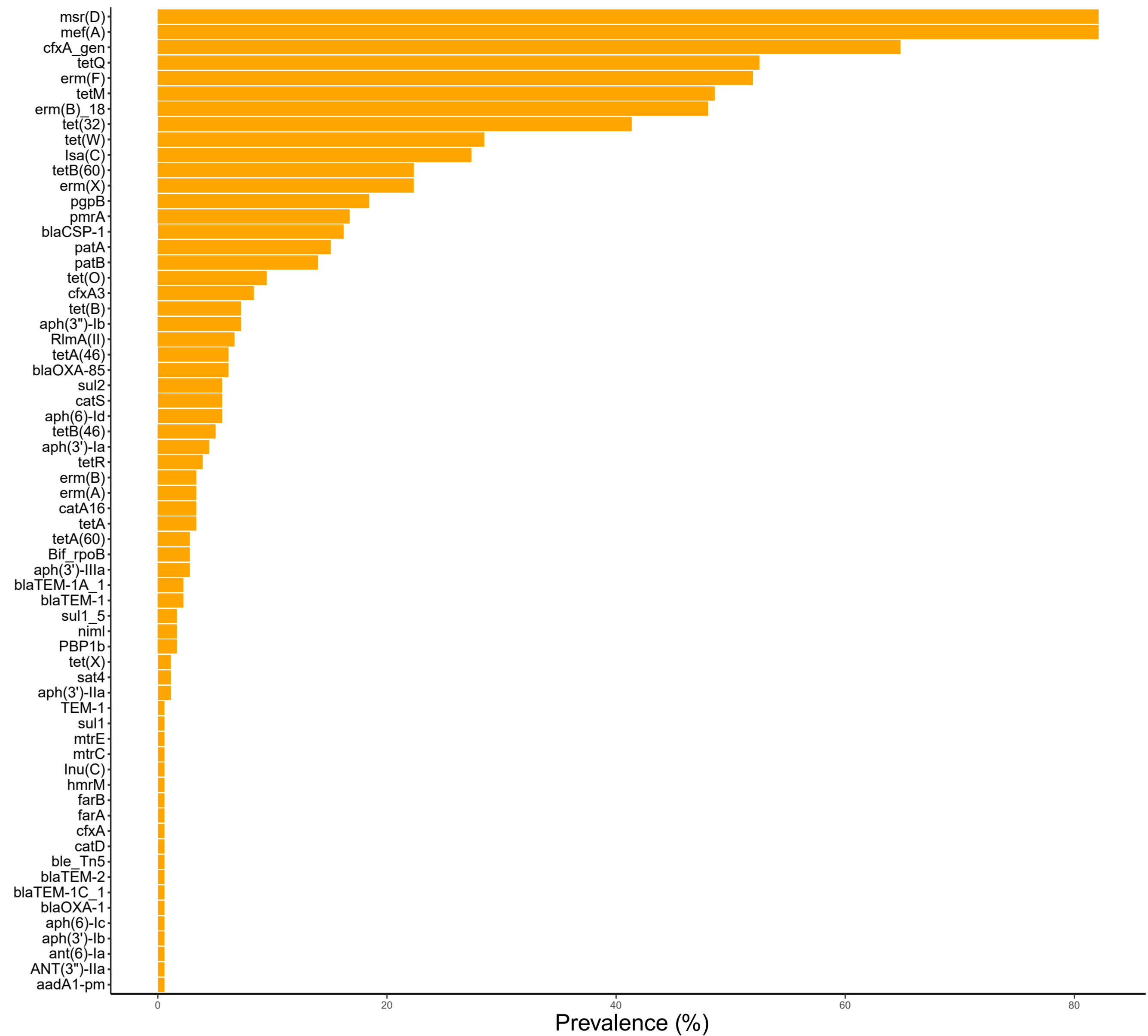

C

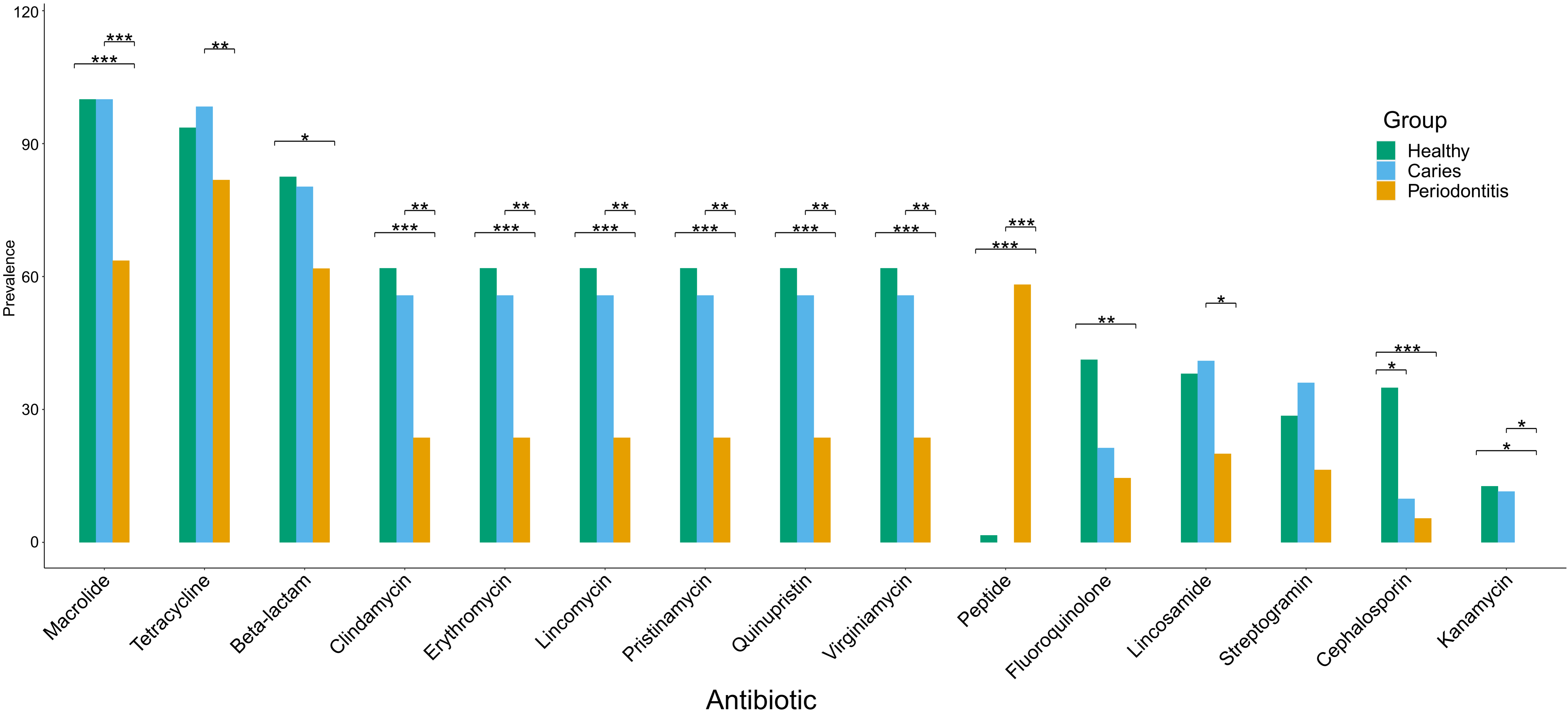



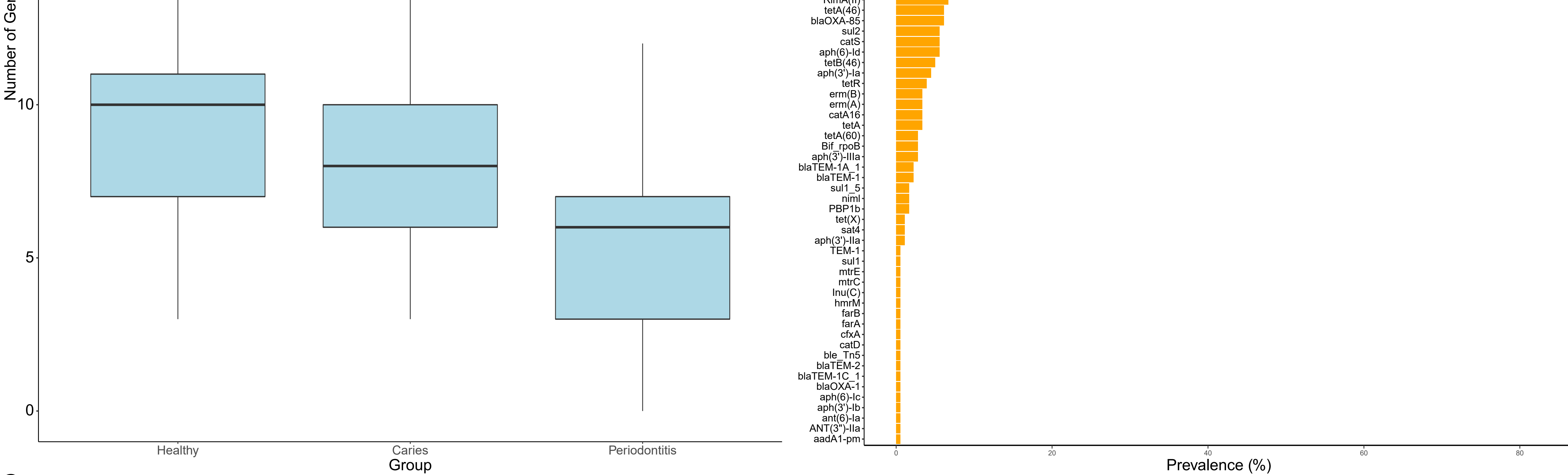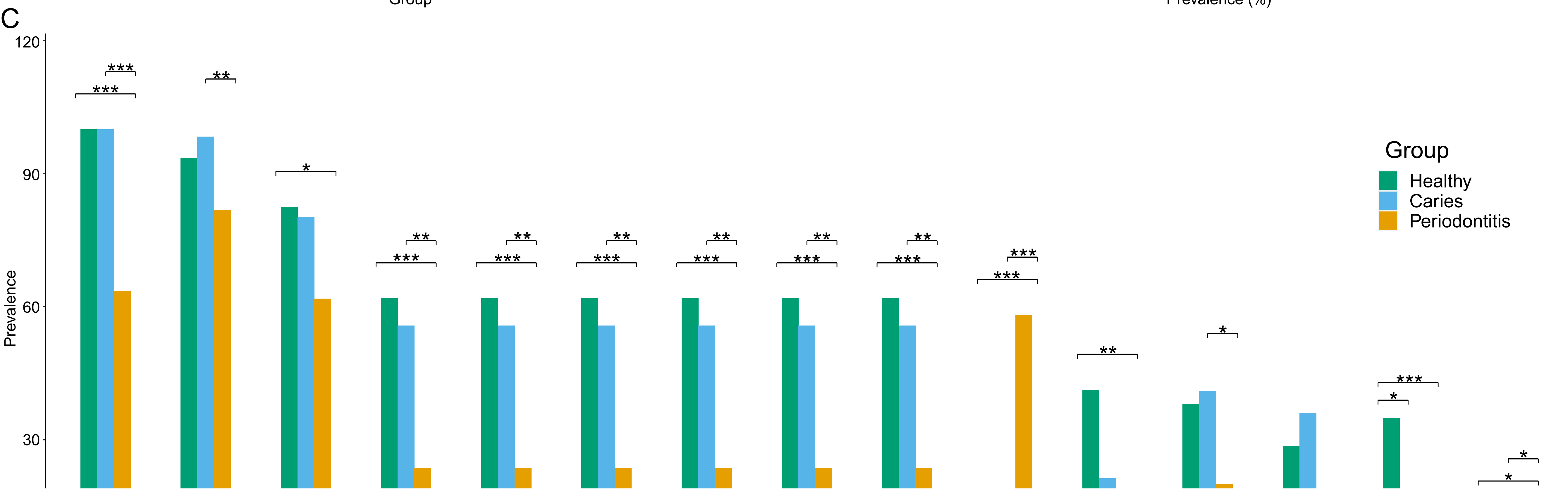

Supplement: Supplementary file 5 — Additional file 5: Figure S4. Detection of antibiotic resistance genesin oral biofilm samples of three different groupsof 179 study participants based on metagenomic sequencing. A) Numbers of ARGs present in samples from H,C,P; B) Prevalence of the ARGs across all samples; C) Prevalence of resistance to the different antibiotics/ antibiotic classes in H,C,P with significance determined using Firth’s logistic regression. * <0.05; **<0.01; ***<0.001. Healthy, Caries, and Periodontitis. [file 12941_2023_585_MOESM5_ESM.pdf]

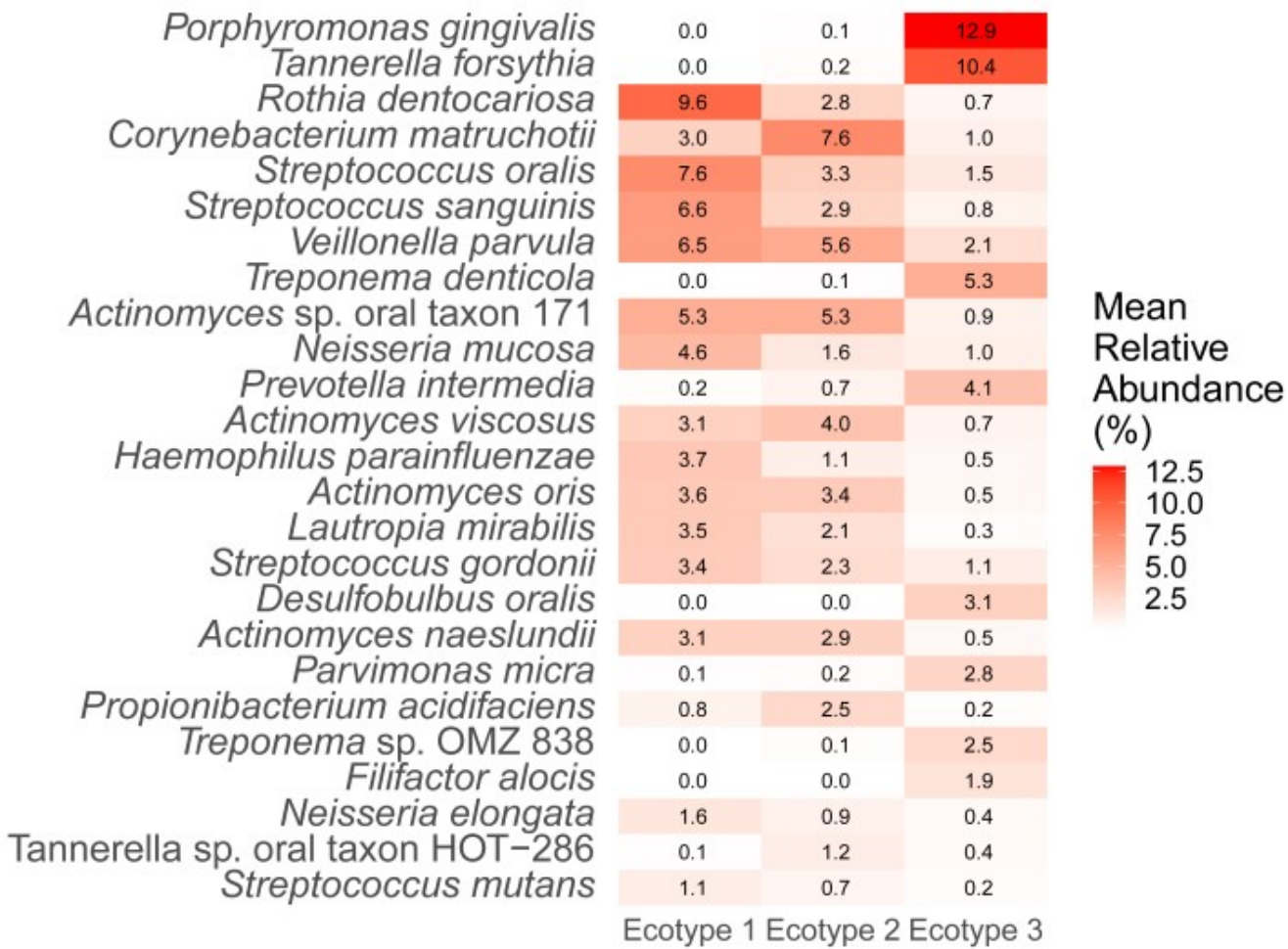

Supplement: Supplementary file 6 — Additional file 6: Figure S5. Bacterial taxa underlying the 3 ecotypesfound in oral biofilm samples of three different groupsof 179 study participants based on metagenomic sequencing. Healthy, Caries, and Periodontitis. [file 12941_2023_585_MOESM6_ESM.pdf]
