# Supplementary material for: The oral microbiota is a reservoir for antimicrobial resistance: resistome and phenotypic resistance characteristics of oral biofilm in health, caries, and periodontitis
Source: Ann Clin Microbiol Antimicrob. 2023 May 13;22:37. doi: 10.1186/s12941-023-00585-z (PMC10183135; doi:10.1186/s12941-023-00585-z)
Supplement: Supplementary file 7 — Additional file 7: Figure S6. Comparison of phenotypic resistanceand genotypic resistancein oral biofilm samples of 179 study participants. The agreement between the phenotypic and sequencing methods in terms of whether each of the species tested phenotypically provides resistance to each of the tested antibiotics. Phenotype Resistance: only phenotypic resistance found; Match: percentage of samples where both methods agreed on resistance; Genotype Resistance: only genotypic resistance found; Phenotype Missing: species was not tested phenotypically; Genotype Missing: species was not found with sequencing; Missing in both: species resp. resistance was not found with either method. [file 12941_2023_585_MOESM7_ESM.pdf]

Ampicillin:

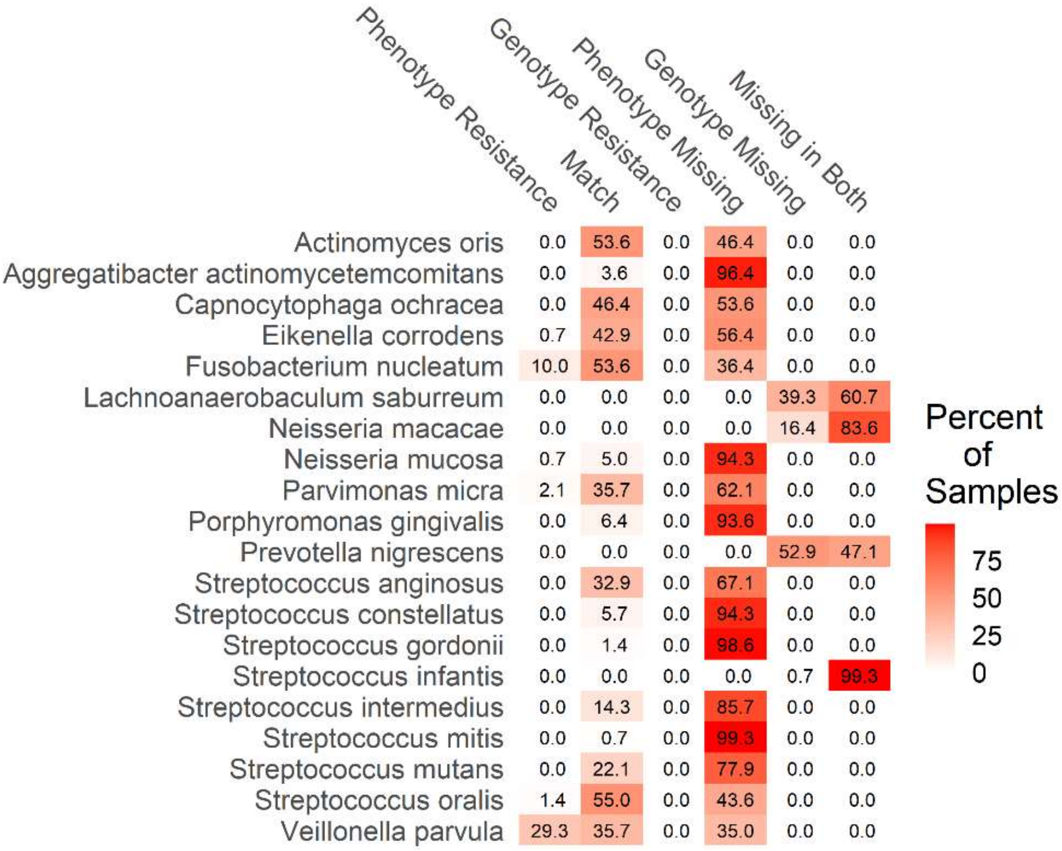

Azithromycin:

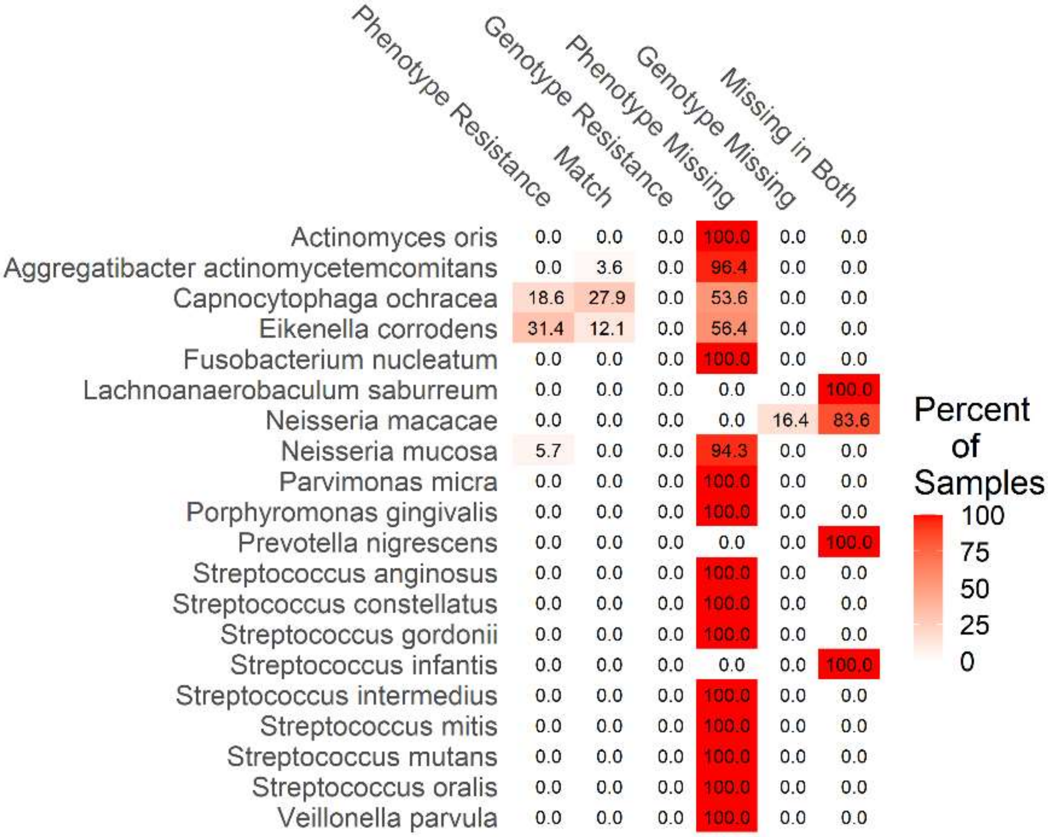

Cefuroxim:

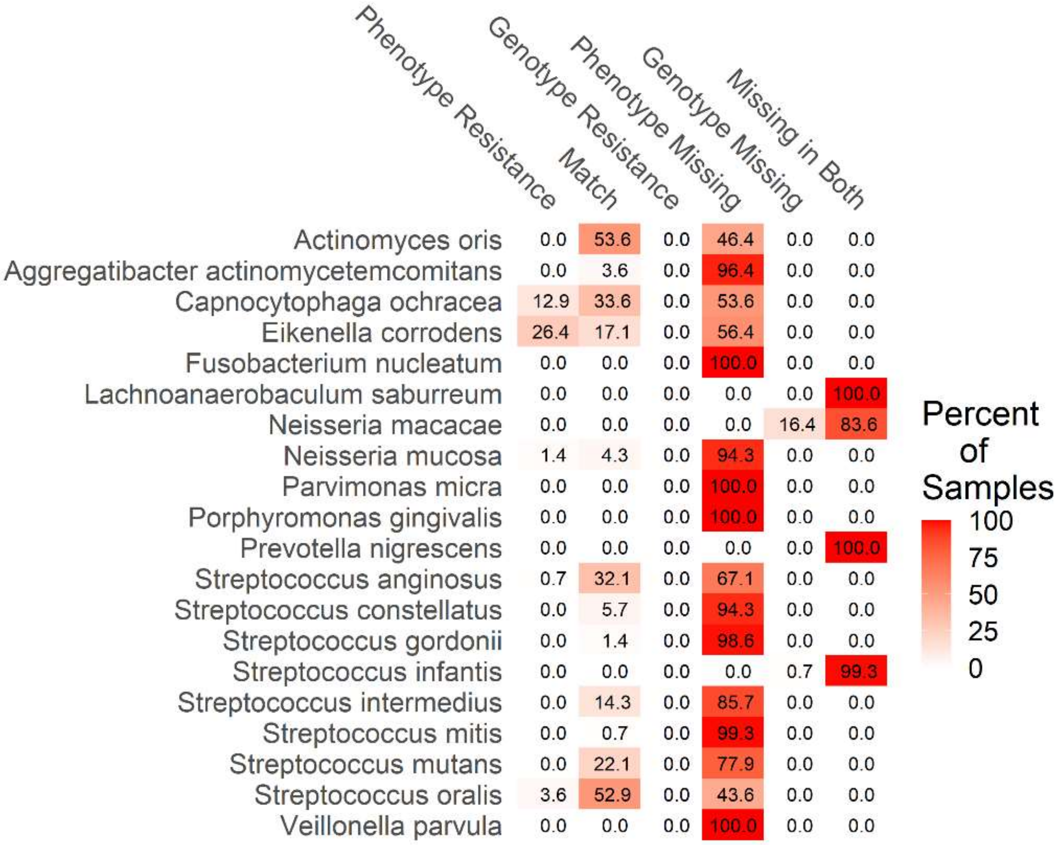

Ciprofloxacin:

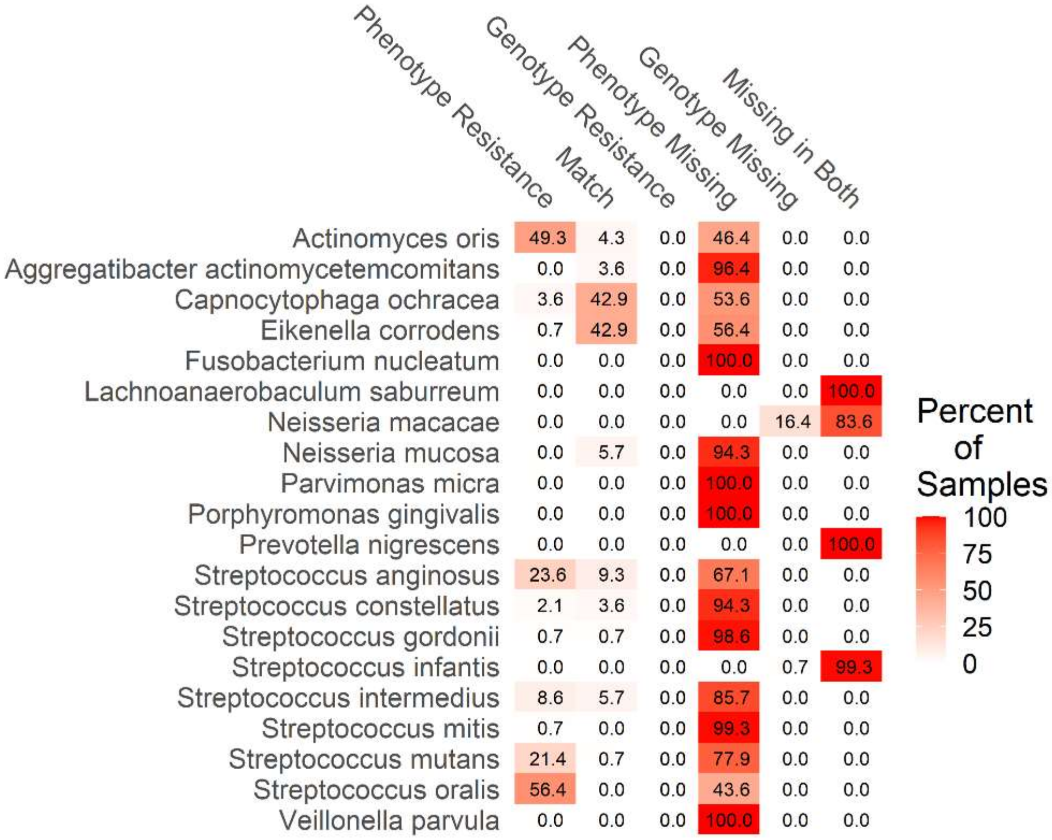

Clindamycin:

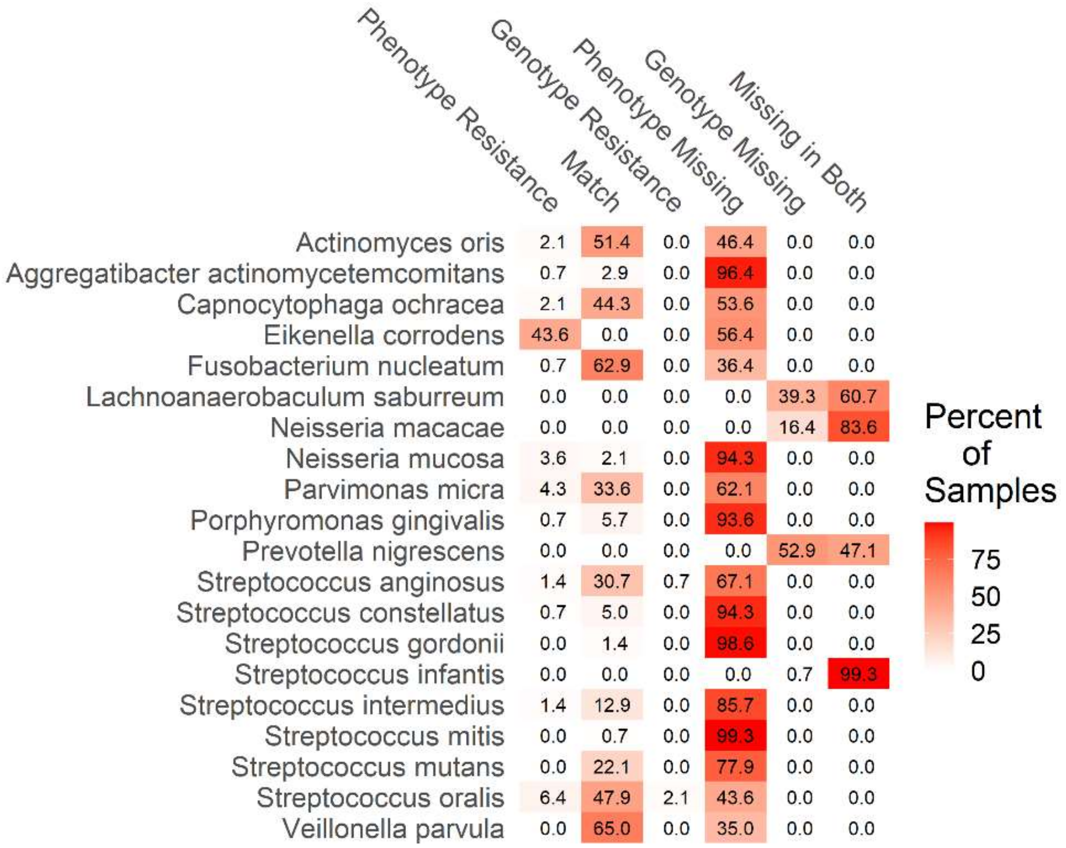

Colistin:

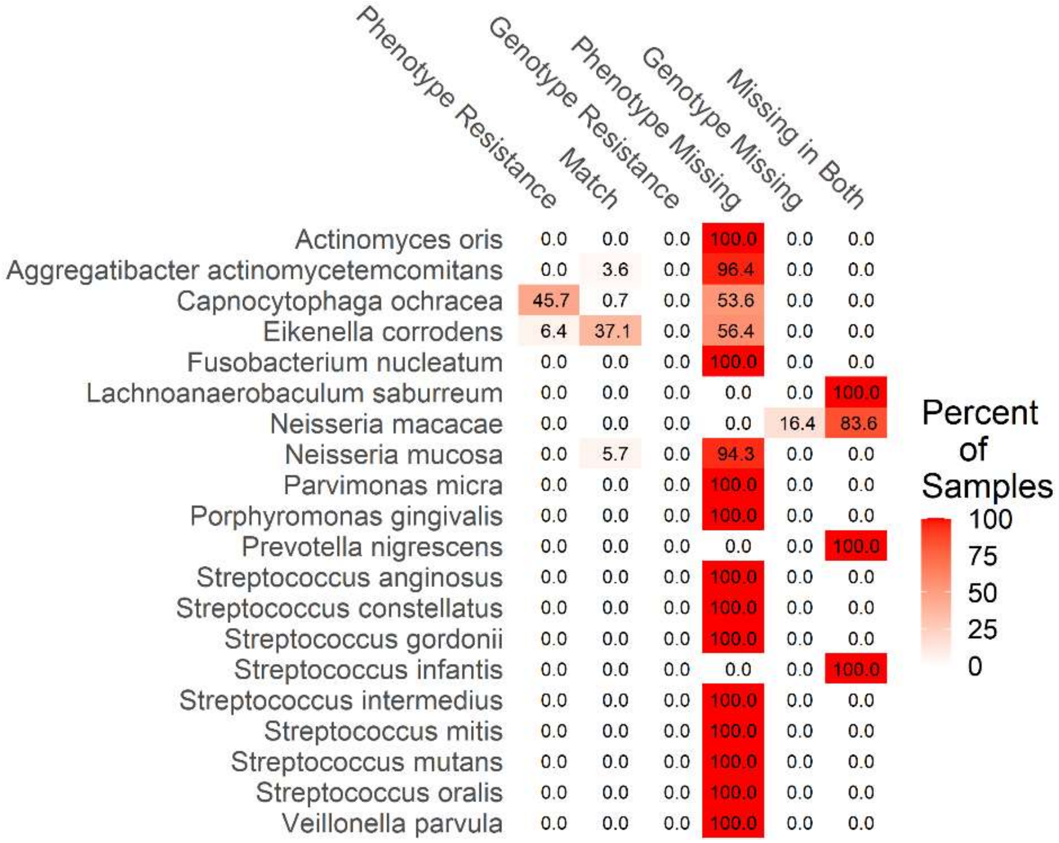

Erythromycin:

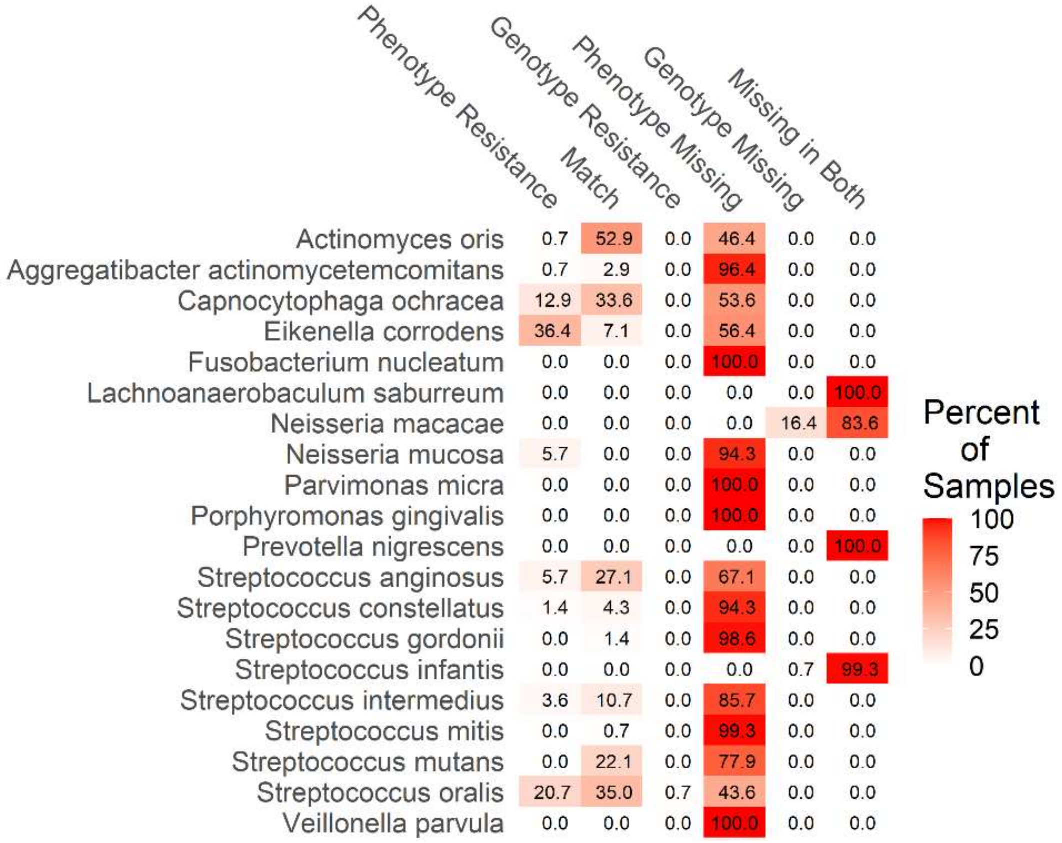

Fosfomycin:

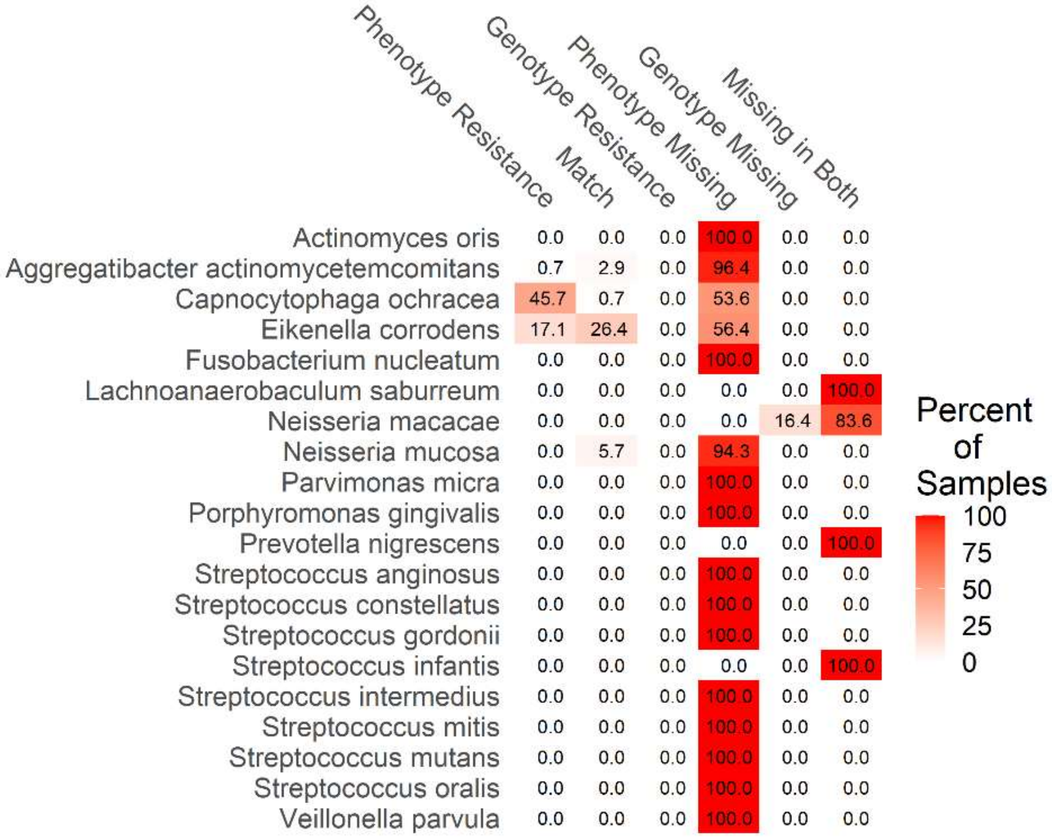

Gentamycin:

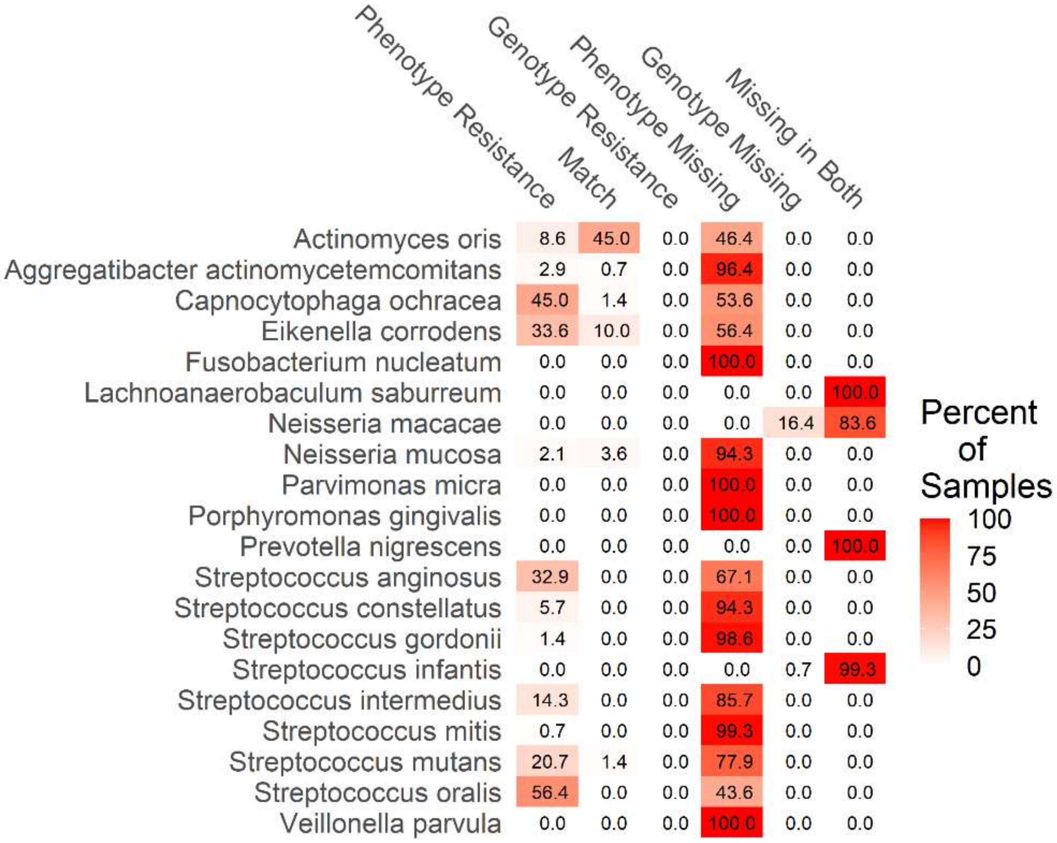

Meropenem:

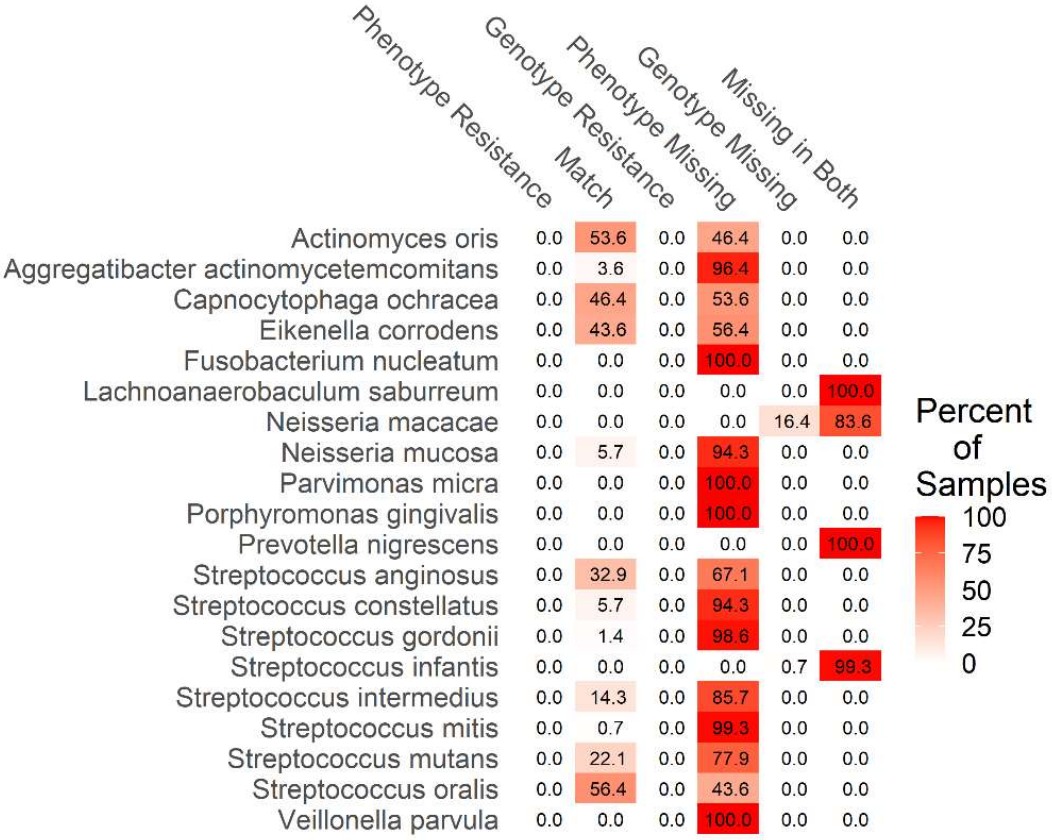

Metronidazol:

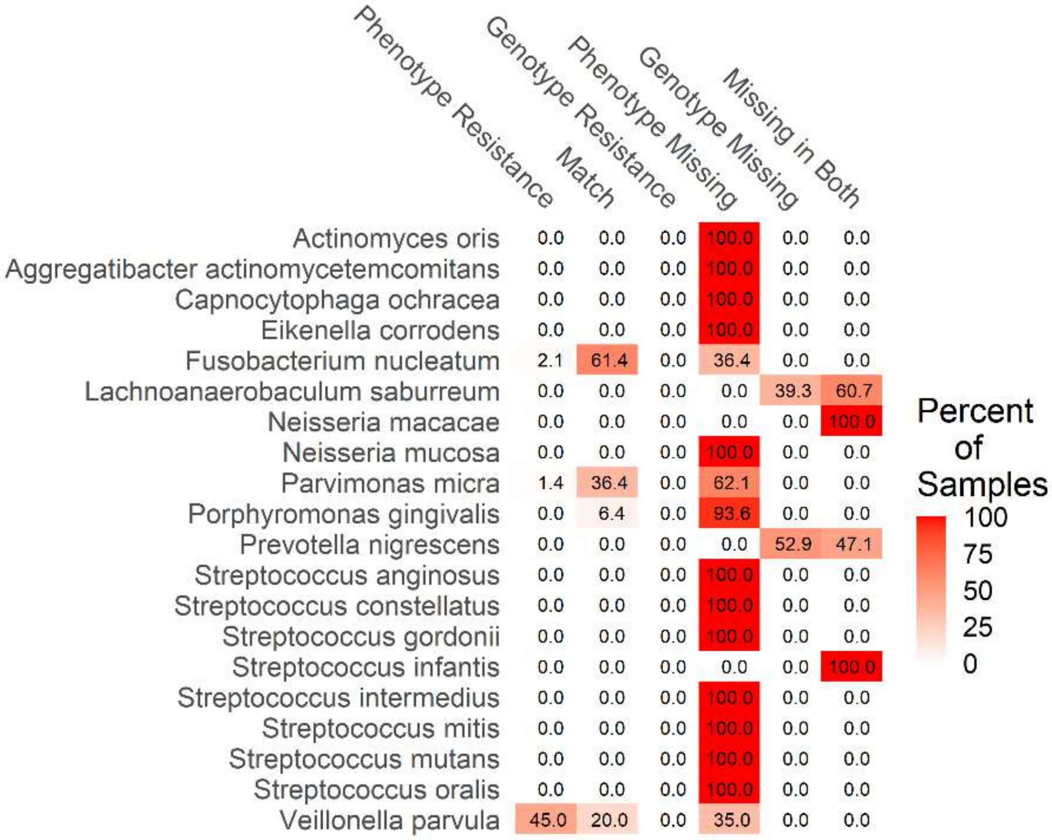

Moxifloxacin:

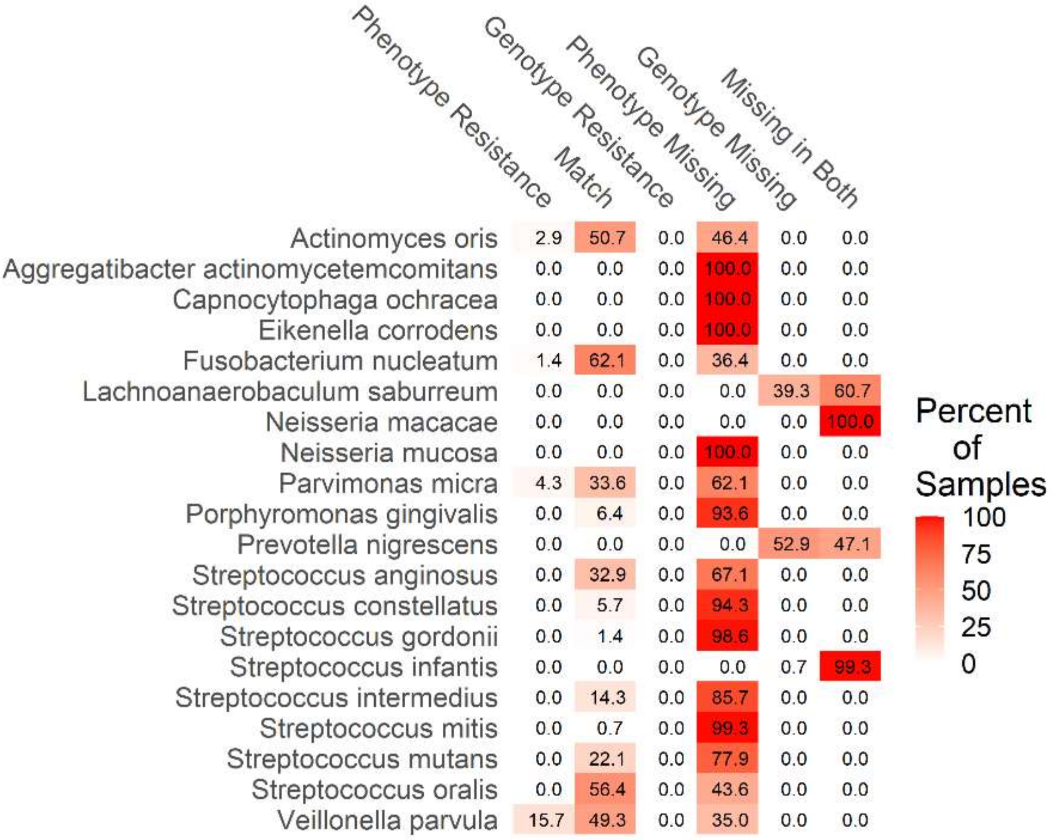

Penicillin G:

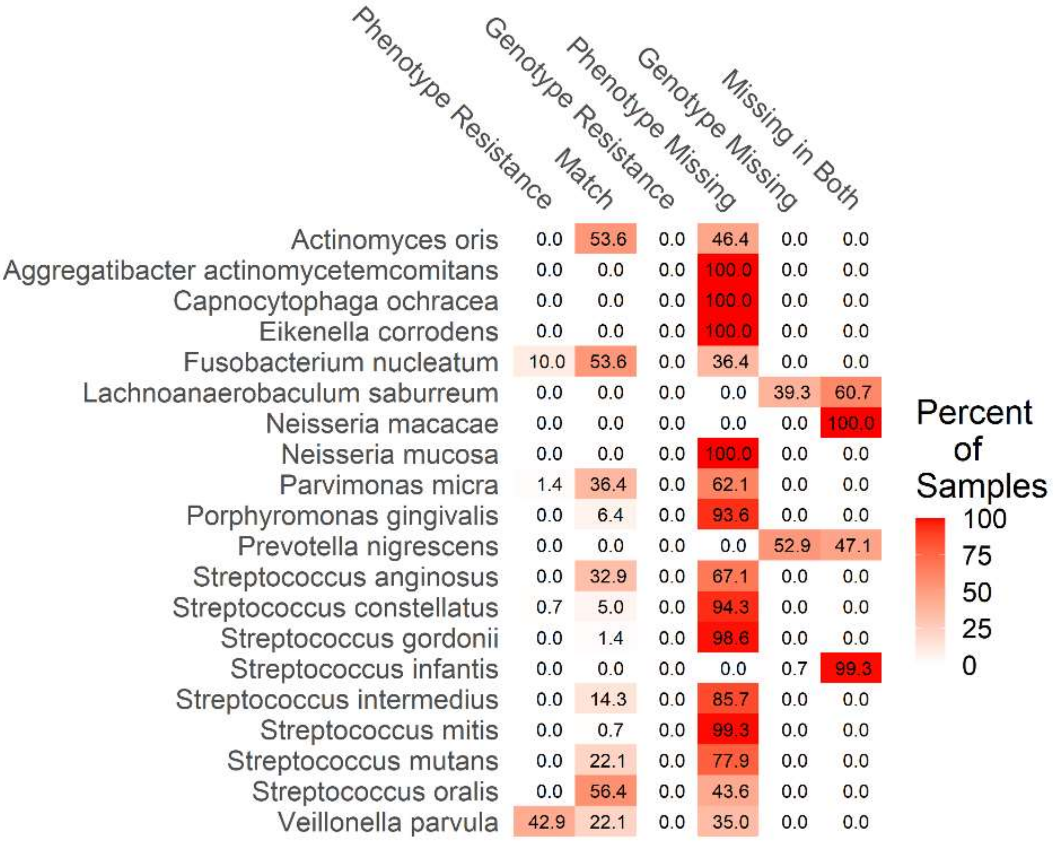

Tetracycline:

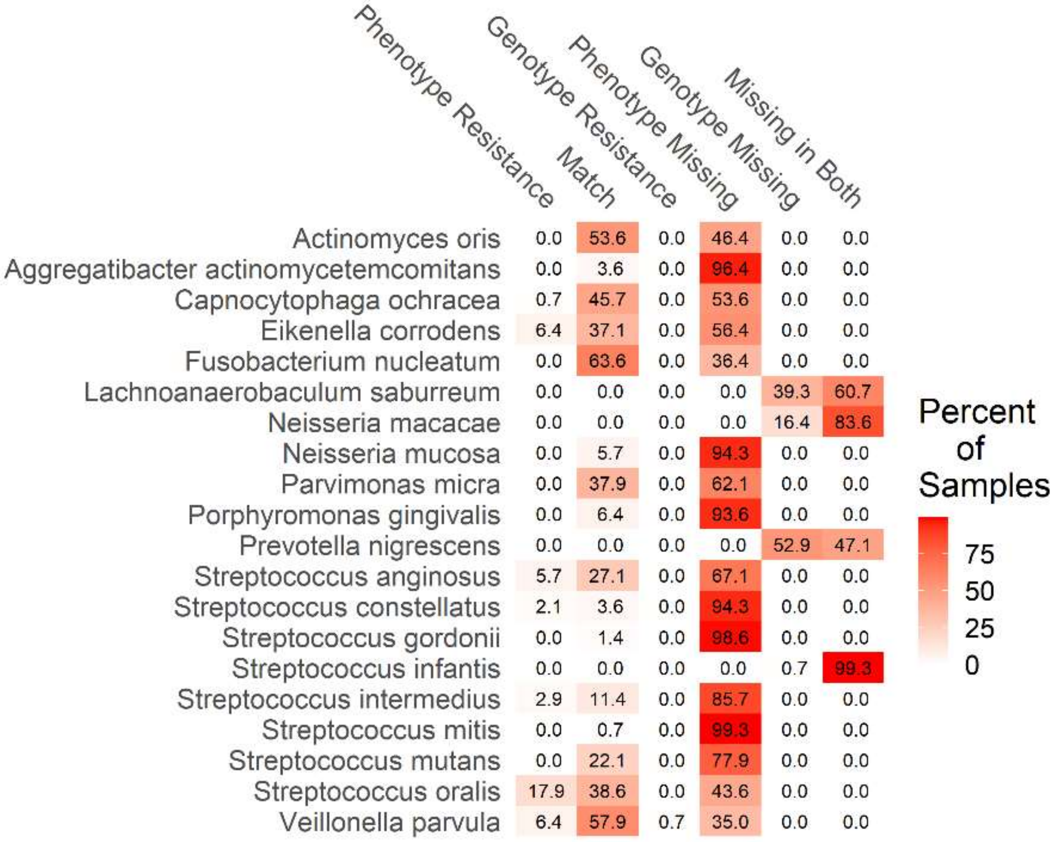

Tigecycline:

	Phenotype Resistance	Match	Genotype Resistance	Phenotype Missing	Genotype Missing	Missing in Both
Actinomyces oris	0.0	53.6	0.0	46.4	0.0	0.0
Aggregatibacter actinomycetemcomitans	0.0	3.6	0.0	96.4	0.0	0.0
Capnocytophaga ochracea	0.7	45.7	0.0	53.6	0.0	0.0
Eikenella corrodens	8.6	35.0	0.0	56.4	0.0	0.0
Fusobacterium nucleatum	0.0	0.0	0.0	100.0	0.0	0.0
Lachnoanaerobaculum saburreum	0.0	0.0	0.0	0.0	0.0	100.0
Neisseria macacae	0.0	0.0	0.0	0.0	16.4	83.6
Neisseria mucosa	0.0	5.7	0.0	94.3	0.0	0.0
Parvimonas micra	0.0	0.0	0.0	100.0	0.0	0.0
Porphyromonas gingivalis	0.0	0.0	0.0	100.0	0.0	0.0
Prevotella nigrescens	0.0	0.0	0.0	0.0	0.0	100.0
Streptococcus anginosus	0.0	32.9	0.0	67.1	0.0	0.0
Streptococcus constellatus	0.0	5.7	0.0	94.3	0.0	0.0
Streptococcus gordonii	0.0	1.4	0.0	98.6	0.0	0.0
Streptococcus infantis	0.0	0.0	0.0	0.0	0.7	99.3
Streptococcus intermedius	0.0	14.3	0.0	85.7	0.0	0.0
Streptococcus mitis	0.0	0.7	0.0	99.3	0.0	0.0
Streptococcus mutans	0.0	22.1	0.0	77.9	0.0	0.0
Streptococcus oralis	0.0	56.4	0.0	43.6	0.0	0.0
Veillonella parvula	0.0	0.0	0.0	100.0	0.0	0.0

Percent of Samples: 100, 75, 50, 25, 0

Vancomycin:

	Phenotype Resistance	Match	Genotype Resistance	Phenotype Missing	Genotype Missing	Missing in Both
Actinomyces oris	0.0	53.6	0.0	46.4	0.0	0.0
Aggregatibacter actinomycetemcomitans	0.0	0.0	0.0	100.0	0.0	0.0
Capnocytophaga ochracea	0.0	0.0	0.0	100.0	0.0	0.0
Eikenella corrodens	0.0	0.0	0.0	100.0	0.0	0.0
Fusobacterium nucleatum	0.0	0.0	0.0	100.0	0.0	0.0
Lachnoanaerobaculum saburreum	0.0	0.0	0.0	0.0	0.0	100.0
Neisseria macacae	0.0	0.0	0.0	0.0	0.0	100.0
Neisseria mucosa	0.0	0.0	0.0	100.0	0.0	0.0
Parvimonas micra	0.0	0.0	0.0	100.0	0.0	0.0
Porphyromonas gingivalis	0.0	0.0	0.0	100.0	0.0	0.0
Prevotella nigrescens	0.0	0.0	0.0	0.0	0.0	100.0
Streptococcus anginosus	0.0	32.9	0.0	67.1	0.0	0.0
Streptococcus constellatus	0.7	5.0	0.0	94.3	0.0	0.0
Streptococcus gordonii	0.0	1.4	0.0	98.6	0.0	0.0
Streptococcus infantis	0.0	0.0	0.0	0.0	0.7	99.3
Streptococcus intermedius	0.0	14.3	0.0	85.7	0.0	0.0
Streptococcus mitis	0.0	0.7	0.0	99.3	0.0	0.0
Streptococcus mutans	0.0	22.1	0.0	77.9	0.0	0.0
Streptococcus oralis	0.0	56.4	0.0	43.6	0.0	0.0
Veillonella parvula	0.0	0.0	0.0	100.0	0.0	0.0

Percent of Samples: 100, 75, 50, 25, 0
